# An Immunoinformatics Approach to Design a Potent Multi-Epitope Vaccine against Asia-1 Genotype of Crimean–Congo Haemorrhagic Fever Virus Using the Structural Glycoproteins as a Target

**DOI:** 10.3390/vaccines11010061

**Published:** 2022-12-27

**Authors:** Syed Zawar Shah, Basit Jabbar, Muhammad Usman Mirza, Muhammad Waqas, Shahkaar Aziz, Sobia Ahsan Halim, Amjad Ali, Shazia Rafique, Muhammad Idrees, Asaad Khalid, Ashraf N. Abdalla, Ajmal Khan, Ahmed Al-Harrasi

**Affiliations:** 1 Centre of Excellence in Molecular Biology, University of the Punjab, Lahore 53700, Pakistan; 2 Department of Chemistry and Biochemistry, University of Windsor, Windsor, ON N9B 3P4, Canada; 3 Department of Biotechnology and Genetic Engineering, Hazara University Mansehra, Mansehra 21120, Pakistan; 4 Natural and Medical Sciences Research Center, University of Nizwa, Birkat-ul-Mouz 616, Oman; 5 Institute of Biotechnology and Genetic Engineering, The University of Agriculture, Peshawar 25130, Pakistan; 6 Substance Abuse and Toxicology Research Center, Jazan University, P.O. Box 114, Jazan 45142, Saudi Arabia; 7 Medicinal and Aromatic Plants and Traditional Medicine Research Institute, National Center for Research, P.O. Box 2404, Khartoum 11111, Sudan; 8 Department of Pharmacology and Toxicology, College of Pharmacy, Umm Al-Qura University, Makkah 21955, Saudi Arabia

**Keywords:** epitope-based vaccine, immunoinformatics, multi-epitope vaccine, epitopes prediction, vaccine design, Crimean–Congo haemorrhagic fever

## Abstract

Crimean–Congo haemorrhagic fever (CCHF), caused by Crimean–Congo haemorrhagic fever virus (CCHFV), is a disease of worldwide importance (endemic yet not limited to Asia, Middle East, and Africa) and has triggered several outbreaks amounting to a case fatality rate of 10–40% as per the World Health Organization. Genetic diversity and phylogenetic data revealed that the Asia-1 genotype of CCHFV remained dominant in Pakistan, where 688 confirmed cases were reported between the 2012–2022 period. Currently, no approved vaccine is available to tackle the viral infection. Epitope-based vaccine design has gained significant attention in recent years due to its safety, timeliness, and cost efficiency compared to conventional vaccines. In the present study, we employed a robust immunoinformatics-based approach targeting the structural glycoproteins G1 and G2 of CCHFV (Asia-1 genotype) to design a multi-epitope vaccine construct. Five B-cells and six cytotoxic T-lymphocytes (CTL) epitopes were mapped and finalized from G1 and G2 and were fused with suitable linkers (EAAAK, GGGS, AAY, and GPGPG), a PADRE sequence (13 aa), and an adjuvant (50S ribosomal protein L7/L12) to formulate a chimeric vaccine construct. The selected CTL epitopes showed high affinity and stable binding with the binding groove of common human HLA class I molecules (HLA-A*02:01 and HLA-B*44:02) and mouse major histocompatibility complex class I molecules. The chimeric vaccine was predicted to be an antigenic, non-allergenic, and soluble molecule with a suitable physicochemical profile. Molecular docking and molecular dynamics simulation indicated a stable and energetically favourable interaction between the constructed antigen and Toll-like receptors (TLR2, TLR3, and TLR4). Our results demonstrated that innate, adaptive, and humoral immune responses could be elicited upon administration of such a potent muti-epitope vaccine construct. These results could be helpful for an experimental vaccinologist to develop an effective vaccine against the Asia-1 genotype of CCHFV.

## 1. Introduction

Crimean–Congo haemorrhagic fever (CCHF) is caused by the human pathogenic agent Crimean–Congo haemorrhagic fever virus (CCHFV). It is known to have affected more than 30 countries and discrete areas around the world [1]. CCHFV belongs to the genus Nairovirus of the family Bunyaviridae; the nairoviruses are mainly tick-borne viruses [2]. The RNA genome of the Bunyaviridae family members harbours three negative-sense segments, S (small), M (medium), and L (large), which minimally code for the virus nucleocapsid (N), glycoprotein precursor (GPC), and polymerase (L) proteins, respectively [3]. Two structural glycoproteins (i.e., G1 and G2, also known as GN and GC, respectively) are encoded by the M segment [4,5].

The virus glycoproteins play a salient role in the natural tick–vertebrate cycle of CCHFV and are also responsible for high pathogenicity in humans. Identification of a highly variable mucin-like region at the amino terminus of the CCHFV glycoprotein precursor represents a distinct feature of nairoviruses belonging to the family Bunyaviridae [4]. As surface glycoproteins are important elements facilitating the interaction of CCHFV with its receptor, neutralizing antibodies are engendered against these proteins, making them an attractive target for designing potential vaccines against CCHFV [6]. Previous studies have reported that structural glycoproteins-based vaccines for CCHFV resulted in varying degrees of protection from viral infection in in vivo models [6,7,8,9].

CCHFV was initially reported in the 1940s among agricultural workers of the Crimean peninsula, where more than 200 cases of severe haemorrhagic fever were found [10]. Due to the severity of this disease, the fatality rate can rise to 30%. Its broad distribution around the world includes a significant part of the Middle East, Africa, Asia, and regions of Eastern Europe [11,12,13]. No vaccine is available to combat CCHFV infection in humans or animals [14]. The disease progresses rapidly in humans and results in acute febrile illness linked with petechiae, ecchymosis, disseminated intravascular coagulation, and multiple-organ failure [11]. CCHFV spreads throughout the body during the course of the disease, indicated by its detection in the spleen, lung, heart, and intestinal tissues in fatal cases [15]. Among the main cellular targets of CCHFV, infection of monocyte-derived macrophages, endothelial cells, and dendritic cells has been confirmed in vitro [16,17,18]. Owing to the error-prone polymerase, random mutations are incorporated into the single-stranded-RNA-containing genome of CCHFV. This, coupled with a high rate of recombination in CCHFV RNA, makes the development of a vaccine or antiviral drug against CCHFV onerous [19]. The efficacy of Ribavirin against CCHFV has been indicated in vitro and in animal models, but the clinical benefits of Ribavirin remain unwarranted [20].

Based on phylogenetic analysis, CCHFV is subdivided into seven distinct genetic classes: Africa 1–3, Asia 1–2, and Europe 1–2, which correlate to the geographic origin [3]. Since the detection of the first case in 1976, CCHF infection has been endemic in Pakistan. A total of 688 confirmed cases were reported between 2012–2022, with most cases reported from Pakistan’s Khyber Pakhtunkhwa and Baluchistan provinces. Genetic diversity and phylogenetic data revealed that the Asia-1 genotype of CCHFV remained dominant in Pakistan [21,22].

A multi-epitope vaccine composed of a collection of epitopes is a viable strategy for preventing and treating viral infection [23]. Besides, multi-epitope vaccines have the benefit of inducing humoral, innate, and cellular immune responses at the same time when compared to monovalent vaccines [24]. Traditional vaccine development procedures are time-consuming and labour-intensive [25]. Immunoinformatics-based tools, on the other hand, can evaluate the host immune response to present an alternative way for developing cost-effective vaccines against diseases because predictions can reduce the number of in vitro experiments required [26,27]. In addition, vaccines based on structural and non-structural proteins have been shown to induce protective immune responses against pathogens [28,29]. Herein, we used several in silico and immunoinformatics-based methods to predict immunodominant B-cell and T-cell epitopes from G1 and G2 of the Asia-1 Genotype of CCHFV and design a multi-epitope vaccine capable of protecting against the viral infection.

## 2. Material and Methods

### 2.1. Sequences Retrieval and Multiple Sequence Alignment

The M segment genome sequences available of Asia-1 genotype of CCHFV were accessed from NCBI, and corresponding glycoproteins sequences were downloaded with accession numbers AAM48107, AAK52742, BAB84572, AIE16134, ABB30033, AIE16135, ABB30035, ABB30032, AIE16129, AIE1612B8, AIE16130, AIE16131, AIE16133, AIE16132, AAW84284, AAK52743, ABB30029, BA84577, AF338470, and BAB84578. Multiple sequence alignment was performed with ClustalW implemented in BioEdit version 7.2 [30], using the multiple sequence alignment mode, keeping the gap open penalty of 10 and gap extension penalty of 0.05. Finally, from the sequence obtained after multiple sequence alignment, sequences of G1 and G2 were obtained and proceeded with epitope prediction studies.

### 2.2. Linear B-Cell Epitopes Prediction

The online tool integrated at Immune Epitope Database Analysis Resource (IEDB) (http://tools.immuneepitope.org/tools/bcell/iedb_input, accessed on 10 October 2022) was used to determine B cell linear epitope of G1 (644 amino acids) sequences using the method of Kolaskar and Tongaonkar (threshold value = 1) [31]. The method is based on the occurrence of amino acid residues in experimentally determined epitopes. The application of this method can predict antigenic determinants with 75% accuracy, which is better than most known methods [31].

### 2.3. Cytotoxic T-lymphocyte Epitopes Prediction

Cytotoxic T-lymphocyte [(CTL) also called CD8+ T-cell or major histocompatibility complex (MHC)-I binding epitopes)] epitope prediction was done using NetCTL-1.2 server (http://www.cbs.dtu.dk/services/NetCTL/, accessed on 10 October 2022) keeping a threshold score of >0.75000. HLA-A*02:01 supertype restricted CTL epitopes were predicted from the G1 sequence, while HLA-B*44:02 supertype restricted CTL epitopes were predicted from the G2 sequence.

### 2.4. Antigenicity, Allergenicity, Toxicity, and Non-Homology Analysis of Epitopes

The predicted epitopes were checked for antigenicity using the VexiJen v2.0 server (http://www.ddg-pharmfac.net/vaxijen/VaxiJen/VaxiJen.html, accessed on 10 October 2022), and epitopes that showed antigenicity value (model “virus”) ≥ 0.4 were selected for further analysis. Using the AllerTOP v.2.0 server (https://www.ddg-pharmfac.net/AllerTOP/, accessed on 11 October 2022), the antigenic epitopes were tested for the non-allergen character. Next, the epitope sequences were submitted to the ToxinPred server (http://crdd.osdd.net/raghava/toxinpred/, accessed on 12 October 2022) to filter toxic epitopes. Finally, to avoid an autoimmune reaction, the amino acid sequences of epitopes were checked for homology with the amino acid sequence of proteins included in the Homo sapiens proteome (NCBI Taxid:9606) via the BLASTp tool (https://blast.ncbi.nlm.nih.gov/Blast.cgi, accessed on 12 October 2022).

### 2.5. Construction of Multi-Epitope Chimeric Vaccine

Suitable linkers and an adjuvant were used to combine the selected epitopes in a rationally immunogenic manner. The explicit linkers, including EAAAK, GGGS, AAY, and GPGPG were employed to link the epitopes. The N-terminal of the construct started with an adjuvant, 50S ribosomal protein L7/L12 (UniProt entry: P9WHE3), to enhance the construct’s immunogenicity. Next, with the help of the EAAAK linker, the Pan DR epitope (PADRE—AKFVAAWTLKAAA) was added to perform a helper T-lymphocyte (HTL) stimulus role. Then, CTL epitopes were added and fused via the AAY linker, followed by the addition of B-cell epitopes using the GPGPG linker. The classes of epitopes were separated using the GGGS linker.

### 2.6. Population Coverage Analysis

The target population’s coverage of the chosen epitopes was estimated using the IEDB’s population coverage tool (http://tools.iedb.org/population/, accessed on 13 October 2022). We focused on population coverage of HLA binding alleles of selected peptides in Pakistan, Asia, and across the globe.

### 2.7. Evaluation of Immunological and Physicochemical Properties of the Chimeric Vaccine

The antigenic character of the constructed vaccine was predicted employing the VexiJen v2.0 (antigenicity score cut-off ≥ 0.4, model “virus”) and ANTIGENpro (https://scratch.proteomics.ics.uci.edu/, accessed on 13 October 2022) server. In order to evaluate whether the designed vaccine construct is labelled as allergen or non-allergen, two servers were used: AllerTOP v.2.0 and AllergenFP v.1.0 (https://ddg-pharmfac.net/AllergenFP/, accessed on 14 October 2022). In addition, the number of transmembrane (TM) helices in the constructed vaccine were predicted by deploying the TMHMM v2.0 tool (https://services.healthtech.dtu.dk/service.php?TMHMM-2.0, accessed on 14 October 2022). Furthermore, a set of physicochemical properties were estimated for the designed chimeric construct via the ProtParam server (https://web.expasy.org/protparam/, accessed on 14 October 2022). Additionally, using the SOLpro (https://scratch.proteomics.ics.uci.edu/, accessed on 14 October 2022) and Protein-Sol server (https://protein-sol.manchester.ac.uk/, accessed on 14 October 2022), the solubility of the constructed vaccine was assessed. Finally, the amino acid sequence of the designed vaccine construct was checked for any sequence homology with the proteins included in the Homo sapiens proteome by using the BLASTp tool.

### 2.8. Computational Immune Assay for the Chimeric Vaccine

In order to characterize the immunogenicity and immune response induced by the designed multi-epitope vaccine, a computational immune simulation was carried out employing the C-ImmSim server (https://150.146.2.1/C-IMMSIM/index.php, accessed on 17 October 2022). Considering the literature [32], three in silico vaccine doses were injected at different intervals of 4 weeks. The time steps of injections were 1, 84, and 170, involving a total of 1050 simulation steps, where a single time step is equal to 8 h of everyday life. The default setup was applied for all other simulation parameters.

### 2.9. Prediction, Refinement and Validation of Tertiary Structure of the Constructed Vaccine

Using the I-TASSER server (https://zhanggroup.org/I-TASSER/, accessed on 17 October 2022), the tertiary structure of the chimeric vaccine was predicted. Then, the modelled structure was submitted to the GalaxyRefine server (http://galaxy.seoklab.org/, accessed on 17 October 2022) for structural improvements. This server implements ab initio modelling in order to improve loops or terminal regions in the predicted three-dimensional (3D) structural model. Next, the accuracy of the optimized tertiary model of chimeric protein was validated by examining the PROCHECK [33] generated Ramachandran plot (that showed favourable backbone dihedral angles in relation to amino acid residues in tertiary structure) and ProSA-web (https://prosa.services.came.sbg.ac.at/prosa.php/, accessed on 17 October 2022) generated Z-score (that can be used to evaluate the overall and local quality of tertiary structure).

### 2.10. Molecular Docking Analysis

#### 2.10.1. Peptide Modelling and Docking Analysis of Predicted Epitopes with MHC-I

The selected epitopes were modelled using the PEP-FOLD3 server (https://bioserv.rpbs.univ-paris-diderot.fr/services/PEP-FOLD3/, accessed on 20 October 2022) with 2000 simulations and sOPEP sorting scheme. Human MHC-I allele HLA-A*02:01 (PDB ID: 5HHP) [34] and HLA-B*44:02 (PDB ID: 3L3D) [35] and three mouse MHC-I molecules ((H2-Db (classical, PDB ID: 1YN6), H2-Kb (classical, PDB ID: 6JQ3), and PDB ID: H2-M3 (non-classical) PDB ID: 7LFK)) were retrieved in PDB format from the RCSB Protein Data Bank (https://www.rcsb.org/, accessed on 20 October 2022). The protein binding motif residues were defined for HLA molecules as obtained from MHC Motif Viewer. The missing residues in retrieved HLA molecules were modelled using the Molecular Operating Environment version 2022.02 (MOE) [36]. Before proceeding to the protein–protein docking, a quick minimization of the protein was performed until an RMS gradient of 0.1 kcal/mol using the Amber14:EHT forcefield. The missing hydrogens and forcefield parameters were added using the MOE QuickPrep tool. The docking protocol was validated by redocking the HLA-A*02:01 and HLA-B*44:02 co-crystalized peptides. For redocking, the initial 100 pose placement of the peptide was done by coarse-grained (CG) model, and exhaustive sampling was carried out to generate a set of initial poses. The Hopf fibration method was used to generate a set of uniformly distributed rotations, and a Fast Fourier Transform (FFT) was used to sample all translations for a given rotation. The final 10 poses were selected based on the solvation free energy using the GBVI algorithm. The RMSD between the co-crystallized and docked peptides was determined. For each peptide, 10 docked poses were saved using the redocking protocols, and the optimum docked orientation of each peptide was determined based on the docking score (DS) and maximum binding interactions.

#### 2.10.2. Molecular Docking of Multi-Epitope Vaccine Construct and TLRs

In the present study, the binding affinity of the modelled vaccine for the Toll-like receptors (TLRs) was assessed by performing molecular docking. Employing the protein–protein docking protocol of MOE2022.02 software, the chimeric vaccine was docked with human Toll-like receptors TLR2, TLR3, and TLR4 with PDB IDs 2Z7X, 2A0Z, and 3FXI, respectively. Before executing docking, the retrieved crystal structures were edited to remove the attached oligosaccharides, ligands, and chains B, C, and D (only for TLR4), and were energy minimized with the Quick Prep module of MOE2022.02. The final 30 docked confirmations were retained, implementing the MOE2022.02′s rigid body refinement approach. The interface contact analysis of vaccine construct and immune receptor was done through the PDBsum server (http://www.ebi.ac.uk/thornton-srv/databases/cgibin/pdbsum/GetPage.pl?pdbcode=index.html, accessed on 22 October 2022), and structural illustrations were generated using the Blender software [37].

### 2.11. Molecular Dynamics Simulations

With molecular dynamic (MD) simulations, the dynamic behaviour of the modelled vaccine–TLRs complexes was examined. The TLR2 (PDB ID: 2Z7X), TLR3 (PDB ID: 2A0Z), and TLR4 (PDB ID: 3FXI) docked with the modelled vaccine construct and peptides docked with the HLA-B*44:02 (PDB ID: 3L3D) and HLA-A*02:01 (PDB ID: 5HHP) alleles were subjected to molecular dynamic (M.D.) simulations using the AMBER22 [38]. While generating the protein coordinates and topology with the LEaP module of AMBER22, the forcefield utilized for the protein is ff19SB [39] (amino acid-specific forcefield). During the simulation run, the monovalent OPC ions Na+ and Cl- (~0.1 M) were utilized at 1 Å grid to neutralize each system. The AMBER22 LEaP module was used to add the missing hydrogen from each system. The OPC (optimal point charge) water model was utilized with an octahedral box of 10 Å buffer distance to solvate the protein in each system while simulating. The long-range electrostatics were computed using AMBER22’s Particle Mesh Ewald Molecular Dynamics (PMEMD) [40] approach to enhance parallel scalability. For each system, a two-step minimization was carried out. Before doing the 5000 steps of conjugate gradients minimization, the 10,000 steps of the steepest descent algorithm minimization were completed [41]. Using weak constraints from the protein residues, the Langevin thermostat [42] was applied to steadily heat each system from 0.1 to 300 K in 400 ps. The Langevin thermostat and the collision frequency of 2.0 ps^−1^ were used to modify the protein kinetic energy of harmonic oscillators for dynamic propagation. At 400 ps, the protein density was modified following the heating procedures. Prior to production, equilibration was carried out utilizing the 300 K temperature and NPT ensemble for 2000 ps. The equilibration pressure relaxation time was chosen to be 2 ps. The hydrogen atoms were subjected to the shaking algorithm with a 2 fs time step [43]. The systems were subjected to a cut-off of 8 Å to compute the long-range electrostatics. The production runs of 100 ns for vaccine construct–TLRs complex systems and 50 ns for the peptide–HLA allele complexes were carried out using the equilibration methods. Each system’s output trajectory was written at a simulation time of 10 ps.

### 2.12. Analysis of MD Trajectories

The stability of each system’s 100 ns long output trajectory of the vaccine construct–TLR complex and 50 ns peptides–alleles complexes were examined using the CPPTRAJ module [44] of AMBER22. Each system’s 11,000 and 5000 frame trajectories were used to construct the Cα-atom-based Root Mean Square Deviation (RMSD). The starting frame of the trajectory was used as a reference coordinate for computing the RMSD. The average RMSD against time was obtained to comprehend the fluctuation in the RMSD of each system. The Root Mean Square Fluctuations (RMSF), Radius of Gyration (Rg), Solvent Accessible Surface Area (SASA), and native contacts analysis were performed for the vaccine construct–TLRs complex systems utilizing the CPPTRAJ of AMBER22.

### 2.13. Binding Free Energy Calculation

The Molecular Mechanics/Generalized Born Surface Area (MM/GBSA) approach was used to determine the binding free energies of the vaccine construct–TLRs and peptides– HLA alleles complexes [45,46,47]. Using the 2 Å solvent probe and the mbondi3 radii, the topology of each system was tuned. Following Equation (1), the binding free energy (ΔGbind) was computed. Next, the protein-ligand complex energy (ΔGR + L) was subtracted from the receptor (ΔGR) and ligand (ΔGL) individual energies to compute the binding free energy. The binding free energies of the modelled vaccine and peptides were determined using the final 2200 frames for vaccine construct–TLRs complexes and the final 1000 frames for peptide–HLA allele complexes (20 ns) of each trajectory using Equation (1).

(1) $${\Delta G}_{\mathrm{bind}}={\;\Delta G}_{R+L}-\left({\Delta G}_{R}+{\Delta G}_{L}\right)$$

Following Equation (2), the individual energies that made up each system energy in Equation (1) were calculated.

(2) $$G=E_{BOND}+E_{VDW}+E_{ELEC}+G_{PB}+G_{SA}-T_{SS}$$

Dihedrals and angels (G_BOND_), van der Waals energies (G_VDW_), electrostatic energies (G_ELEC_), polar and non-polar solvation energies (G_PB_ and G_SA_), and temperature (T) with the solute entropy (SS) are the contribution energies of each system in Equation (2). Except for the polar solvation energy, computed using the LCPO technique, all other energies were calculated in kcal/mol, and the protein surface area was estimated in Å^2^. Finally, the total energy of each system was estimated by adding the given electrostatic energies.

## 3. Results

### 3.1. Sequence Retrieval and Conservation

The Congo virus Asia-1 genotype glycoproteins sequences were retrieved from NCBI with accession numbers AAM48107, AAK52742, BAB84572, AIE16134, ABB30033, AIE16135, ABB30035, ABB30032, AIE16129, AIE1612B8, AIE16130, AIE16131, AIE16133, AIE16132, AAW84284, AAK52743, ABB30029, BA84577, AF338470, and BAB84578. The ClustalW alignment shows 87–99% genome similarity of the Congo virus Asia-1 genotypes (Supplementary File S2A). The G1 protein alignment shows a high conservation of 89–100% between the genome sequences (Supplementary File S2B). Similarly, the G2 protein conservation was reported from 95–100% between the genome’s sequences (Supplementary File S2C). Based on the high conservation of G1 and G2, these protein sequences were selected for further analysis.

### 3.2. Linear B-Cell Epitopes Prediction

A total of 12 B-cell epitopes were predicted for G1 and G2 proteins. Three non-antigen and four allergen epitopes were filtered; the finalized five probable antigen and non-allergen epitopes had antigenicity scores ranging from 0.42 to 1.37. ToxinPred server labelled these epitopes as non-toxic, and they did not show an exact match with the human protein sequences, thus classifying them as safe from triggering an autoimmune response (Table 1).

### 3.3. CTL Epitope Prediction

A total of 18 CTL epitopes were predicted for G1 and G2 proteins, indicating a high affinity for the respective HLA supertype allele. We further shortlisted six epitopes (five for G1 protein and a single epitope for G2 protein) based on probable antigenicity, non-allergenicity, non-toxicity, and non-homology. VexiJen v2.0 server predicted antigenicity scores of finalized CTL epitopes varied from 0.65 to 1.54. The selected six CTL epitopes and their several predicted parameters are provided in Table 2.

### 3.4. Construction of Multi-Epitope Vaccine Construct

**Figure 1:**
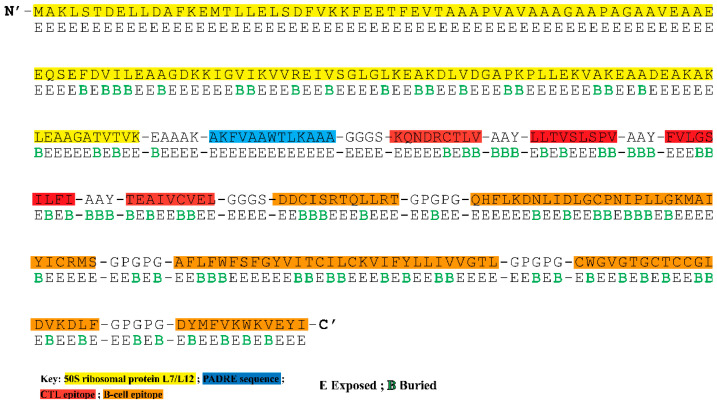
The amino acid sequence and surface accessibility of the constructed highlighted N-terminal has an attached adjuvant (50S ribosomal protein L7/L12) highlighted in yellow. The PADRE sequence is represented in dark blue colour. CTL, and B-cell epitopes are highlighted in red and orange. Surface accessibility (NetSurfP—3.0 sever) of each amino acid is shown as either exposed (E) or buried (B) residue.

The final potential epitopes were fused using suitable linkers and an adjuvant to construct a multi-epitope vaccine. The final 324 amino acid long sequence of the designed vaccine construct is shown in Figure 1. An adjuvant, derived from *Mycobacterium tuberculosis* (strain ATCC 25618/H37Rv) was appended at the N-terminal, followed by the EAAAK linker, the PADRE epitope (13aa), and GGGS linker. Four unique CTL epitopes were linked together using the AAY linker. A GGGS linker was added between the last CTL epitope and the first B-cell epitope. The connection of five B-cell epitopes was assisted by adding the GPGPG linker. The B-cell epitope “AFLFWFSFGYVITCILCKVIFYLLIVVGTL” is an overlapping peptide containing amino acid sequences of two selected CTL epitopes (FLFWFSFGY and YLLIVVGTL)**.**

### 3.5. Analysis of Surface Accessibility and Population Coverage of the Chimeric Construct

The solvent accessibility of each residue in the multi-epitope vaccination ensemble was returned by the NetSurfP-3.0 server (Figure 1). In the B-cell epitopes, this position (exposed (E)) was best defined for 66.94% of the residues, indicating their suitability for antibody or B-cell receptor recognition. For CD8+ T-cell epitopes, 62.16% exposed and 38.84% buried (B) residues are estimated. The population coverage analysis based on CTL epitopes showed that the constructed vaccine is expected to cover 60.52% of the global, 69.85% of Pakistani, 45.54% of Southwest Asian, 35.99% of East Asian, 31.98% of South Asian, 30.33% of Northeast Asian, and 29.51% of Southeast Asian population (Table S1).

### 3.6. The Chimeric Vaccine Accumulates Features of Safety and Effective Antigen

A search for sequence similarity between proteins from *Homo sapiens* and the multi-epitope vaccine showed no significant matches. When submitted individually to a BLASTp analysis, the chimeric epitopes also showed non-homology with the proteins of the human proteome. Hence, the final components of the multi-epitope vaccine construct were kept unchanged. As per the AllergenFP v.1.0 and AllergenTOP v.2.0 results, the proposed vaccine construct is expected to be non-allergenic. These findings suggest that the hypothetical antigen might possess safety in in vivo assays. The chimeric protein was classified as a potential antigen after the vaccine’s antigenicity tests revealed scores of 0.51 (VexiJen v2.0) and 0.84 (ANTIGENpro).

**Figure 2:**
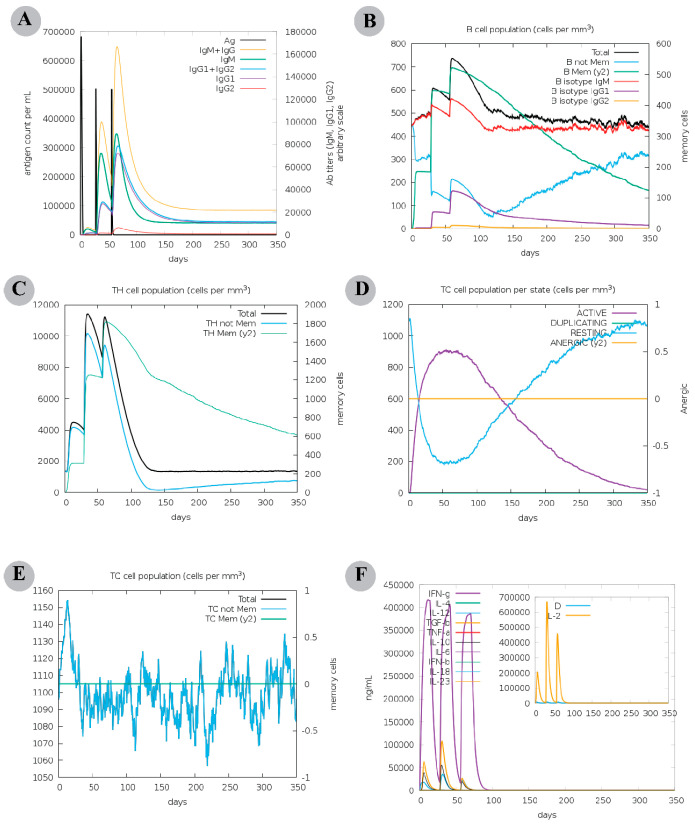
Computation immune simulation of an infection challenge that included Congo virus proteins sequences covered by the designed chimeric vaccine was simulated for 350 days. (**A**) Antibodies response against the antigen (or virus). Following the antigen injection, antibodies and immunocomplexes indicate the induction of humoral immune response with a change toward a variety of antibodies subtypes (with IgG subtype being the predominant one). (**B**) Population of B lymphocytes in a cell per mm^3^ in terms of the total count, generation of memory cells, and induction of several isotypes, including IgM, IgG1, and IgG2. (**C**) Concentration of total and memory CD4+ T-helper (TH) lymphocytes. (**D**) Concentration of CD8+ T-cytotoxic (TC) lymphocytes in several forms, i.e., active, duplicating (in the mitotic cycle), resting (not active), and anergic. (**E**) The cell count of total and memory CD8+ T-cytotoxic (TC) lymphocytes. (**F**) Levels of cytokines and interleukins. D in the inset plot is a danger signal and leukocyte growth factor IL-2.

The secondary and tertiary immune responses exceeded the primary immune response in an in silico immunological simulation, demonstrating behaviour comparable with the results of actual vaccination. Elevated antibody titre per ml of IgM + IgG (>160,000), IgM (>80,000), IgG1 + IgG2 (>60,000), and IgG1 (>60,000) was detected, followed by a decrease in antigen concentration (Figure 2A). A substantial count of total B cells (>450 cells per mm^3^) (Figure S1A), memory B-cell population (nearly 450 cells per mm^3^) (Figure 2B), and plasma B-cell population were detected (Figure S1B). A steady count of memory T helper (TH) cells (peak value > 1600 cells per mm^3^) was maintained (Figure 2C) and many active TH cells lasted unitl the end (Figure S1C). Higher count of active T cytotoxic cells (TC) (Figure 2D) and memory TC cells suggests the development of a long-lasting cellular response (Figure 2E). Also, the results revealed a decline in active regulatory T-cells (TR) after reaching a maximum count of ~1400 cells per mm^3^ (Figure S1D). Among the innate immune cells, the data demonstrated sustained levels of macrophages (>150 cells per mm^3^) and dendritic cells (DCs, >140 cells per mm^3^) (Figure S1E & F). Finally, an elevated levels of IFN-γ (>400,000 ng/mL) and transforming growth factor-b (>100,000 ng/mL) was seen, and a higher Simpson index suggested the production of various cytokines in response to multi-epitope vaccine antigen (Figure 2F). These findings propose the probability of a chimeric vaccine to stimulate a robust cellular and humoral immune response. Nevertheless, an experimental consideration is warranted to clarify the constructed vaccine’s potential to induce adaptive immunity against the Asia-1 genotype of CCHFV.

### 3.7. Physicochemical Analysis Suggested Positive Parameters for Vaccine Production

A set of physicochemical properties for the proposed vaccine construct were calculated using the ProtParam server and are shown in Table 3. The construct’s theoretical isoelectric point (pI) is 5.01 (acidic), and its estimated molecular weight is 33.88 kDa. There were 37 residues that were negatively charged and 30 that were positively charged. An estimated instability index of the multi-epitope vaccine is 24.69, classifying the protein as stable (a value between 0 and 40 indicates a stable protein). The protein’s stability across a wide temperature range was also strengthened by its high aliphatic index (102.75). The protein’s heterologous expression in bacteria or yeast depends on a long half-life time. The designed vaccine construct has an estimated half-life of 30 h in mammalian reticulocytes (in vitro), over 20 h and 10 h, respectively, in yeast and E. coli (both in vivo). Besides, the chimeric antigen is predicted to have no TM helices, underlining its suitability for future application.

### 3.8. The Modelled Vaccine Attained a Desirable 3D Orientation

**Figure 3:**
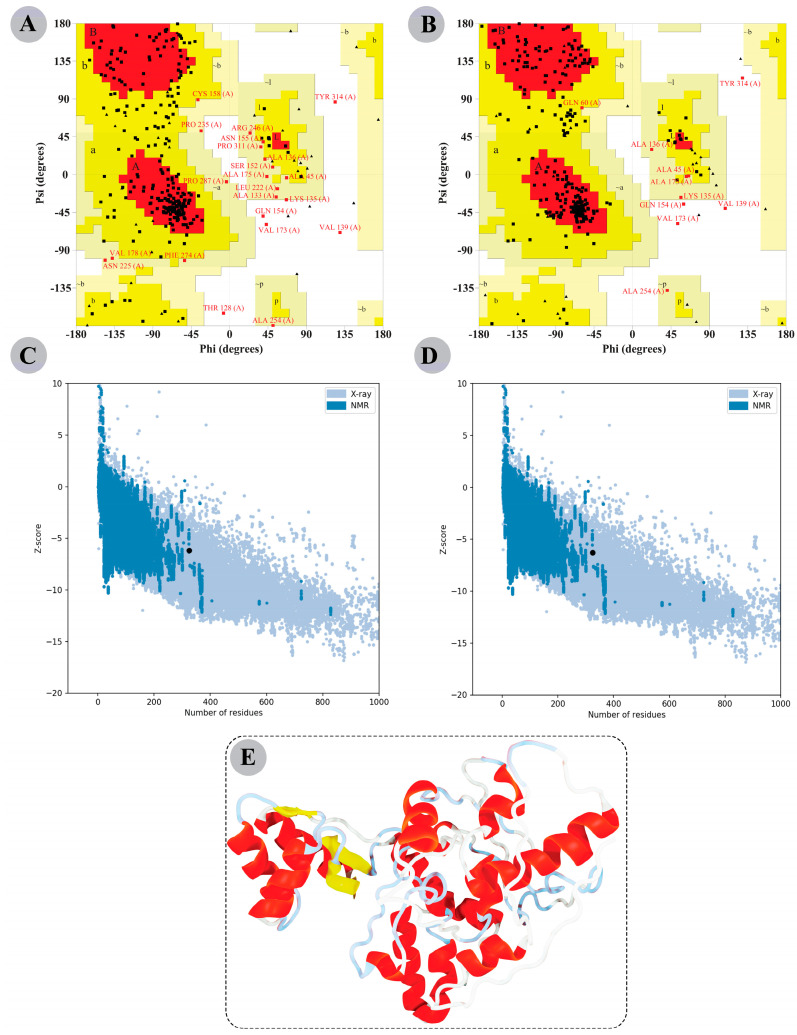
Tertiary structure modelling, refinement, and validation of the chimeric vaccine construct. Ramachandran plot details of modelled structure (**A**) before and (**B**) after refinement. ProSA web server generated a Z-score graph of the modelled structure (**C**) before and (**D**) after refinement, depicting that the modelled 3D structure is comparable to the X-Ray crystallographic determined structure for the protein of similar sizes. (**E**) Representation of 3D modelled final structure (after refinement).

I-TASSER server yielded five possible 3D structures for the constructed vaccine with C-scores ranging from −3.34 to −4.25. A model was deemed perfect if more residues were positioned in the Ramachandran plot’s favoured regions and fewer in the disallowed regions. Model 5 has the highest C-score of −4.25. The Ramachandran plot analysis revealed 64.3% residues in favoured regions, 28.7% in additional allowed regions, 5.1% in generously allowed regions, and 1.8% in disallowed regions for this modelled structure. Thus, Model 5 was selected for structural refinement via the GalaxyRefine server, which also yielded five models (Table S2). Compared to the other models, the improved Model 3 showed more residues collectively in favoured and allowed regions (98.5%) and only 1.5% in disallowed regions of the Ramachandran plot. Additionally, the ProSA-web estimated Z-score of the initial and refined model was −6.33 and −6.14, respectively. A lower Z-score means our improved final model is of high quality. Figure 3 shows the Ramachandran plot information and Z-score of the initial and refined designed vaccine 3D model. The 3D structure of the refined modelled chimeric construct is also illustrated in Figure 3.

### 3.9. Molecular Docking Analysis

#### 3.9.1. Peptide Modelling and Docking Analysis of Predicted Epitopes with MHC-I

**Figure 4:**
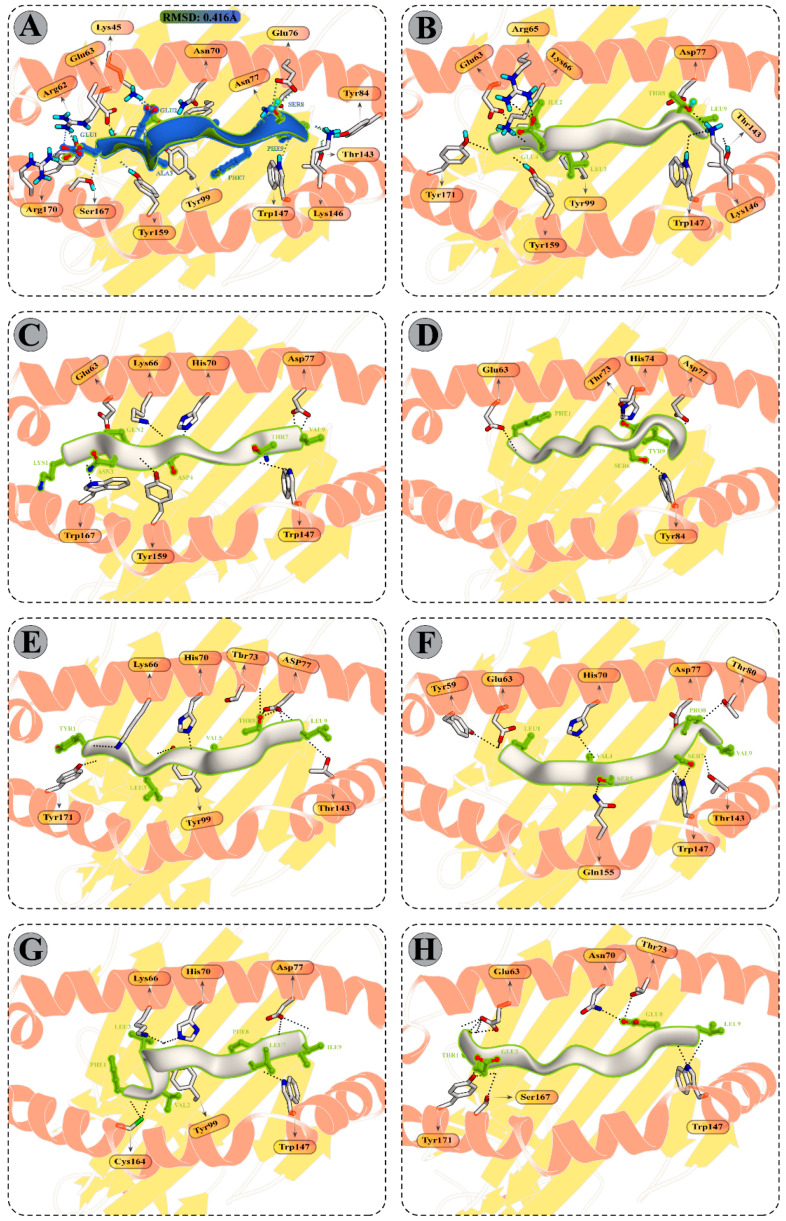
Molecular docking of CTL peptides and HLA class I molecules (**A**) Peptide EEAGRAFSF (Green) co-crystalized in the binding pocket of HLA-B*44:02 (PDB ID: 3L3D). The Blue peptide represent the re-docked peptide. (**B**) Peptide GILEFVFTL (PDB ID: 5HHP) co-crystallized in the binding pocket of HLA-A*02:01. (**C**) Peptide KQNDRCTLV attached with the binding pocket of HLA-A*02:01. (**D**) Peptide FLFWFSFGY attached with the binding pocket of HLA-A*02:01. (**E**) Peptide YLLIVVGTL attached with the binding pocket of HLA-A*02:01. (**F**) Peptide LLTVSLSPV attached with the binding pocket of HLA-A*02:01. (**G**) Peptide FVLGSILFI attached with the binding pocket of HLA-A*02:01. (**H**) Peptide TEAIVCVEL attached with the binding pocket of HLA-B*44:02. All H-bonds are represented in dotted lines and the interacting residues of HLA class II molecule is shown in stick.

Molecular docking between selected CD8+ T-cell peptides and specific HLA molecules showed a good DS (Table S3) and several molecular interactions, such as H-bonds and ionic interactions (Figure 4 and Table S4). The reference docked complexes HLA-B*44:02 (PDB ID: 3l3D) and HLA-A*02:01 (PDB ID 5HHP) indicated a DS of −18.89 kcal/mol and −40.53 kcal/mol and an RMSD of 0.416 Å and 0.900 Å respectively, confirming the best fit of a ligand. The reported DS of the selected peptides are −60.21 kcal/mol (KQNDRCTLV), −56.85 kcal/mol (FLFWFSFGY), −37.35 kcal/mol (YLLIVVGTL), −53.81 kcal/mol (LLTVSLSPV), and −55.69 kcal/mol (FVLGSILFI), with the HLA-A*02:01 receptor, while the DS of the TEAIVCVEL peptide with the HLA-B*44:02 receptor calculated is −37.83 kcal/mol, indicating that peptides were docked well within the binding groove of HLA receptor. The chosen CTL peptide also revealed a striking docking affinity and several intermolecular contacts with the mouse MHC-I alleles (Table S5). With classical MHC-I alleles, including H2-Db and H2-Kb, the DS of selected peptides varies from −35.10 to −51.47 kcal/mol and −34.10 to −44.10 kcal/mol, respectively. In contrast, the finalized CTL epitopes indicated docking energy ranging from −31.13 kcal/mol to −52.97 kcal/mol with H2-M3 (non-classical MHC-I allele3).

#### 3.9.2. Molecular Docking of Modelled Vaccine with TLRs

Interaction between antigen molecules (putative vaccine construct) with appropriate immune receptors would be crucial for the cellular transport of antigen molecules and activation of the downstream immune pathway. The predominant binding mode of the vaccine construct–TLR2/3/4 had striking DS of −80.44 kcal/mol, −99.37 kcal/mol, and −79.29 kcal/mol, respectively, suggesting the strong binding of the chimeric antigen with the receptor binding pocket.

**Figure 5:**
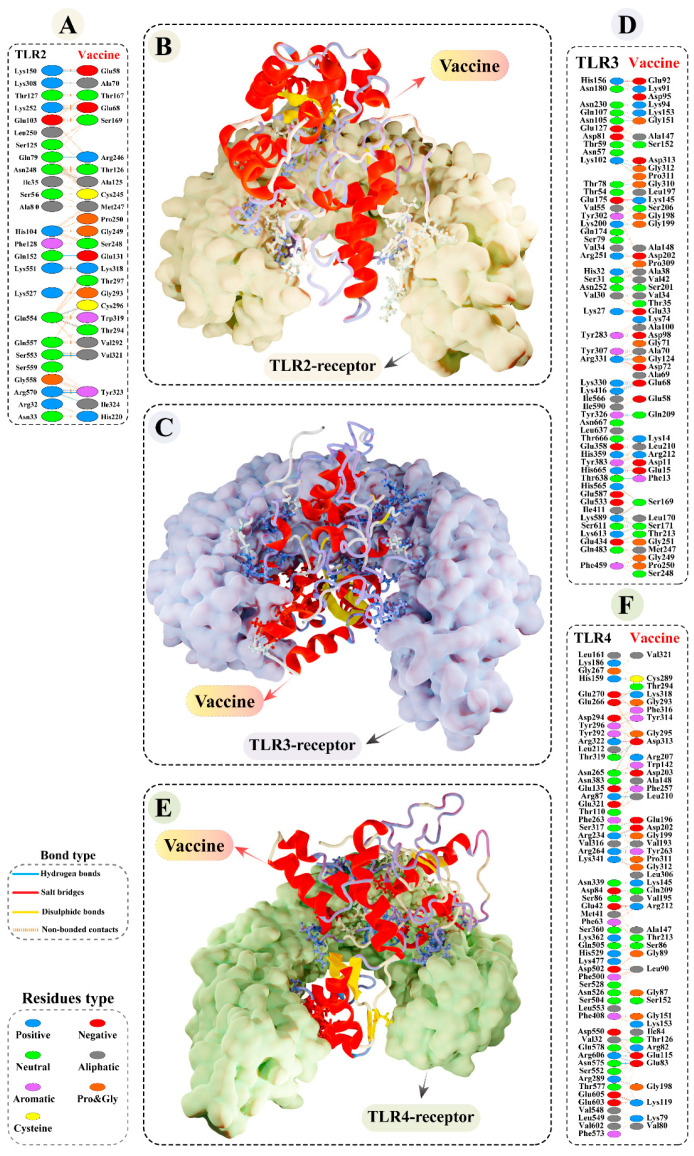
Molecular docking study of the modelled vaccine construct and Toll-like receptor (TLR2, TLR3, and TLR4). (**A**) Interface contacts between the putative vaccine construct and TLR2. Best binding confirmations of (**B**) modelled vaccine construct–TLR2 complex and (**C**) modelled vaccine construct–TLR3 complex. (**D**) Interface contacts between the modelled vaccine construct and TLR3. Best binding confirmation of (**E**) modelled vaccine construct–TLR4 complex. (**F**) Interface contacts between the modelled vaccine construct and TLR4. Hydrogen bonds are depicted in blue lines. The residue type is shown with a distinctive colour.

Interface contacts between vaccine construct and immune receptors revealed multiple interactions, such as H-bond, salt bridges, and non-bonded contacts. In case of vaccine construct–TLR2 complex, a total of 18 H-bonds was formed between the modelled vaccine residues and TLR2 with a bond distance ranging from 2.73 to 3.18Å (Figure 5A,B, and Table S6). In addition, GLU58 and GLU68 denoted salt-bridges interaction with this receptor, having a bond distance of 2.73Å and 3.97Å, respectively. There were 36 H-bonds (bond distance range: 2.58 to 3.09Å) and eight slat-bridges interactions (bond distance range: 2.66Å to 3.84Å) observed between the vaccine construct and TLR3 interface (Figure 5C,D and Table S7). Similarly, there were 26 residues from the modelled vaccine side that mediated hydrogen bonding within a 3.29Å bond distance (Figure 5E,F and Table S8) and six residues showed salt-bridge interactions within 2.84 Å bond distance with the TLR4.

### 3.10. Molecular Dynamics Simulations of CTL Epitopes–HLA Complex

The attachment stability of the selected peptides (KQNDRCTLV, FLFWFSFGY, YLLIVVGTL, LLTVSLSPV, FVLGSILFI, and TEAIVCVEL) with the HLA-B*44:02 (PDB ID: 3l3D), and HLA-A*02:01 (PDB ID 5HHP) during the simulation run was confirmed via RMSDs calculation base on the Cα atoms of the protein. The HLA-A*02:01 and HLA-B*44:02 receptors bind with the co-crystalized peptides and were selected as reference systems in the MD. The RMSD of each system is depicted in Figure S2. The 3L3D system RMSD shows stability until 35 ns, while an increase was observed until the end of the simulation. The 5HHP, KQNDRCTLV, FLFWFSFGY, LLTVSLSPV, and TEAIVCVEL peptides show stable behaviour after 35 ns of simulation time, where the systems converged. The YLLIVVGTL, and FVLGSILFI systems stabilize the RMSD after 40ns simulation time until the end. The RMSD analysis of the peptide complexes shows no sudden fluctuation in the values, which signifies the results.

### 3.11. Molecular Dynamics Simulations of Modelled Vaccine–TLRs Complex

**Figure 6:**
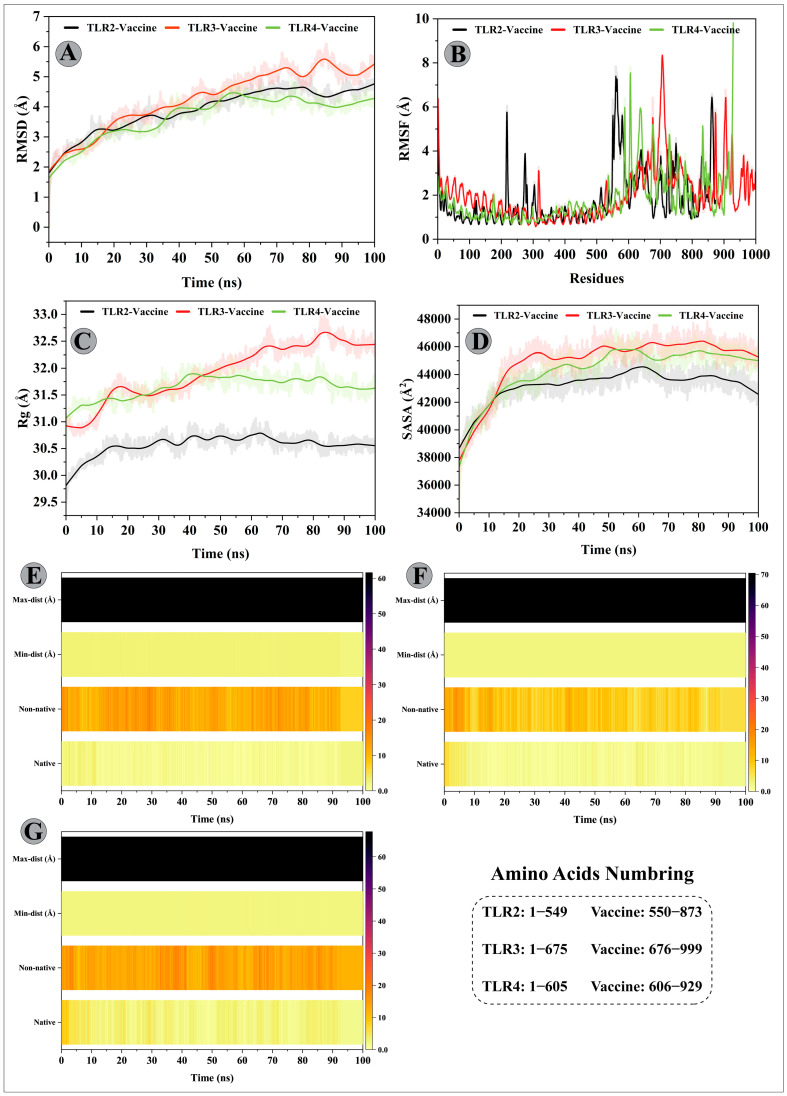
Molecular dynamics simulations analysis of the designed vaccine construct (ligand) and immune receptor (TLR2/3/4) complexes. (**A**) Root Mean Square Deviation (RMSD) plots of ligand and receptor complexes show no substantial deviations from their reference starting position and establishment of a stable complex. (**B**) Root Mean Square Fluctuation (RMSF) plots of ligand and receptor complexes demonstrating the flexibility of amino acid side chains. (**C**) The structural compactness of ligand–receptor complexes estimated over 100 ns simulation time is represented by the Radius of Gyration (Rg) plots. (**D**) Changes in the protein surface area accessible to the solvent over 100 ns simulation time represented by Solvent Accessible Surface Area (SASA) plots of ligand–receptor complexes. Native and non-native contacts between (**E**) Chimeric vaccine construct–TLR2 complex, (**F**) Chimeric vaccine construct–TLR3 complex, and (**G**) Chimeric vaccine construct–TLR4 complex estimated over the 100 ns simulation timescale.

To characterize the confirmational stability of the vaccine construct and TLRs complexes, the RMSDs were calculated and produced as a graph based on the Cα atoms of the protein (Figure 6A). The mean RMSDs computed for the vaccine construct–TLR2/3/4 complexes were 3.90 Å, 4.26 Å, and 3.68 Å, respectively. An upward trend was observed for the chimeric construct–TRL2 complex until 70 ns, whereby an RMSD value steadily increased from 1.80 Å to 4.80 Å. Then, the RMSD value remained stable until the end of the simulation time. A similar increasing RMSD pattern can be seen for the chimeric construct–TLR3 complex; however, the recorded values were highest in the period of 0ns to 70 ns among the simulation complexes (minimum and maximum fluctuation range 1.85 Å and 5.28 Å), indicating significant structural alterations in the receptor after the modelled vaccine attachment. This complex system also underwent structural deviations from 80 ns to 90 ns with a peak RMSD reaching 5.82 Å and achieving equilibrium towards the end. In case of the modelled vaccine–TLR4 complex, a stable protein–protein complex was formed following the 55 ns of simulation time. Before that, a steadily improved RMSD value was detected (minimum and maximum fluctuation range 1.62 Å and 4.46 Å) with slight deviations.

To gain insight into protein mobility at the amino acids level, the RMSF of the modelled vaccine–TLRs complexes was evaluated (Figure 6B). The average RMSF values of 1.78 Å, 2.01 Å, and 1.76 Å were estimated for the designed vaccine construct–TLR2/3/4 complexes, respectively. In all complexes, the N-terminal residues exhibited higher mobility, and a few residues of the C-terminal in the modelled vaccine–TLR4 complex also fluctuated to a higher degree. In all complexes, the adjuvant residues of the constructed vaccine reflected higher flexibility. For instance, the adjuvant residues belonging to segments MET–THR35, GLY199–SER201, and PRO309–LYS318 showed fluctuations > 4 Å in the vaccine construct–TLR2 complex. Besides, the amino acid segment of TLR2 comprising LEU216–GLY219 was more mobile (RMSF > 4 Å) than the rest of the residues. In the case of the vaccine construct–TLR3 complex, apart from adjuvant residues (LEU18–ALA45; RMSF range = 4.09 Å to 8.35 Å), LEU197–LEU200 (RMSF > 4.5 Å), ASN225–ILE234 (RMSF range 4.02 Å to 6.42 Å) regions of the designed vaccine construct (B-cell epitopes residues) exhibited higher fluctuations. TLR3 residues, however, remained stabilized after the designed vaccine construct. Similarly, the adjuvant residues, especially MET1–LYS3 (RMSF range 4.81 Å to 7.82 Å) and VAL25–LYS28 (RMSF range 4.04 Å to 4.78 Å) indicated higher fluctuations; also, the B-cell epitope residues ASP228-GLY230 showed greater flexibility (RMSF > 4 Å). In addition, a few residues from the TLR4 side displayed RMSF > 5.2 Å. Moreover, most of the designed vaccine construct residues that mediated H-bond interactions with the TLRs (mentioned in the docking results) showed a stable RMSF value > 3 Å.

The higher mobility shown by residues comprising adjuvant and B-cell epitopes could be crucial for the modelled vaccine to attain a favourable confirmation in order to bind the immune cells. To examine the structural compactness and tight packing of secondary structure elements (alpha helices and beta-sheets) in the tertiary structures of vaccine construct–TLRs complexes, the Rg of each complex was calculated and plotted as a graph (Figure 6C). Consistent with the RMSD results, vaccine construct–TLR2/3 complexes indicated tight packing of secondary structure elements throughout the simulation time, with Rg values less than 30.70 Å and 32 Å detected for these complexes, respectively. The modelled vaccine–TLR3 complex, on the other hand, reflected a steady rise in Rg value in the period of 10–18 ns and 25–60 ns, with a gain in the value 0.8 Å and 1.4 Å, respectively. The system attained an equilibrium towards the end. To investigate the surface area of protein exposed to the solvent molecules, the SASA values were calculated for each complex (Figure 6D). In case of vaccine construct–TLR2 complex, the initial 10 ns saw more and more internal residues exposure towards the solvent side (gain in SASA value 4000 Å^2^), followed by maintenance of a compact complex (SASA value ~43,235 Å^2^) until the end of the simulation. For the vaccine construct–TLR3 complex, the SASA value substantially increased from 37,778 Å^2^ to 45,570 Å^2^ during 0 ns to 25 ns period and sustained around the mean value of 44,935 Å^2^ with minor fluctuations. The SASA value of vaccine construct–TLR4 complex (mean value 44,315 Å) steadily increased until 60ns (from 37,322 Å^2^ to 45,750 Å^2^; the difference in SASA value was 8428 Å^2^), suggesting a structural change has increased the protein contact with the solvent. The remaining simulation time showed conformational stability for this complex.

The strength of intermolecular contacts between vaccine–TLRs interface was investigated by calculating the native contacts’ lifetime during the 100 ns simulation. In the vaccine construct–TLR2 complex, high-frequency H-bond was detected between VAL168_construct_ and GLU77_TLR2_ (2X; collective frequency of 61%), GLU322_construct_ and ARG544_TLR2_ (H-bond frequency 23%), ALA57 and LY124 (21%), and ALA69 and LYS282 (11%). There were additional H-bonds with retention frequency > 10% enlisted in Table S9.

In the case of vaccine construct–TLR3 complex, H-bonds between GLY151_construct_–ASN36_TLR3_, GLY310_construct_–LYS81_TLR3_, VAL168–GLU512, and SER201–PRO230, and PRO311–LYS81 maintained effectively for 37%, 24%, 22%, 20%, and 19% of simulation fraction, respectively. Besides, hydrogen bonding shown by designed vaccine residues LEU144, GLY312, LEU67, VAL168, and GLY151 with TLR3 residues GLU154, LYS81, LYS309, HIS544, and ASP60 was maintained for over 10% of simulation time. H-bond analysis details for the vaccine–TLR3 interface are provided in Table S9.

Similarly, for the designed vaccine construct–TLR4 complex, H-bonds formed between CYS194_construct_ and ASN313_TLR4_, LEU211_construct_ and GLU216_TLR4_ (2X), GLY312 and ARG296, ARG82 and ASN549, and VAL195 and SER291 were retained for 66%, 63%, 47%, ~44%, and ~29% of simulation fraction, respectively. Moreover, the modelled vaccine residues LEU311, VAL317, ALA318, and ARG82 mediated H-bond with LYS315, GLU244, GLU577, and ARG580 residues of TLR4, respectively, with over 10% frequency during the simulation. H-bond analysis details for the vaccine construct–TLR4 interface are provided in Table S9. In addition to the native contacts, non-native contacts between the chimeric construct and TLR2/3/4 interface were sustained throughout the simulation, indicating a stable molecule binding (Figure 6E–G).

### 3.12. Binding Free Energy Calculation of Selected Peptide–HLA Molecule Complexes

The binding free energy (∆_G TOTAL_) of the selected peptides (KQNDRCTLV, FLFWFSFGY, YLLIVVGTL, LLTVSLSPV, FVLGSILFI, and TEAIVCVEL) with the reference systems (3L3D and 5HHP) were calculated using the MMGBSA approach. The reference systems 3L3D and 5HHP binding free energy calculated with the peptides are −55.48 kcal/mol and −45.37 kcal/mol (Table 4). The highest binding free energy of −57.50 kcal/mol, −68.84 kcal/mol is reported for FLFWFSFGY and YLLIVVGTL systems compared to the reference systems. The TEAIVCVEL system binding energy (−55.21 kcal/mol) is higher than the reference system 3L3D. Similarly, in the KQNDRCTLV peptide system, the binding energy is greater than the reference system 5HHP. Furthermore, the LLTVSLSPV and FVLGSILFI systems’ binding energies −45.3 kcal/mol and −43.70 kcal/mol, respectively, are closely related to the reference system 5HHP. The binding energies of the docked peptides compared to the reference systems were superior, indicating stability and more energetically favourable binding energy. Electrostatic energy (∆_EEL_) and Van der Waals energy (**∆_VDW_**) were the dominant contributors to the total interaction energies of receptor–peptide complexes.

### 3.13. Binding Free Calculation of Vaccine–TLRs Complexes

The ∆G _TOTAL_ computed via the MMGBSA method for the vaccine construct–TLR2/3/4 complexes were −80.79 kcal/mol, −79.43 kcal/mol, and −84.24 kcal/mol, respectively (Table 5). The negative **∆_G TOTAL_** indicates stability and energetically favourable binding of complexes within the biological system. Analysing the free energy components revealed that ∆_EEL_ and **∆_VDW_** contributed most towards the overall interaction energies of vaccine construct–TLR2 and vaccine construct–TLR3 complexes. On the other hand, the polar component of solvation energy (∆_EGB_) and ∆_VDW_ energy made the highest contribution to the total free energy of vaccine–TLR4 complex.

## 4. Discussion

Epitope-based vaccine designing has gained considerable attention owing to its promising features over traditional vaccination. Unlike the single epitope or conventional vaccine, the multi-epitope vaccine benefits from including several MHC-restricted epitopes that T-cell receptors can recognize from various T-cell subsets. They can simultaneously elicit humoral and cellular immune responses due to the inclusion of CTL, HTL, and B-cell epitopes. Besides, the multi-epitope vaccine contains adjuvant substances that can enhance immunogenicity and minimize the use of undesirable substances which can otherwise cause abnormal immunological reactions [23]. Previously, several immunoinformatics-guided studies have designed the multi-epitope vaccine against pathogenic viruses, such as Cytomegalovirus, Dengue, Ebola, Hepatitis C, MERS-CoV, SARS-CoV-2, Zika, etc. [48,49,50]. Experimental approaches have validated the efficacy of in silico constructed multi-epitope vaccine against Mycobacterium tuberculosis [51] and SARS-CoV-2 [52]. Also, multi-epitope vaccine designed using a similar strategy has been shown to exhibit protective effects in vivo [53,54], and several of these vaccines have progressed to the clinical trial stage [55,56,57]. Considering the significant health burden associated with CCHFV (Asia-1 genotype), designing a multi-epitope vaccine against the virus could be desirable. The current study aimed to formulate a multi-epitope vaccine using the immunoinformatics-guided approaches capable of eliciting a robust immune response against the CCHFV (Asia-1 genotype) infection in humans.

Typically, pathogens’ surface proteins are more likely to interact with the host’s immune system and trigger an immunological response [58]. Here, we screened the surface glycoproteins (G1 and G2) of CCHFV to predict the immunodominant B-cell and CTL epitopes. This feature is crucial for the vaccine design, as B-cells are associated with antibodies production and CTLs show a major cytotoxic activity against cells infected with intracellular microbes [59]. Besides, the HTL epitopes can be mapped from the structural glycoproteins of CCHFV (Asia-1 genotype) for novel multi-epitope-based vaccine designing. Antigenicity prediction indicated the immunogenic potential of epitope as probable antigen and non-allergenic character labelled them safe from causing harmful allergen reactions in humans. The selected CTL epitopes showed high affinity for HLA-A*02:01 and HLA-B*44:02 alleles, which are reported to have an overall average frequency of 15.28% and 21.62%, respectively [60] around the world. Linking the finalized six CTL and five B-cell epitopes and fusing with other chimeric vaccine components were done using multiple linkers. The main advantage of using linkers in a multi-epitope vaccine is that they avert the formation of junctional immunity and assist in the processing and presentation of antigens [61]. Following the literature [52,62,63,64], several linkers (EAAAK, GGGS, AAY, and GPGPG) were used to construct the chimeric vaccine. CTL and B-cell epitopes were connected using the AAY and GPGPG linker, as reported by Robert et al. [65]. Besides acting as a proteasomal cleavage site, the AAY linker supports the epitope presentation by helping them find a suitable site for attachment on TAP transporters [66,67]. The GPGPG linker averts the formation of junctional immunity and promotes HTLs immune response, as demonstrated experimentally by Livingstone et al. [68] in mouse models. EAAAK linker was utilized to provide structural stiffness that can lessen the interference from other protein regions during the interaction between the adjuvant and its receptor [69]. In order to provide flexibility in the protein 3D structure, an alternative linker, GGGGS, was used [70]. Subunit vaccines are often less immunogenic and effective, requiring the inclusion of an adjuvant, which can promote and direct the adaptive immune response to the antigen (vaccine construct) [71]. Thus, we used 50S ribosomal protein L7/L12 to formulate a multi-epitope vaccine construct. Upon activation of naïve T-cells, the 50S ribosomal protein L7/L12 is reported to induce the maturation of DCs, CTLs, HTLs, and IFN-γ-producing cells [72].

The refined modelled tertiary structure of the putative vaccine construct indicated a Z-score of −6.14, which corresponds to the X-crystallography-determined structures for proteins of similar sizes. Moreover, the predicted modelled vaccine structure in this study is comparable to the study of Droppa-Almeida et al. [73] and Rekik et al. [74], wherein the predicted tertiary structure of the constructed vaccines had a Z-score of −5.26 kcal/mol, and −9.51 kcal/mol, respectively. Therefore, the predicted 3D structure of the multi-epitope vaccine construct was deemed a high-quality one and was used for the downstream structure-based analysis.

TLRs are Pattern Recognition Receptors (PRRs), which are expressed on both innate immune (DCs and macrophages) and non-immune cells (fibroblast cells and epithelial cells). They play an important role in innate immunity by identifying the conserved pathogen-associated molecular pattern (PAMP) derived from diverse pathogens [75]. TLR2 and TLR4 have also been studied to recognize the viral structural proteins, which results in the production of inflammatory cytokines against the viral infection [76,77,78]. TLR3 also detects viral infection and initiates an innate immune signalling pathway [79]. We carried out protein–protein docking to compute the binding affinity of the chimeric vaccine for TLRs. The docking analysis revealed the lowest (negative) S-scores (high-affinity) for the constructed vaccine–TLRs complexes. In addition, ionic and hydrogen bonding between the vaccine construct–TLRs interface also indirectly supported the formation of stable complexes [80], implying the potential of the designed vaccine construct to trigger an appropriate downstream immune pathway. Molecular dynamics simulation is a reliable approach for capturing motion at the atomic level, which is very difficult to accomplish by employing experimental methods [81]. The structural stability and compactness of the vaccine construct–TLRs complex were validated using the RMSD, RMSF, and Rg descriptors. The SASA profile of complexes indicated structural adjustments caused by the vaccine construct (ligand) binding to the receptor. Besides, H-bond analysis supported the formation of several long-lasting H-bonds between vaccine construct–TLRs interface that could play an essential role in complex stability [82]. Consistent with the docking analysis, binding free energy calculation with MM/GBSA method showed negative ∆G scores (energetically favourable binding) for peptides–HLA molecule complexes and modelled vaccine–TLRs complexes [83].

The C-ImmSim server predicted high titers of neutralizing antibody production following in silico immunization, which is essential to combat the viral infection. Immune simulation findings also indicated substantial CTLs and IFN-γ levels, hence induction of long-lasting cellular and adaptive immune responses against CCHFV infection. These outcomes are comparable to other studies that designed multi-epitope vaccine constructs using B-cell, CTL, and HTL epitopes [32,63]. Nonetheless, experimental validation of the constructed vaccine is required to support the findings of the current investigation.

The present study represents an alternative approach to designing a vaccine based on a multi-epitope vaccine ensemble comprising antigenic components of CCHFV proteins (Asia-1 genotype) to tackle the antigenic complexity. Although numerous immunoinformatics-based methods were used, and the designed vaccine construct is predicted to be immunogenic; nevertheless, the extent of protection from the viral infection is unknown. Following the literature, the order and spacing of the CTL and B-cell epitopes were provided; still, this would require more proof to obtain the best possible immunogenicity with CTL and B-cell epitopes. The next step would be in vitro immunological assays to establish the immunogenicity of the designed vaccine construct and perform challenge-protection clinical experiments to validate the strategy.

## 5. Conclusions

Despite a significant worldwide health burden associated with CCHF, no approved vaccine or therapeutics exists to protect and treat the disease. The current study aimed to design a multi-epitope vaccine ensemble for CCHFV infection (Asia-1 genotype). Multiple immunodominant B-cell and CTL epitopes were mapped from the viral structural glycoproteins (G1 and G2) and were assembled into a non-allergen and antigenic multi-epitope-based vaccine construct. Various linkers and an adjuvant sequence were added to improve the immunological response, stability, and efficiency of the designed vaccine construct. The developed vaccine construct possesses relevant structural, physicochemical, and immunological properties to elicit humoral and cellular immune responses efficiently. Nonetheless, experimental validation of safety and effectiveness, as well as preclinical investigations of the designed vaccine, required prior to human vaccination.

## Figures and Tables

**Table 1:** Shortlisted linear B-Cell Epitopes from G1 and G2 of CCHFV. These epitopes are predicted as non-allergen (AllerTOP v.2.0) and non-toxic (ToxinPred).

Start	End	Peptide	Length	VexiJen	Percent Identity
G1 protein					
285	296	DDCISRTQLLRT	12	0.42	50.00% (XP_006711865.1)
415	444	QHFLKDNLIDLGCPNIPLLGKMAIYICRMS	30	0.42	37.50% (XP_047304331.1)
452	481	AFLFWFSFGYVITCILCKVIFYLLIVVGTL	30	0.57	43.75% (XP_047276342.1)
G2 protein					
22	40	CWGVGTGCTCCGLDVKDLF	19	1.37	60.00% (NP_001305465.1)
42	53	DYMFVKWKVEYI	12	1.00	83.33% (CAA69330.1)

**Table 2:** Shortlisted CTL epitopes from G1 and G2 of CCHFV. The prediction score threshold was set at >0.75000. Shortlisted epitopes are predicted as non-allergen (AllerTOP v.2.0) and non-toxic (ToxinPred).

Position	Peptide Sequence	Predicted MHC Binding Affinity	Binding Affinity Rescale Score	C-Terminal Cleavage Affinity	Transport Affinity	Prediction Score	VexiJen	BLASTp % Identity (Accession No.)
GP 1 protein								
234	KQNDRCTLV	0.5317	0.7926	0.8507	0.5440	0.9474	1.5407	66.67% (MBY87633.1)
453	FLFWFSFGY	0.4787	0.7136	0.9715	3.1150	1.0151	1.1001	77.78% (NP_114125.1)
473	YLLIVVGTL	0.5793	0.8636	0.9660	0.8670	1.0518	0.8774	63.64% (XP_047286234.1)
589	LLTVSLSPV	0.6810	1.0151	0.9670	0.3490	1.1776	1.3397	87.50% (AAL65133.2)
623	FVLGSILFI	0.8208	1.2236	0.5436	0.6440	1.3373	0.6538	57.14% (NP_001308089.1)
G2 protein								
55	TEAIVCVEL	0.4966	1.2306	0.9690	0.9160	1.4217	1.1768	75.00% (KAI2525983.1)

**Table 3:** Estimated immunological and physicochemical properties of the designed vaccine construct.

Property	Result	Indication
No. of amino acid	324	
Sol-Pro	0.733538	Soluble
Protein Sol	0.490	Soluble
Molecular weight	33888.65 Da	Suitable
Formula	C_1435_H_2228_N_374_O_428_S_13_	
Theoretical pI	5.01	Acidic
Instability index	24.69	(Stable)
Aliphatic index	102.75	(Thermostable)
Total number of negatively charged residues (Asp + Glu)	37	
Total number of positively charged residues (Arg + Lys)	30	
Half-Life	30 h (mammalian reticulocytes, in vitro), >20 h (yeast, in vivo), >10 h (Escherichia coli, in vivo).	
Allergenicity	AllerTOP v.2.0 (Non-allergen), AllergenFP v.1.0 (Non-allergen)	Non-allergen
Antigenicity	VexiJen v2.0 (0.5163), ANTIGENpro (0.84)	Antigen
TM helices	0	Suitable

**Table 4:** MM/GBSA free energy calculations and individual free energy components of the selected peptide–HLA molecule complexes.

Peptide	∆_VDW_ (kcal/mol)	∆_EEL_ (kcal/mol)	∆_EGB_ (kcal/mol)	∆_ESURF_ (kcal/mol)	∆_G TOTAL_ (kcal/mol)
3L3D	−78.36 ± 0.15	−347.03 ± 1.02	384.95 ± 0.87	−15.03 ± 0.01	−55.48 ± 0.17
5HHP	−88.17 ± 0.11	−357.42 ± 0.83	389.09 ± 0.69	−13.87 ± 0.09	−45.37 ± 0.17
KQNDRCTLV	−70.69 ± 0.14	−335.63 ± 0.90	367.02 ± 0.88	−10.77 ± 0.01	−50.08 ± 0.16
FLFWFSFGY	−82.87 ± 0.22	−196.62 ± 0.85	234.37 ± 0.80	−12.37 ± 0.02	−57.50 ± 0.23
YLLIVVGTL	−84.64 ± 0.14	−199.13 ± 0.65	228.07 ± 0.58	−13.13 ± 0.02	−68.84 ± 0.20
LLTVSLSPV	−71.58 ± 0.19	−132.33 ± 0.61	168.95 ± 0.58	−10.39 ± 0.02	−45.36 ± 0.19
FVLGSILFI	−71.97 ± 0.18	−154.77 ± 1.36	193.63 ± 1.31	−10.58 ± 0.02	−43.70 ± 0.24
TEAIVCVEL	−78.05 ± 0.12	−127.94 ± 1.19	163.53 ± 1.11	−12.74 ± 0.01	−55.21 ± 0.18

VDW = Van der Waals, EEL = Electrostatic energy, EGB = Polar solvation energy, ESURF = non-polar solvation energy.

**Table 5:** MM/GBSA free energy calculations and individual free energy components of the constructed vaccine–TLR2/3/4 complex. Differences (Complex–Receptor–Ligand).

Complex Name	∆_VDW_ (kcal/mol)	∆_EEL_ (kcal/mol)	∆_EGB_ (kcal/mol)	∆_ESURF_ (kcal/mol)	∆_G TOTAL_ (kcal/mol)
Modelled vaccine–TLR2	−150.27 ± 0.19	−466.56 ± 1.53	556.00 ± 1.50	−19.95 ± 0.02	−80.79 ± 0.17
Modelled vaccine–TLR3	−128.81 ± 0.24	−376.39 ± 1.40	442.65 ± 1.37	−16.87 ± 0.03	−79.43 ± 0.20
Modelled vaccine–TLR4	−189.68 ± 0.22	172.25 ± 1.70	−40.30 ± 1.60	−26.51 ± 0.02	−84.24 ± 0.22

VDW = Van der Waals, EEL = Electrostatic energy, EGB = Polar solvation energy, ESURF = non-polar solvation energy.

## Data Availability

Not applicable.

## Supplementary Materials

The following supporting information can be downloaded at https://www.mdpi.com/article/10.3390/vaccines11010061/s1. Figure S1: In silico immune simulation of an infection challenge, comprised of a virus responding to the sequence of Asia-1 genotype of CCHFV proteins covered by the multi-epitope construct, was simulated on day 350; Figure S2: Root Mean Square Deviation (RMSD) analysis of the selected epitopes (KQNDRCTLV, FLFWFSFGY, YLLIVVGTL, LLTVSLSPV, FVLGSILFI) docked with the HLA-A*02:01 molecule and epitope TEAIVCVEL docked with HLA-B*44:02 molecule. The 3L3D (EEAGRAFSF and HLA-B*44:02 complex) and 5HHP (GILEFVFTL and HLA-A*02:01 complex) were selected as reference systems; Table S1: Population coverage calculation result; Table S2: GalaxyRefine output for the submitted initial model of vaccine constructs predicted by the I-TASSER server; Table S3: Docking score (DS) and RMSD of the selected peptides with the HLA-B*44:02 and HLA-A*02:01 alleles; Table S4: Molecular docking calculations and interactions details between selected CTL epitopes and respective HLA class I allele; Table S5: Molecular docking between selected CTL peptides and mouse MHC-I molecules; Table S6: Atom–atom interactions across TLR2-designed vaccine construct interface obtained using PDBsum server; Table S7: Atom–atom interactions across TLR3-designed vaccine construct interface obtained using PDBsum server; Table S8: Atom–atom interactions across TLR4–designed vaccine construct interface obtained using PDBsum server; Table S9: Fraction of hydrogen bonds at the designed vaccine construct–TLRs interface during the 100 ns simulation timescale (with occupancy ≥ 2%)
