# Supplementary material for: An Immunoinformatics Approach to Design a Potent Multi-Epitope Vaccine against Asia-1 Genotype of Crimean–Congo Haemorrhagic Fever Virus Using the Structural Glycoproteins as a Target
Source: Vaccines (Basel). 2022 Dec 27;11(1):61. doi: 10.3390/vaccines11010061 (PMC9867508; doi:10.3390/vaccines11010061)
Supplement: Supplementary file 1 [file vaccines-11-00061-s001.zip › Supplimentry File 1.pdf]

Supporting Information

An Immunoinformatics Approach to Design a Potent Multi-Epitope Vaccine Against Asia-1 Genotype of Crimean-Congo Haemorrhagic Fever Virus Using the Structural Glycoproteins as a Target

Syed Zawar Shah[1†], Basit Jabbar[1], Muhammad Usman Mirza[2], Muhammad Waqas[3,4†], Shahkaar Aziz[5], Sobia Ahsan Halim[4], Amjad Ali[3*], Shazia Rafique[1], Muhammad Idrees[1], Asaad Khalid[6,7], Ashraf N. Abdalla[8], Ajmal Khan[4*], and Ahmed Al-Harrasi[4*]

1. Centre of Excellence in Molecular Biology, University of the Punjab, 53700, Punjab, Pakistan
2. Department of Chemistry and Biochemistry, University of Windsor, ON, Canada
3. Department of Biotechnology and Genetic Engineering, Hazara University Mansehra 21120, Mansehra, Pakistan
4. Natural and Medical Sciences Research Center, University of Nizwa, Birkat-ul-Mouz 616, Nizwa, Oman
5. Institute of Biotechnology and Genetic Engineering, the University of Agriculture Peshawar, Pakistan
6. Substance Abuse and Toxicology Research Center, Jazan University, P.O. Box: 114, Jazan 45142, Saudi Arabia
7. Medicinal and Aromatic Plants and Traditional Medicine Research Institute, National Center for Research, P. O. Box 2404, Khartoum, Sudan
8. Department of Pharmacology and Toxicology, College of Pharmacy, Umm Al-Qura University, Makkah 21955, Saudi Arabia

†These authors have contributed equally to this work.

* Correspondence: amjad.camb@pu.edu.pk (A.A.); ajmalkhan@unizwa.edu.om (A.K); aharrasi@unizwa.edu.om (A.A-H)

Table of content

Figure S1 In silico immune simulation of an infection challenge, comprised of a virus responding to the sequence of Asia-1 genotype of CCHFV proteins covered by the multi-epitope construct, was simulated on day 350. (A) B lymphocytes population per entity-state (i.e., showing counts for active, presenting on class II, internalized the Ag, duplicating and anergic (B) Plasma B lymphocytes count sub-divided per isotype (IgM, IgG1 and IgG2) (C) CD4+ T-helper (TH) lymphocytes in several forms, i.e., active, duplicating (in the mitotic cycle), resting (not active), and anergic (D) CD4 T-regulatory lymphocytes count. Both total, memory and per entity-state counts are plotted here (E) Total count, internalized, presenting on MHC class-II, active and resting macrophages. (F) Dendritic cells can present antigenic peptides on both MHC class-I and class-II molecules. The curves show the total number broken down to active, resting, internalized, and presenting the ag.

Figure S2 Root Mean Square Deviation (RMSD) analysis of the selected epitopes (KQNDRCTLV, FLFWFSFGY, YLLIVVGTL, LLTVSLSPV, FVLGSILFI) docked with

the HLA-A*0201 molecule and epitope TEAIVCVEL docked with HLA-B*4402 molecule. The 3L3D (EEAGRAFSF and HLA-B*4402 complex) and 5HHP (GILEFVFTL and HLA-A*0201 complex) were selected as reference systems.

Table S1 Population coverage calculation result

Table S2 GalaxyRefine output for the submitted initial model of vaccine constructs predicted by the I-TASSER server. (Letter in bold indicates the final model selected for further analysis).

Table S3 Docking score (DS) and RMSD of the selected peptides with the HLA-B*4402 and HLA-A*0201 alleles.

Table S4 Molecular docking calculations and interactions details between selected CTL epitopes and respective HLA class I allele.

Table S5 Molecular docking between selected CTL peptides and mouse MHC-I molecules

Table S6 Atom-atom interactions across TLR2–designed vaccine construct interface obtained using PDBsum server

Table S7 Atom-atom interactions across TLR3–designed vaccine construct interface obtained using PDBsum server

Table S8 Atom-atom interactions across TLR4–designed vaccine construct interface obtained using PDBsum server

Table S9 Fraction of hydrogen bonds at the designed vaccine construct–TLRs interface during the 100ns simulation timescale (with occupancy ≥ 2%)

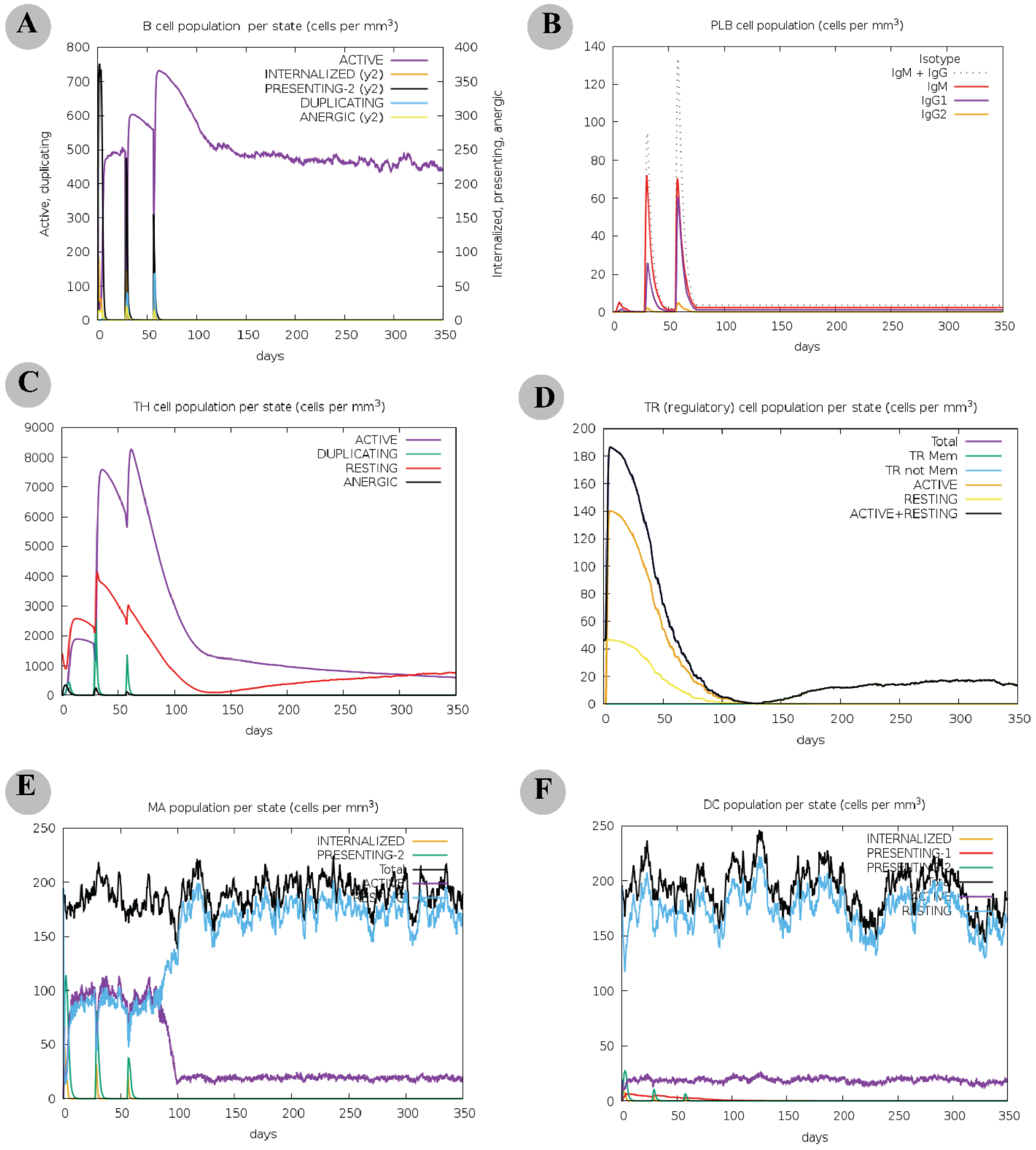

Figure S1. *In silico* immune simulation of an infection challenge, comprised of a virus responding to the sequence of Asia-1 genotype of CCHFV proteins covered by the multi-epitope construct, was simulated on day 350. **(A)** B lymphocytes population per entity-state (i.e., showing counts for active, presenting on class II, internalized the Ag, duplicating and anergic **(B)** Plasma B lymphocytes count sub-divided per isotype (IgM, IgG1 and IgG2) **(C)** CD4+ T-helper (TH) lymphocytes in several forms, i.e., active, duplicating (in the mitotic cycle), resting (not active), and anergic **(D)** CD4 T-regulatory lymphocytes count. Both total, memory and per entity-state counts are plotted here **(E)** Total count, internalized, presenting on MHC class-II, active and resting macrophages. **(F)** Dendritic cells can present antigenic peptides on both MHC class-I and class-II molecules. The curves show the total number broken down to active, resting, internalized, and presenting the ag.

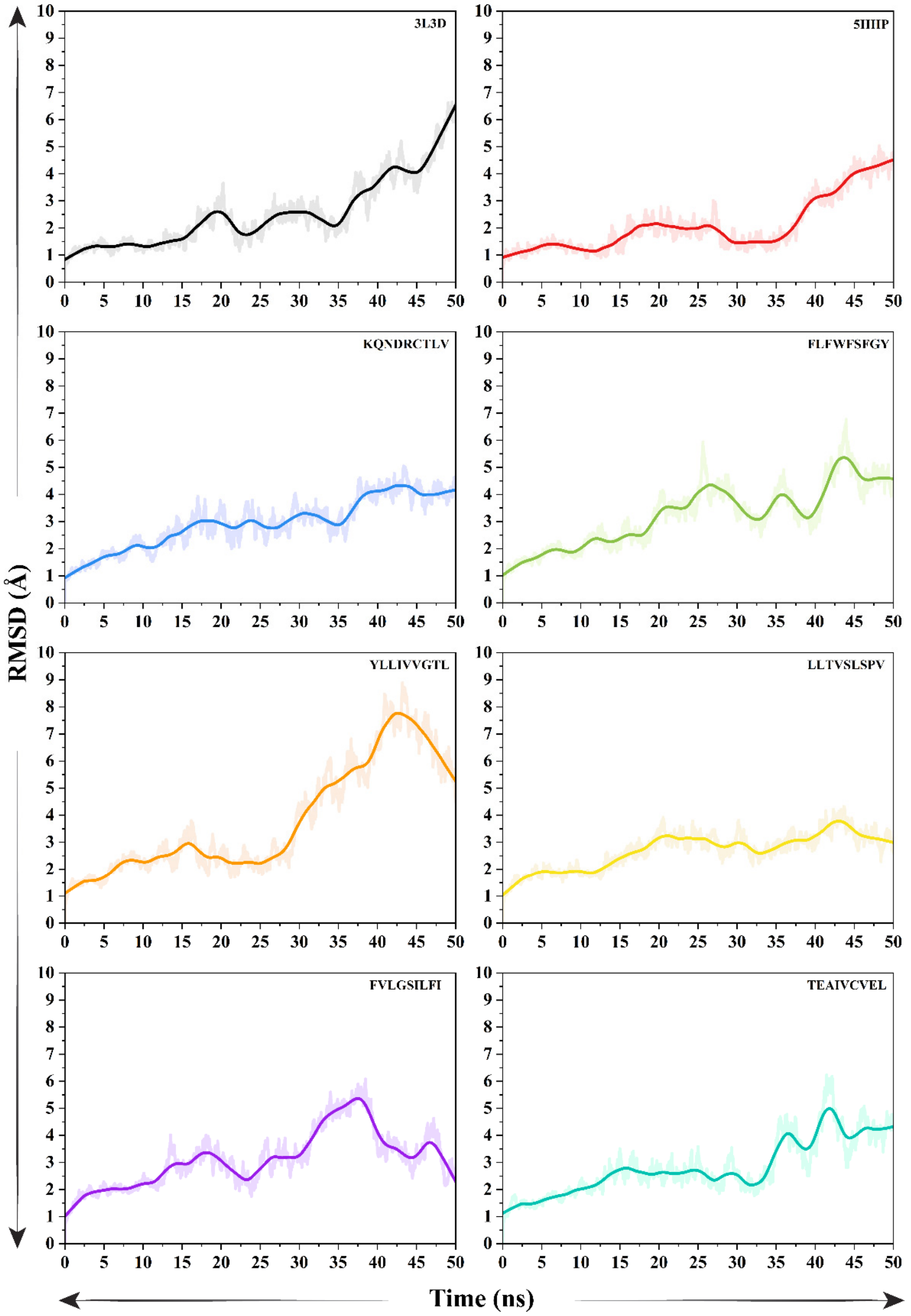

Figure S2. Root Mean Square Deviation (RMSD) analysis of the selected epitopes (KQNDRCTLV, FLFWFSFGY, YLLIVVGTL, LLTVSLSPV, FVLGSILFI) docked with the HLA-A*0201 molecule and epitope TEAIVCVEL docked with HLA-B*4402 molecule. The 3L3D (EEAGRAFSF and HLA-B*4402 complex) and 5HHP (GILEFVFTL and HLA-A*0201 complex) were selected as reference systems.

Table S1. Population coverage calculation result

	Class I		
population/area	coverage[a]	average hit[b]	pc90[c]
East Asia	35.99%	1.43	0.16
Northeast Asia	30.33%	1.5	0.14
Pakistan	49.85%	1.56	0.71
South Asia	31.98%	1.44	0.15
Northeast Asia	29.51%	1.44	0.14
Southeast Asia	45.54%	2.28	0.18
World	60.52%	3.07	0.25
[a]projected population coverage			
[b]average number of epitope hits / HLA combinations recognized by the population			
[c]minimum number of epitope hits / HLA combinations recognized by 90% of the population			

Table S2. GalaxyRefine output for the submitted initial model of vaccine constructs predicted by the I-TASSER server. (Letter in bold indicates the final model selected for further analysis).

Model	GDT-HA	RMSD	MolProbity	Clash score	Poor rotamers	Rama favored
Initial	1.0000	0.000	3.227	9.7	13.2	66.8
MODEL 1	0.9275	0.469	2.423	20.6	0.8	87.3
MODEL 2	0.9360	0.471	2.494	25.4	0.8	87.9
**MODEL 3**	0.9267	0.463	2.501	24.5	0.8	87.0
MODEL 4	0.9174	0.490	2.498	24.7	0.8	87.3
MODEL 5	0.9282	0.471	2.438	20.4	0.8	86.3

Table S3. Docking score (DS) and RMSD of the selected peptides with the HLA-B*4402 and HLA-A*0201 alleles.

Peptide	Allele	Docking Score (Kcal/mol)	RMSD (Å)
EEAGRAFSF (3L3D)	HLA-B*4402	–108.89	0.416
GILEFVFTL (5HHP)	HLA-A*0201	–40.53	0.900
KQNDRCTLV	HLA-A*0201	–60.21	0.825
FLFWFSFGY	HLA-A*0201	–56.85	1.140
YLLIVVGTL	HLA-A*0201	–37.35	0.570
LLTVSLSPV	HLA-A*0201	–53.81	0.767
FVLGSILFI	HLA-A*0201	–55.69	0.457
TEAIVCVEL	HLA-B*4402	–37.83	0.819

Table S4. Molecular docking calculations and interactions details between selected CTL epitopes and respective HLA class I allele.

Peptide	Receptor Atoms	Receptor Residues	Receptor residues No	Peptide Atoms	Peptide Resides	Peptide residue No	Distance (Å)
3L3D	OH	TYR	7	N	GLU	1	3.1
	OH	TYR	9	OE1	GLU	2	2.55
	NZ	LYS	45	OE2	GLU	2	2.73
	NH2	ARG	62	OE1	GLU	1	3.02
	OE2	GLU	63	N	GLU	2	2.91
	OD1	ASN	70	N	ALA	6	3
	OD1	ASN	77	N	PHE	9	2.74
	OH	TYR	84	O	PHE	9	2.66
	OH	TYR	99	OE1	GLU	2	2.51
	OH	TYR	99	N	ALA	3	3.05
	OG1	THR	143	O	PHE	9	2.67
	NZ	LYS	146	OXT	PHE	9	2.83
	NE1	TRP	147	O	SER	8	2.83
	OH	TYR	159	O	GLU	1	2.6
	OG	SER	167	N	GLU	1	3.03
	NH2	ARG	170	OE2	GLU	1	2.79
	OH	TYR	171	N	GLU	1	2.7
**Receptor Non-Bonded Contacts:** MET5, TYR7, TYR9, THR24, LYS45, TYR59, ARG62, GLU63, ILE66, SER67, ASN70, THR73, TYR74, GLU76, ASN77, THR80, TYR84, ILE95, ARG97, TYR99, ASP116, TYR123, THR143, LYS146, TRP147, VAL152, GLN155, ASP156, TYR159, LEU163, SER167, ARG170, and TYR171.				**Peptide Non-Bonded Contacts:** GLU1, GLU2, GLY4, ALA3, ALA6, PHE7, SER8, and PHE9.			
5HHP	OH	TYR	7	N	GLY	1	2.83
	OE1	GLU	63	N	ILE	2	3.09
	NH1	ARG	65	OE2	GLU	4	2.53
	NZ	LYS	66	O	ILE	2	2.87
	OD1	ASP	77	N	LEU	9	2.93
	OH	TYR	99	N	LEU	3	2.98
	OG1	THR	143	O	LEU	9	2.69
	NZ	LYS	146	O	THR	8	3.07
	NZ	LYS	146	OG1	THR	8	3.15
	NE1	TRP	147	O	THR	8	2.99
	OH	TYR	159	O	GLY	1	2.62
	OH	TYR	171	N	GLY	1	2.71
**Receptor Non-Bonded Contacts:** TYR7, GLU63, ARG65, LYS66, VAL67, HIS70, THR73, ASP77, THR80, LEU81, TYR84, ARG97, TYR99, HIS114, TYR116, THR143, LYS146, TRP147, VAL152, LEU156, TYR159, TRP167, and TYR171.				**Peptide Non-Bonded Contacts:** ILE 2, GLY1, GLU4, LEU3, VAL6, PHE7, LEU9, and THR8			
KQNDRCTLV	OE2	GLU	63	NE2	GLN	2	2.92
	NZ	LYS	66	O	ASP	4	2.93
	NE2	HIS	70	OD2	ASP	4	3.07
	NH2	ARG	97	O	VAL	9	2.81
	OH	TYR	159	O	ASN	3	2.84
	NE1	TRP	167	O	LYS	1	2.67
**Receptor Non-Bonded Contacts:** TYR59, GLU63, LYS66, HIS70, THR73, HIS74, ASP77, ARG97, HIS114, TYR116, TYR123, THR143, TRP147, LEU156, TYR159, TRP167, and ARG170.				**Peptide Non-Bonded Contacts:** LYS1, GLN2, ASP4, ASN3, LEU8, VAL9, THR7, CYS6, and ARG5.			
FLFWFSFGY	OG1	THR	73	OH	TYR	9	2.79
	OG1	THR	73	OH	TYR	9	2.79
	OD1	ASP	77	N	TYR	9	2.86
	NE1	TRP	147	OG	SER	6	2.95

**Receptor Non-Bonded Contacts:** MET5, TYR7, PHE33, MET45, TYR59, GLU63, LYS66, VAL67, THR73, HIS74, ASP77, ARG97, TYR99, GLY100, TYR116, TYR123, ILE124, THR143, TRP147, VAL152, GLN155, LEU156, TYR159, CYS164, and TRP167.				**Peptide Non-Bonded Contacts:** LEU2 PHE1, PHE3, PHE7, TYR9, GLY8, TRP4, SER6, and PHE5.			
**YLLIVVGTL**	OG1	THR	73	OH	TYR	9	2.79
	OG1	THR	73	OH	TYR	9	2.79
	OD1	ASP	77	N	TYR	9	2.86
	NE1	TRP	147	OG	SER	6	2.95
**Receptor Non-Bonded Contacts:** MET5, TYR7, PHE33, MET45, TYR59, GLU63, LYS66, VAL67, THR73, HIS74, ASP77, ARG97, TYR99, GLY100, TYR116, TYR123, ILE124, THR143, TRP147, VAL152, GLN155, LEU156, TYR159, CYS164, and TRP167.				**Peptide Non-Bonded Contacts:** LEU2, PHE1, PHE3, PHE7, TYR9, GLY8, TRP4, SER6, and PHE5.			
**LLTVSLSPV**	OE2	GLU	63	N	LEU	1	2.76
	NE2	HIS	70	O	VAL	4	3.33
	OG1	THR	80	O	PRO	8	2.88
	OG1	THR	143	OXT	VAL	9	2.83
	NE1	TRP	147	OG	SER	7	2.75
**Receptor Non-Bonded Contacts:** MET5, ARG6, TYR7, GLY26, PHE33, VAL34, TYR59, GLU63, LYS66, HIS70, THR73, VAL76, ASP77, THR80, TYR84, ARG97, TYR99, GLY100, HIS114, TYR123, THR143, LYS146, TRP147, VAL152, GLN155, LEU156, TYR159, CYS164, and TYR171.				**Peptide Non-Bonded Contacts:** LEU2, LEU1, VAL4, THR3, LEU6, PRO8, VAL9 SER7, and SER5.			
**FVLGSILFI**	NZ	LYS	66	O	GLY	4	2.85
	NE2	HIS	70	O	LEU	3	3.09
	NE1	TRP	147	O	LEU	7	2.8
**Receptor Non-Bonded Contacts:** MET5, TYR7, LYS66, HIS70, THR73, HIS74, ASP77, THR80, TYR84, ARG97, TYR99, HIS114, THR143, LYS146, TRP147, VAL152, LEU156, TYR159, THR163, CYS164, and TRP167.				**Peptide Non-Bonded Contacts:** PHE1, LEU3, GLY4, PHE8, ILE9, VAL2, ILE6, and LEU7.			
**TEAIVCVEL**	OE1	GLU	63	N	THR	1	2.75
	OE1	GLU	63	OG1	THR	1	2.74
	NE1	TRP	147	O	GLU	8	2.85
	OG	SER	167	O	GLU	2	2.98
	OH	TYR	171	N	GLU	2	2.69
	OH	TYR	171	O	GLU	2	2.72
**Receptor Non-Bonded Contacts:** TYR7, VAL34, ILE52, GLU55, TYR59, TRP60, GLU63, ILE66, ASN70, THR73, TYR74, ASN77, THR80, TYR99, THR143, LYS146, TRP147, VAL152, TYR159, LEU163, CYS164, SER167, and TYR171.				**Peptide Non-Bonded Contacts:** ALA3, THR1, GLU2, VAL5, GLU8, LEU9, CYS6 VAL7, and ILE4.			

Table S5. Molecular docking between selected CTL peptides and mouse MHC-I molecules

**PDB ID: 1YN6 (classical) H2-Db**								
**Peptide**	**Docking Score**	**RMSD**	**Bond**	**Protein Residues**	**Peptide Residues**	**Energy (kcal/mol)**	**Distance (Å)**	**Bond-Frequency**
KQNDRCT LV	-40.18	0.92	IH	Glu9	Arg5	-15.18	3.06	4
			H	Lys66	Asn3	-4.20	2.82	1
			H	Gln70	Arg5	-1.00	3.04	1
			H	Asn80	Val9	-3.90	2.94	1
			IH	Lys146	Val9	-32.39	2.76	4
			H	Trp147	Val9	-6.50	2.92	1
			H	His155	Cys6	-3.50	2.77	1
			H	Tyr156	Leu8	-1.10	3.08	1
			H	Gly162	Lys1	-5.30	2.73	1
			H	Glu163	Asn3	-2.70	2.86	1
			IH	Glu166	Lys1	-48.82	2.76	6
			H	Glu166	Asn3	-8.00	2.95	1

FLFWFSFGY	-40.37	1.86	H	Gln65	Phe1	-12.50	3.01	2
			H	Asn80	Tyr9	-4.90	2.74	1
			H	Tyr84	Tyr9	-3.40	3.01	2
			IH	Lys146	Tyr9	-11.76	3.12	3
			H	Trp147	Tyr9	-7.20	2.82	1
			H	Ser150	Ser6	-1.50	2.84	1
			H	His155	Phe3	-0.80	2.88	1
YLLIVVGTL	-51.49	0.68	H	Gln70	Val6	-3.10	2.89	2
			H	Trp73	Thr8	-0.70	2.94	1
			H	Gln97	Val6	-3.60	2.93	1
			IH	Lys146	Leu9	-37.37	2.68	4
			H	Trp147	Thr8	-0.70	2.93	1
			H	His155	Val5	-6.50	2.80	1
			IH	Glu163	Tyr1	-16.56	2.74	2
			IH	Glu166	Tyr1	-32.24	2.76	4
LLTVSLSPV	-36.38	1.75	H	Gln65	Thr3	-2.60	2.92	2
			H	Trp73	Ser7	-0.80	2.80	1
			H	Asn80	Val9	-2.60	2.94	1
			H	Tyr84	Val9	-4.30	2.56	1
			IH	Lys146	Val9	-21.52	2.84	3
			H	Trp147	Pro8	-0.90	2.79	1
			H	His155	Leu6	-5.80	2.86	1
FVLGSILFI	-35.10	1.92	H	Gly69	Leu3	-0.60	3.41	1
			H	Asn80	Ile9	-3.80	2.79	1
			H	Tyr84	Ile9	-4.20	2.67	1
			IH	Lys146	Ile9	-21.00	2.99	3
			H	His155	Phe1	-6.70	3.09	1
			H	Ala158	Phe1	-3.60	2.78	1
TEAIVCVEL	-39.72	2.56	H	Gln65	Thr1	-2.50	2.61	1
			H	Trp73	Val7	-1.90	2.89	1
			H	Ser77	Leu9	-1.10	3.28	1
			H	Asn80	Glu8	-5.90	2.78	1
			H	Asn80	Leu9	-6.80	2.77	1
			H	Tyr84	Leu9	-5.20	2.65	1
			H	Thr143	Leu9	-2.50	2.60	1
			IH	Lys146	Glu8	-26.83	2.86	4
			IH	Lys146	Leu9	-11.47	3.15	3
			H	Trp147	Glu8	-1.90	2.83	1
			H	His155	Val5	-4.90	2.83	1
			H	Tyr156	Val7	-0.60	2.85	1
**PDB ID: 6JQ3 (classical) H2-Kb**								
TEAIVCVEL	-41.53	1.48	H	Glu24	Gln2	-3.80	2.90	1
			IH	Arg62	Asp4	-15.23	3.07	4
			H	Lys66	Gln2	-8.10	2.78	1
			H	Gly69	Lys1	-8.40	2.83	1
			H	Gln72	Lys1	-6.50	2.70	1
			IH	Glu152	Lys1	-28.28	2.71	4
			H	Thr163	Asp4	-1.70	2.69	1
			IH	Glu166	Arg5	-35.72	2.82	6
			H	Glu166	Thr7	-3.60	2.66	1
			H	Trp167	Asp4	-5.30	2.80	1
			H	Arg169	Leu8	-6.90	2.82	1
			IH	Lys173	Val9	-41.75	2.67	4
TEAIVCVEL	-44.40	1.78	H	Tyr7	Tyr9	-4.80	2.61	1
			H	Arg62	Phe7	-4.40	2.77	1
			IH	Lys66	Tyr9	-24.63	2.70	2

			H	Asn70	Tyr9	-3.20	2.71	1
			H	Lys146	Phe1	-8.20	2.86	1
			H	Gln149	Phe1	-9.80	2.80	2
			H	Arg155	Phe3	-0.80	3.05	1
			H	Tyr159	Tyr9	-1.70	2.61	1
TEAIVCVEL	-39.64	1.15	H	Arg62	Val6	-4.20	2.79	1
			H	Lys66	Ile4	-11.40	2.79	1
			H	Ser73	Tyr1	-2.40	2.81	1
			IH	Asp77	Tyr1	-26.87	2.77	3
			H	Tyr116	Tyr1	-3.10	2.66	1
			H	Arg155	Leu2	-2.40	2.85	1
			H	Arg155	Leu3	-2.70	2.76	1
			H	Glu166	Thr8	-1.40	2.69	1
			H	Trp167	Gly7	-2.50	2.91	1
			IH	Arg169	Leu9	-28.73	3.04	6
			H	Arg170	Leu9	-0.70	3.40	1
TEAIVCVEL	-49.25	0.92	H	Glu24	Thr3	-1.30	2.71	1
			H	Arg62	Leu6	-1.30	2.84	1
			H	Arg62	Ser7	-8.30	2.84	2
			H	Lys66	Val4	-12.20	2.76	1
			IH	Asp77	Leu1	-26.31	2.84	4
			H	Ser99	Thr3	-1.60	2.93	1
			H	Tyr116	Leu1	-3.30	2.71	1
			H	Tyr159	Ser5	-2.20	2.76	1
			H	Trp167	Leu6	-1.70	2.73	1
TEAIVCVEL	-36.77	0.85	H	Tyr7	Ile9	-4.70	2.61	1
			I	Arg62	Ile9	-2.58	3.34	1
			IH	Lys66	Ile9	-15.16	2.81	2
			H	Ser73	Phe1	-4.50	2.95	1
			IH	Asp77	Phe1	-18.98	2.81	3
			H	Arg155	Leu3	-9.50	2.88	2
			H	Arg155	Ile6	-5.30	2.78	1
			H	Tyr159	Ile9	-1.60	2.63	1
TEAIVCVEL	-34.10	1.10	IH	Arg62	Leu9	-32.02	2.97	7
			IH	Lys66	Leu9	-14.94	2.69	2
			H	Asn70	Val7	-3.90	2.87	1
			H	Tyr116	Cys6	-2.30	3.30	1
			H	His145	Thr1	-1.70	2.89	1
			H	Lys146	Glu2	-0.60	3.26	1
			H	Lys146	Ala3	-3.50	2.86	1
			H	Gln149	Thr1	-16.20	2.83	2
			H	Glu152	Cys6	-8.70	3.03	2
			IH	Arg155	Glu8	-35.32	3.00	6
			H	Trp167	Leu9	-2.70	2.80	1
**PDB ID: 7LFK (non-classical) H2-M3**								
TEAIVCVEL	-51.22	1.34	IH	Glu62	Lys1	-29.07	2.77	4
			H	Glu62	Gln2	-7.40	2.79	1
			H	Asn77	Val9	-5.80	2.95	2
			H	Thr143	Val9	-2.30	2.60	1
			H	Ala150	Arg5	-4.60	2.79	1
			H	Ala158	Asn3	-4.70	3.05	2
			H	Gly162	Lys1	-10.80	2.79	1
			IH	Glu166	Lys1	-16.49	2.62	2
TEAIVCVEL	-52.97	1.61	H	Ser73	Ser6	-1.50	2.85	1
			H	Asn77	Phe7	-1.90	2.85	1
			H	Tyr155	Trp4	-2.30	2.96	1

			H	Ala158	Phe1	-10.20	2.91	1
			IH	Glu161	Phe1	-28.70	2.81	3
TEAIVCVE L	-48.01	1.01	H	Ala76	Leu9	-0.50	3.54	1
			H	Asn77	Gly7	-0.50	2.99	1
			IH	Arg79	Leu9	-27.06	3.09	6
			H	Thr80	Thr8	-1.50	2.95	1
			H	Arg146	Val6	-7.10	2.76	1
			H	Arg146	Thr8	-5.80	2.94	2
			H	Ala158	Tyr1	-0.50	2.86	1
			A	Glu163	Tyr1	-0.50	3.43	1
			H	Glu166	Tyr1	-5.20	2.62	1
TEAIVCVE L	-36.17	1.02	H	Asn77	Ser5	-2.00	2.72	1
			H	Asn77	Ser7	-2.30	2.86	1
			H	Thr80	Val9	-2.10	2.71	1
			H	Arg146	Ser7	-4.10	2.74	1
			IH	Arg146	Val9	-24.39	2.97	5
			H	Ala158	Leu1	-3.50	2.68	1
			IH	Glu163	Leu1	-18.17	2.58	2
TEAIVCVE L	-43.89	0.70	H	Lys66	Leu3	-9.00	2.80	1
			H	Asn77	Ile9	-4.80	2.75	1
			H	Tyr114	Ile9	-4.20	2.61	1
			H	Gly162	Phe1	-2.90	3.00	1
			IH	Glu166	Phe1	-24.68	2.65	2
TEAIVCVE L	-31.13	1.73	H	Asn77	Leu9	-6.60	2.83	1
			H	Thr80	Leu9	-0.60	2.55	1
			H	Thr143	Glu8	-1.00	2.65	1
			H	Arg146	Val7	-4.50	2.81	1
			IH	Arg146	Leu9	-24.79	2.75	4
			H	Thr152	Cys6	-1.70	3.20	1
			H	Glu161	Thr1	-2.20	2.62	1

Table S6. Atom-atom interactions across TLR2– designed vaccine construct interface obtained using PDBsum server

S. No	TLR2 Atoms	TLR2 Residues	TLR2 residues No	Vaccine Atoms	Vaccine Resides	Vaccine residue No	Distance (Å)-bond type (H=H-bond)
1	OE1	GLN	79	N	ARG	246	3.05-H
2	OG1	THR	127	OG1	THR	167	3.03-H
3	NZ	LYS	150	OE2	GLU	58	2.73-H
4	NE2	GLN	152	OE1	GLU	131	3.11-H
5	OD1	ASN	248	OG1	THR	126	3.14-H
6	ND2	ASN	248	O	ALA	125	2.88-H
7	NZ	LYS	252	O	GLU	68	2.81-H
8	NZ	LYS	308	O	ALA	70	2.94-H
9	O	LYS	551	NZ	LYS	318	2.78-H
10	OG	SER	553	N	VAL	321	3.06-H
11	OG	SER	553	O	VAL	321	3.2-H
12	O	GLN	554	OG1	THR	294	2.95-H
13	OE1	GLN	554	N	TRP	319	3.02-H
14	NE2	GLN	554	O	CYS	296	2.84-H
15	NE2	GLN	557	O	VAL	292	2.81-H
16	NH1	ARG	570	O	TYR	323	2.97-H
17	NH2	ARG	570	O	TYR	323	2.82-H
18	NH2	ARG	570	O	ILE	324	3.18-H

19	NZ	LYS	150	OE1	GLU	58	2.73-salt bridge
20	NZ	LYS	252	OE2	GLU	68	3.97-salt bridge
**TLR2 Non-Bonded Contacts:** ARG32, ASN33, ILE35, SER56, GLN79, ALA80, GLU103, HIS104, SER125, THR127, PHE128, LYS150, GLN152, ASN248, LEU250, LYS252, LYS308, LYS527, LYS551, SER553, GLN554, GLN557, GLY558, SER559, and ARG570				**Vaccine Non-Bonded Contacts:** HIS220, CYS245, MET247, ARG246, SER169, THR167, SER248, GLY249, PRO250, GLU58, GLU131, THR126, ALA125, GLU68, ALA70, GLY293, LYS318, VAL321, THR294, TRP319, CYS296, THR297, VAL292, TYR323, and ILE324			

Table S7. Atom-atom interactions across TLR3– designed vaccine construct interface obtained using PDBsum server

S. No	TLR3 Atoms	TLR3 Residues	TLR3 residues No	Vaccine Atoms	Vaccine Resides	Vaccine residue No	Distance (Å)
1	NZ	LYS	27	OE2	GLU	33	2.7-H
2	NE2	HIS	32	O	ALA	38	2.89-H
3	OG1	THR	78	O	GLY	310	2.72-H
4	OD2	ASP	81	OG	SER	152	2.65-H
5	NZ	LYS	102	O	PRO	311	2.75-H
6	NZ	LYS	102	O	GLY	312	2.75-H
7	ND2	ASN	105	O	GLY	151	3.05-H
8	OE1	GLN	107	NZ	LYS	153	2.92-H
9	NE2	GLN	107	O	GLY	151	2.77-H
10	OE1	GLU	175	NZ	LYS	145	2.66-H
11	NZ	LYS	200	O	GLY	199	2.74-H
12	OD1	ASN	230	NZ	LYS	94	2.87-H
13	NH1	ARG	251	OD2	ASP	202	2.77-H
14	NH2	ARG	251	OD2	ASP	202	2.73-H
15	ND2	ASN	252	OG	SER	201	2.9-H
16	OH	TYR	283	O	ASP	98	2.78-H
17	OH	TYR	307	N	ALA	70	2.97-H
18	OH	TYR	326	OE1	GLN	209	2.8-H
19	NZ	LYS	330	O	GLU	68	2.69-H
20	NZ	LYS	330	OE1	GLU	68	2.73-H
21	NH1	ARG	331	O	ALA	70	2.75-H
22	NH1	ARG	331	O	GLY	124	2.75-H
23	NH2	ARG	331	O	GLY	124	2.99-H
24	NE2	HIS	359	NH1	ARG	212	3.09-H
25	NZ	LYS	416	OE1	GLU	68	2.86-H
26	NZ	LYS	416	OE2	GLU	68	2.69-H
27	OE1	GLU	434	OG1	THR	213	2.76-H
28	OE2	GLU	434	N	GLY	251	2.81-H
29	OE1	GLU	533	OG	SER	169	2.58-H
30	ND1	HIS	565	OG	SER	169	3-H
31	NZ	LYS	589	O	SER	169	2.89-H
32	NZ	LYS	589	O	LEU	170	2.83-H
33	NZ	LYS	589	OG	SER	171	2.88-H
34	O	SER	611	OG	SER	171	2.71-H
35	NZ	LYS	613	O	SER	171	2.96-H
36	OG1	THR	638	O	PHE	13	2.84-H
37	NZ	LYS	27	OE1	GLU	33	2.7--salt bridge
38	NZ	LYS	102	OD1	ASP	313	2.76-salt bridge
39	NE2	HIS	156	OE2	GLU	92	2.88-salt bridge

40	OE1	GLU	175	NZ	LYS	145	2.66-salt bridge
41	NH2	ARG	251	OD1	ASP	202	2.73-salt bridge
42	NZ	LYS	330	OE2	GLU	68	2.73-salt bridge
43	NH1	ARG	331	OD1	ASP	72	3.84-salt bridge
44	NZ	LYS	416	OE1	GLU	68	2.69-salt bridge
45	NE2	HIS	665	OE1	GLU	15	2.7-salt bridge
**TLR3 Non-Bonded Contacts:** LYS27, VAL30, SER31, HIS32, VAL34, THR54, VAL55, ASN57, THR59, THR78, SER79, ASP81, LYS102, ASN105, GLN107, GLU127, HIS156, GLN174, GLU175, ASN180, LYS200, ASN230, ARG251, ASN252, TYR283, TYR302, TYR307, TYR326, LYS330, ARG331, GLU358, HIS359, TYR383, ILE411, LYS416, GLU434, PHE459, GLN483, GLU533, HIS565, ILE566, GLU587, LYS589, ILE590, SER611, LYS613, LEU637, THR638, HIS665, THR666, and ASN667				**Vaccine Non-Bonded Contacts:** GLU33, VAL34, THR35, ALA38, PRO309, VAL42, GLY310, PRO311, SER152, ALA148, GLY151, GLY312, ASP313, LYS153, ALA147, LYS91, GLU92, GLY199, GLY198, LYS145, LEU197, LYS94, ASP95, ASP202, SER201, LYS74, ASP98, ALA70, ALA100, SER206, ALA69, GLN209, GLU68, ASP72, GLY124, GLY71, LEU210, ARG212, THR213, GLY251, GLY249, PRO250, SER248, MET247, SER169, GLU58, SER171, LEU170, LYS14, PHE13, ASP11, and GLU15			

Table S8. Atom-atom interactions across TLR4–designed vaccine construct interface obtained using PDBsum server

S. No	TLR4 Atoms	TLR4 Residues	TLR4 residues No	Vaccine Atoms	Vaccine Resides	Vaccine residue No	Distance (Å)
1	O	VAL	32	OG1	THR	126	2.9-H
2	OE1	GLU	42	NE	ARG	212	2.77-H
3	OD2	ASP	84	NE2	GLN	209	2.83-H
4	NE	ARG	87	O	LEU	210	2.73-H
5	O	HIS	159	SG	CYS	289	3.29-H
6	NH1	ARG	234	O	GLY	199	2.79-H
7	NH1	ARG	234	OD2	ASP	202	2.72-H
8	NH2	ARG	234	O	GLY	199	2.96-H
9	NH2	ARG	264	O	VAL	193	2.89-H
10	O	ASN	265	N	GLY	295	3.01-H
11	ND2	ASN	265	OD2	ASP	203	2.84-H
12	OE1	GLU	266	N	THR	294	2.78-H
13	OE1	GLU	270	NZ	LYS	318	2.65-H
14	NH1	ARG	289	O	GLY	198	2.79-H
15	OH	TYR	296	O	TYR	314	2.69-H
16	OG	SER	317	OE1	GLU	196	2.5-H
17	OG1	THR	319	O	ASP	313	2.91-H
18	NE	ARG	322	OD2	ASP	313	2.8-H
19	NH2	ARG	322	OD2	ASP	313	2.74-H
20	NZ	LYS	341	O	PRO	311	2.78-H
21	NZ	LYS	341	O	GLY	312	2.85-H
22	OD2	ASP	502	N	LEU	90	3-H
23	NE2	GLN	505	O	SER	86	2.8-H
24	ND2	ASN	526	O	GLY	87	2.84-H
25	O	LEU	549	NZ	LYS	79	2.91-H
26	ND2	ASN	575	OE1	GLU	83	2.69-H
27	NE2	GLN	578	OE2	GLU	115	2.81-H
28	OE2	GLU	603	NZ	LYS	119	2.69-H
29	OE2	GLU	605	NZ	LYS	119	2.7-H
30	NE	ARG	606	OE2	GLU	115	2.81-H
31	NH1	ARG	606	O	GLU	83	3.11-H
32	NH2	ARG	606	O	GLU	83	2.84-H

<table>
<tr><td>33</td><td>OE2</td><td>GLU</td><td>42</td><td>NH1</td><td>ARG</td><td>212</td><td>2.75-salt bridge</td></tr>
<tr><td>34</td><td>NH1</td><td>ARG</td><td>234</td><td>OD1</td><td>ASP</td><td>202</td><td>2.72-salt bridge</td></tr>
<tr><td>35</td><td>OE1</td><td>GLU</td><td>270</td><td>NZ</td><td>LYS</td><td>318</td><td>2.65-salt bridge</td></tr>
<tr><td>36</td><td>NH2</td><td>ARG</td><td>322</td><td>OD2</td><td>ASP</td><td>313</td><td>2.74-salt bridge</td></tr>
<tr><td>37</td><td>OE2</td><td>GLU</td><td>603</td><td>NZ</td><td>LYS</td><td>119</td><td>2.69-salt bridge</td></tr>
<tr><td>38</td><td>OE1</td><td>GLU</td><td>605</td><td>NZ</td><td>LYS</td><td>119</td><td>2.7-salt bridge</td></tr>
<tr><td>39</td><td>NE</td><td>ARG</td><td>606</td><td>OE1</td><td>GLU</td><td>115</td><td>2.81-salt bridge</td></tr>
<tr><td colspan="4">TLR4 Non-Bonded Contacts: VAL32, MET41, GLU42, PHE63, ASP84, SER86, ARG87, THR110, GLU135, HIS159, LEU161, LYS186, LEU212, ARG234, PHE263, ARG264, ASN265, GLU266, GLY267, GLU270, ARG289, TYR292, ASP294, TYR296, VAL316, SER317, THR319, GLU321, ARG322, ASN339, LYS341, SER360, LYS362, ASN383, PHE408, LYS477, PHE500, ASP502, SER504, GLN505, ASN526, SER528, HIS529, VAL548, LEU549, ASP550, SER552, LEU553, PHE573, ASN575, THR577, GLN578, VAL602, GLU603, GLU605, and ARG606</td><td colspan="4">Vaccine Non-Bonded Contacts: THR126, ARG212, THR213, GLN209, LEU210, PHE257, CYS289, ARG207, ASP202, GLY199, ASP203, TYR263, LEU306, VAL193, GLY295, TRP142, GLY293, THR294, VAL321, PHE316, LYS318, GLY198, TYR314, ASP313, GLU196, GLY312, LYS145, ALA147, VAL195, PRO311, ALA148, GLY151, SER152, LYS153, LEU90, GLY89, GLY87, SER86, LYS79, GLU83, ARG82, ILE84, GLU115, VAL80, and LYS119</td></tr>
</table>

Table S9. Fraction of hydrogen bonds at the designed vaccine construct–TLRs interface during the 100ns simulation timescale (with occupancy ≥ 2%)

Modeled Vaccine-TLR2 complex			
**TLR2 resdiue@atom: Vaccine construct residue@atom**	**No. of frames**	**Frac.**	**Avg**
GLU77@OE2: VAL168@N	5970	54.3	2.88
ARG544@NH2: GLU322@O	2586	23.5	2.87
LYS124@NZ: ALA57@OE1	2391	21.7	2.81
LYS282@NZ: ALA69@O	1273	11.6	2.82
GLN531@NE2: GLY293@OG1	802	7.29	2.89
GLN528@O: GLY293@OG1	756	6.87	2.86
GLU77@OE2: VAL168@OG	747	6.79	2.69
LYS525@O: VAL317@NZ	587	5.34	2.81
GLN528@NE2: GLY295@O	355	3.23	2.87
SER533@OG: GLU322@O	227	2.06	2.91
**Modeled Vaccine-TLR3 complex**			
**TLR3 resdiue@atom: Vaccine construct residue@atom**	**No. of frames**	**Frac.**	**Avg**
ASN36@ND2: GLY151@OG	4159	37.8	2.88
LYS81@NZ: GLY310@O	2711	24.6	2.83
GLU512@OE1: VAL168@OG	2425	22	2.71
PRO230@NH2: SER201@OD1	2238	20.3	2.83
LYS81@NZ:PRO311@O	2132	19.4	2.83
LYS81@NZ: GLY312@OD1	1409	12.8	2.8
LYS309@NZ: LEU67@O	1342	12.2	2.82
HIS544@ND1: VAL168@OG	1218	11.1	2.85
ASP60@OD2: GLY151@OG	1134	10.3	2.68
GLU154@OE1: LEU144@NZ	1108	10.1	2.79
TYR262@OH: VAL97@O	844	7.67	2.78
HIS644@NE2: LYS14@OE1	783	7.12	2.79
ARG310@NH1: ALA123@O	754	6.85	2.83
GLU154@OE2: LEU144@NZ	539	4.9	2.84
LYS6@NZ: PHE32@OE1	408	3.71	2.8
GLU413@OE2:PRO250@N	289	2.63	2.86
**Modeled Vaccine-TLR4 complex**			
**TLR4 resdiue@atom: Vaccine construct residue@atom**	**No. of frames**	**Frac.**	**Avg**
ASN313@ND2: CYS194@O	7296	66.3	2.85
ASN549@ND2: ARG82@OE2	4825	43.9	2.82
GLU16@OE2: LEU211@NH1(63%)	4595	41.8	2.81
SER291@OG: VAL195@OE2	3175	28.9	2.71
ARG296@NH2: GLY312@OD2(47%)	2800	25.5	2.82
ARG296@NE: GLY312@OD2	2494	22.7	2.81
GLU16@OE1: LEU211@NE	2379	21.6	2.84
LYS315@NZ: LEU311@O	1984	18	2.83
GLU244@OE1: VAL317@NZ	1874	17	2.81
GLU577@OE2: ALA118@NZ	1519	13.8	2.79
ARG580@NH2: ARG82@O	1438	13.1	2.85
GLU240@OE2: GLY293@N	1192	10.8	2.86
TYR270@OH:ASP313@O	937	8.52	2.7
ARG208@NH1: GLY198@O	691	6.28	2.82
LYS315@NZ: GLY310@O	559	5.08	2.83
LEU523@O: ILE78@NZ	432	3.93	2.88
ARG208@NH1: SER201@OD1	241	2.19	2.8
