# Supplementary material for: An Immunoinformatics Approach to Design a Potent Multi-Epitope Vaccine against Asia-1 Genotype of Crimean–Congo Haemorrhagic Fever Virus Using the Structural Glycoproteins as a Target
Source: Vaccines (Basel). 2022 Dec 27;11(1):61. doi: 10.3390/vaccines11010061 (PMC9867508; doi:10.3390/vaccines11010061)

Supporting Information 2

An Immunoinformatics Approach to Design a Potent Multi-Epitope Vaccine Against Asia-1 Genotype of Crimean-Congo Haemorrhagic Fever Virus Using the Structural Glycoproteins as a Target

Syed Zawar Shah[1†], Basit Jabbar[1], Muhammad Usman Mirza[2], Muhammad Waqas[3,4†], Shahkaar Aziz[5], Sobia Ahsan Halim[4], Amjad Ali[3*], Shazia Rafique[1], Muhammad Idrees[1], Asaad Khalid[6,7], Ashraf N. Abdalla[8], Ajmal Khan[4*], and Ahmed Al-Harrasi[4*]

1. Centre of Excellence in Molecular Biology, University of the Punjab, 53700, Punjab, Pakistan.
2. Department of Chemistry and Biochemistry, University of Windsor, ON, Canada.
3. Department of Biotechnology and Genetic Engineering, Hazara University Mansehra 21120, Mansehra, Pakistan.
4. Natural and Medical Sciences Research Center, University of Nizwa, Birkat-ul-Mouz 616, Nizwa, Oman
5. Institute of Biotechnology and Genetic Engineering, the University of Agriculture Peshawar, Pakistan.
6. Substance Abuse and Toxicology Research Center, Jazan University, P.O. Box: 114, Jazan 45142, Saudi Arabia.
7. Medicinal and Aromatic Plants and Traditional Medicine Research Institute, National Center for Research, P. O. Box 2404, Khartoum, Sudan
8. Department of Pharmacology and Toxicology, College of Pharmacy, Umm Al-Qura University, Makkah 21955, Saudi Arabia.

†These authors have contributed equally to this work.

***** Correspondence: amjad.camb@pu.edu.pk (A.A.); ajmalkhan@unizwa.edu.om (A.K); aharrasi@unizwa.edu.om (A.A-H)

A. Multiple Sequence Alignment of Congo virus Asia-1 genotype genomes retrieved from NCBI with accession numbers AAM48107, AAK52742, BAB84572, AIE16134, ABB30033, AIE16135, ABB30035, ABB30032, AIE16129, AIE1612B8, AIE16130, AIE16131, AIE16133, AIE16132, AAW84284, AAK52743, ABB30029, BA84577, AF338470 and BAB84578

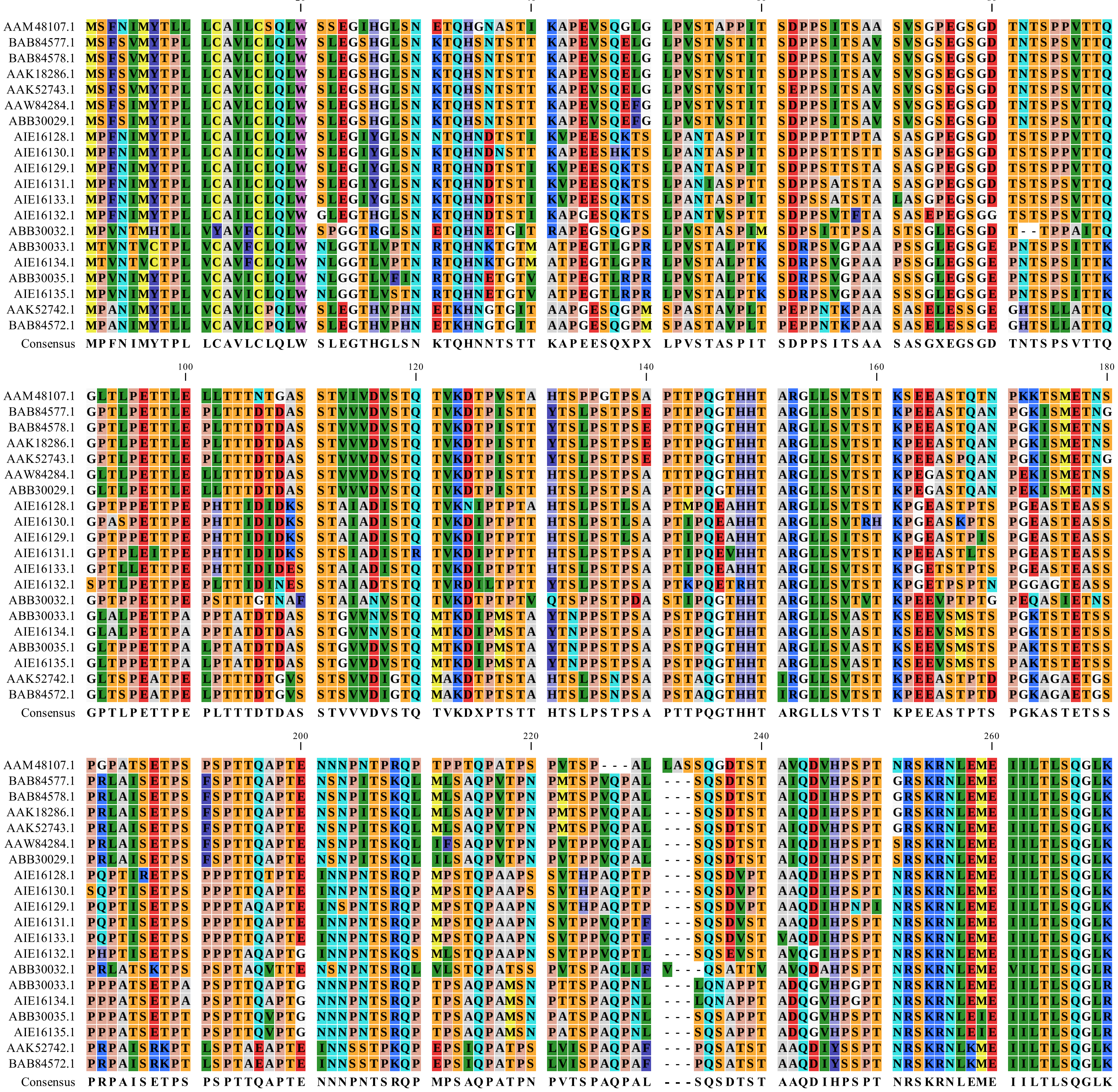

	280		300		320		340		360
AAM48107.1	KYYGKILKLL	DLTLEEDTEG	LLEWCKRNLG	LVCDDSFFQK	RIEEFFLTGE	GHFNEVLQFK	TPSTLSPTEP	ARAGSPTVEP	FKSYFARGLL
BAB84577.1	KYYGKILKLL	DLTLEEDTEG	LLEWCKRNLG	LVCDDNFFQK	RIEEFFLTGK	GHFNEVLQFK	TPSTLSPTGP	ARTGSPTVEP	FKSYFAKGFL
BAB84578.1	KYYGKILKLL	DLTLEEDTEG	LLEWCKRNLG	LVCDDNFFQK	RIEEFFLTGK	GHFNEVLQFK	TPSTLSPTGP	ARTGSPTVEP	FKSYFAKGFL
AAK18286.1	KYYGKILKLL	DLTLEEDTEG	LLEWCKRNLG	LVCDDNFFQK	RIEEFFLTGK	GHFNEVLQFK	TPSTLSPTGP	ARTGSPTVEP	FKSYFAKGFL
AAK52743.1	KYYGKILKLL	DLTLEEDTEG	LLEWCKRNLG	LVCDDNFFQK	RIEEFFLTGK	GHFNEVLQFK	TPSTLSPTGP	ARTGSPTVEP	FKSYFAKGFL
AAW84284.1	KYYGKILKLL	DLTLEEDTEG	LLEWCKRNLG	LVCDADFFQK	RIEEFFLTGE	GHFNEVLQFK	TPSTLSPTGP	ARTGSPTVEP	FKSYFAKGFL
ABB30029.1	KYYGKILKLL	DLTLEEDTEG	LLEWCKRNLG	LVCDDDFFQK	RIEEFFLTGE	GHFNEVLQFK	TPSTLSPTGP	ARTGSPTVEP	FKSYFAKGFL
AIE16128.1	KYYGKVLKLL	DLTLEEDTEG	LLEWCKRNLG	LTCDDNFFQK	RIEEFFLTGE	GHFNEVLQFK	TPSTLSPTEP	ARVESPTVEP	FKSYFAKGFL
AIE16130.1	KYYGKVLKLL	DLTLEEDTEG	LLEWCKRNLG	LTCDDNFFQK	RIEEFFLTGE	GHFNEVLQFK	TPSTLSPTEP	ARVESPTVEP	FKSYFAKGFL
AIE16129.1	KYYGKVLKLL	DLTLEEDTEG	LLESCKRNLG	LTCDDNFFQK	RIEEFFLTGE	GHFNEVLQFK	TPSTLSPTEP	ARVESPTVEP	FKSYFAKGFL
AIE16131.1	KYYGKVLKLL	DLTLEEDTEG	LLEWCKRNLG	LTCDDNFFQK	RIEEFFLTGE	GHFNEVLQFK	TPSTLSPTEP	ARVESPTVEP	FKSYFAKGFL
AIE16133.1	KYYGKVLKLL	DLTLEEDTEG	LLEWCKRNLG	LTCDDNFFQK	RIEEFFLTGE	GHFNEVLQFK	TPSTLSPTEP	ARVESPTVEP	FKSYFAKGFL
AIE16132.1	KYYGKMLKLL	DLTLEEDTEG	LLEWCKRNLG	LTCDDNFFQK	RIEEFFLTGE	GHFNEVLQFK	TPSTPSPTKP	AHVESPTVEP	FKSYFAKGFL
ABB30032.1	KYYGRILKLL	DLTLEEDTEG	LLEWCKRNLG	LTCDDNFFQK	RIEEFFLTGE	GHFNEVLQFK	TPSTLSSTES	ASVGLPTVEP	FKSYFAKGFL
ABB30033.1	KYYGKILKFL	DLTLEEDTEG	LLEWCKRNLG	LTCDDNFFQK	RIEEFFLTGE	GYFNEVLQFK	VPSTLSSTEP	THTGSPTVEP	FKSYFAKGFL
AIE16134.1	KYYGKILKFL	DLTLEEDTEG	LLEWCKRNLG	LTCDDNFFQK	RIEEFFLTGE	GYFNEVLQFK	VPSTLSSTEP	THTGSPTVEP	FKSYFAKGFL
ABB30035.1	KYYGKILKLL	DLTLEEDTEG	LLEWCKRNLG	LTCDDNFFQK	RIEEFFLTGE	GYFNEVLQFK	VPSTLSSTEP	THTRPPTVEP	FKSYFAKGFL
AIE16135.1	KYYGKILKLL	DLTLEEDTEG	LLEWCKRNLG	LTCDDNFFQK	RIEEFFLTGE	GYFNEVLQFK	VPSTLSSTEP	THTRPPTVEP	FKSYFAKGFL
AAK52742.1	KYYGKIQKTL	GLTLEEDTEG	LLEWCKRNLG	LTCDDNFFQK	RIEEFFLTGE	GYFNEVLQFK	TPSTPGSTEP	THAEAPTVKP	FKSYFARGFL
BAB84572.1	KYYGKIQKTL	GLTLEEDTEG	LLEWCKRNLG	LTCDDNFFQK	RIEEFFLTGE	GYFNEVLQFK	TPSTPGSTEP	THTEAPTVKP	FKSYFARGFL
Consensus	KYYGKILKLL	DLTLEEDTEG	LLEWCKRNLG	LTCDDNFFQK	RIEEFFLTGE	GHFNEVLQFK	TPSTLSPTEP	ARTGSPTVEP	FKSYFAKGFL

		380		400		420		440	
AAM48107.1	SMDSGYFSAK	CYPRASNSGL	QLINITQHSA	RITDTPGPKI	TNLKTINCIN	LKASTFKEHR	EIEINVLLPQ	VAVNLSSCHV	LIKSHVCDYS
BAB84577.1	SMDSGYFSAK	CYPRASNSGL	QLINVTQHST	RITDTPGPKI	TNLKTINCIN	LKASVFKEHR	EIEINVLLPQ	VAVNLSSCHV	LIKSHVCDYS
BAB84578.1	SMDSGYFSAK	CYPRASNSGL	QLINVTQHST	RITDTPGPKI	TNLKTINCIN	LKASVFKEHR	EIEINVLLPQ	VAVNLSSCHV	LIKSHVCDYS
AAK18286.1	SMDSGYFSAK	CYPRASNSGL	QLINVTQHST	RITDTPGPKI	TNLKTINCIN	LKASVFKEHR	EIEINVLLPQ	VAVNLSSCHV	LIKSHVCDYS
AAK52743.1	SMDSGYFSAK	CYPRASNSGL	QLINVTQHST	RITDTPGPKI	TNLKTINCIN	LKASVFKEHR	EIEINVLLPQ	VAVNLSSCHV	LIKSHVCDYS
AAW84284.1	SMDSGYFSAK	CYPRASNSGL	QLINVTQHST	RITDTPGPKV	TNLKTINCIN	LKASVFKEHR	EIEINVLLPQ	VAVNLSSCHV	LIKSHVCDYS
ABB30029.1	SMDSGYFSAK	CYPRASNSGL	QLINVTQHST	RITDTPGPKV	TNLKTINCIN	LKASVFKEHR	EIEINVLLPQ	VAVNLSSCHV	LIKSHVCDYS
AIE16128.1	LMDSGYFSAK	CYPKSSNSGL	QLINVTQHLI	RITDTPGPKI	TNLKTINCIN	LKALVFKEHR	EIEINVLLPQ	VAVNLSNCHV	LIKSHVCDYS
AIE16130.1	SMDSGYFSAK	CYPKSSNSGL	QLINVTQHLI	RITDTPGPKI	TNLKTINCIN	LKALVFKEHR	EIEINVLLPQ	VAVNLSNCHV	LIKSHVCDYS
AIE16129.1	SMDSGYFSAK	CYSKSSNSGL	QLINVTQHLI	RITDTPGPKI	TNLKTINCIN	LKALVFKEHR	EIEINVLLPQ	VAVNLSNCHV	LIKSHVCDYS
AIE16131.1	SMDSGYFSAK	CYPKSSNSGL	QLINVTQHLI	RITDTPGPKI	TNLKTINCIN	LKALVFKEHR	EIEINVLLPQ	VAVNLSNCHV	LIKSHVCDYS
AIE16133.1	SMDSGYFSAK	CYPKSSNSGL	QLINVTQHSI	RITDTPGPKI	TSLKTINCIN	LKALVFKEHR	EIEINVLLPQ	VAVNLSNCHV	LIKSHACDYS
AIE16132.1	SMDSGYFSAK	CYPKSSNSGL	QLINITQHSI	RITDTPGPKI	TSLKTINCIN	LKALVFKEHR	EIEINVLLPQ	VAVNLSNCHV	LIKSHVCDYS
ABB30032.1	SIDSGYFSAK	CYPRASNSGL	QLINVTQHSA	RIADTPGPKI	TNLKTINCIN	LKALVFKEHR	EIEINVLLPQ	VAVNLSNCHV	HIKSHVCDYS
ABB30033.1	SMNSGYFSAK	CYPRTSNSGL	QLINVTKHSA	KIADTPGPKI	TNLKTINCIN	LRASVFKEHR	EVEINVLLPQ	VAVNLSNCHA	QIKSHVCDYS
AIE16134.1	SMNSGYFSAK	CYPRTSNSGL	QLINVTKHSA	KIADTPGPKI	TNLKTINCIN	LRASVFKEHR	EVEINVLLPQ	VAVNLSNCHA	QIKSHVCDYS
ABB30035.1	SMNSGYFSAK	CYPRTSNSGL	QLINVTKHSA	KIADTPGPKI	TNLKTINCIN	LRASVFKEHR	EVEINVLLPQ	VAVNLSNCHA	QIKSHVCDYS
AIE16135.1	SMNSGYFSAK	CYPRTSNSGL	QLINVTKHSA	KIADTPGPKI	TNLKTINCIN	LRASVFKEHR	EVEINVLLPQ	VAVNLSNCHA	QIKSHVCDYS
AAK52742.1	TINSGYFSAK	CYSWTSNSGL	QLINVTQHPA	RIADTPGPKI	TNLKTINCIN	LKASVFKEHR	EIEINVLLPQ	VAVNLSNCHV	LIKSHVCDYS
BAB84572.1	TIDSGYFSAK	CYSRTSNSGL	QLINVTQHPA	RIADTPGPKI	TNLKTINCIN	LKASVFKEHR	EIEINVLLPQ	VAVNLSNCHV	LIKSHVCDYS
Consensus	SMDSGYFSAK	CYPRASNSGL	QLINVTQHSA	RITDTPGPKI	TNLKTINCIN	LKASVFKEHR	EIEINVLLPQ	VAVNLSNCHV	LIKSHVCDYS

	460		480		500		520		540
AAM48107.1	LDIDGPVRLP	RIHHEGTFIP	GTYKIVIDKK	NKQNDRCTIV	TNCVIKGREI	RKGQSVLRQY	KTEIKIGKAS	SGSRRLLSEE	SGDDCISRTQ
BAB84577.1	LDIDGPVRLP	RIHHEGTFIP	GTYKIVIDKK	NKQNDRCTLV	TNCVIKGREI	RKGQSVLRQY	RTEIKIGKAS	SGSRRLLSEE	SGDDCISRTQ
BAB84578.1	LDIDGPVRLP	RIHHEGTFIP	GTYKIVIDKK	NKQNDRCTLV	TNCVIKGREI	RKGQSVLRQY	RTEIKIGKAS	SGSRRLLSEE	SGDDCISRTQ
AAK18286.1	LDIDGPVRLP	RIHHEGTFIP	GTYKIVIDKK	NKQNDRCTLV	TNCVIKGREI	RKGQSVLRQY	RTEIKIGKAS	SGSRRLLSEE	SGDDCISRTQ
AAK52743.1	LDIDGPVRLP	RIHHEGTFIP	GTYKIVIDKK	NKQNDRCTLV	TNCVIKGREI	RKGQSVLRQY	RTEIKIGKAS	SGSRRLLSEE	SGDDCISRTQ
AAW84284.1	LDIDGPVRLP	RIHHEGTFIP	GTYKLVIDKK	NKQNDRCTLI	TNCVIKGREI	RKGQSVLRQY	RTEIKIGKAS	SGSRRLLSEE	SGDDCISRTQ
ABB30029.1	LDIDGPVRLP	RIHHEGTFIP	GTYKLVIDKK	NKQNDRCTLI	TNCVIKGREI	RKGQSVLRQY	RTEIKIGKAS	SGSRRLLSEE	SGDDCISRTQ
AIE16128.1	IDIDGPVRLP	RIHHEGTFIP	GTYKIVIDKK	NKQNDRCTLV	TNCVIKGREI	RKGQSVLRQY	RTEIKIGKAS	SGSRRLLSEE	SNDDCLSRTQ
AIE16130.1	LDIDGPVRLP	SIHHEGTFIP	GTYKIVIDKK	NKQNDRCTLV	TNCVIKGREI	RKGQSVLRQY	RTEIKIGKAS	SGSRRLLSEE	PNYDCLSRTQ
AIE16129.1	LDIDGPVRLP	RIHHEGTFIP	GTYKIVIDKN	NKQNDRCTLV	TNCVIKGREI	RKGQSVLRQY	RTEIKIGKAS	SGSRRLLSEE	SNDDCLSRTQ
AIE16131.1	LDIDGPVRLP	RIHHEGTFIP	GTYKIVIDKK	TKQNDRCTLV	TNCVIKGREI	RKGQSVLRQY	RTEIKIGKAS	SGSRRLLSEE	SNDDCLSRTQ
AIE16133.1	LDIDGPVRLP	RIHHEGTFIP	GTYKIVIDKK	NKQNDRCTLV	TNCVIKGREI	RKGQSVLRQY	RTEIKIGKAS	SGSRRLLSEE	SNDDCLSRTQ
AIE16132.1	LDIDGPVRLP	HIHHEGTFIP	GTYKIVIDKK	DKQNDRCTLV	TNCVIKGREI	RKGQSVLRQY	RTEIKIGKAS	SGSRRLLSEE	SSDDCLSRTQ
ABB30032.1	LDIDGTVRLP	RIHHEGTFIP	GTYKIVIDKK	NKQNDRCNLV	TNCVIKGREV	RKGQSVLRQY	RTEIRIGKAS	SGSRRLLSEE	SGEDCISRTQ
ABB30033.1	LATDGMVRLP	RIHHEGTFIP	GTYKIVIDKK	NKQNDRCTLV	TNCVIKGREV	RKGQSALRQY	KTEIRIGKAS	SGSRRLLSEE	SDGDCISRTQ
AIE16134.1	LATDGMVRLP	RIHHEGTFIP	GTYKIVIDKK	NKQNDRCTLV	TNCVIKGREV	RKGQSALRQY	KTEIRIGKAS	SGSRRLLSEE	SDGDCISRTQ
ABB30035.1	LATDGMVRLP	HIHHEGTFIP	GTYKIVIDKK	NKQNDRCTLV	TNCVIKGREV	RKGQSALRQY	KTEIRIGKAS	SGSRRLLSEE	SDGDCISRTQ
AIE16135.1	LATDGMVRLP	HIHHEGTFIP	GTYKIVIDKK	NKQNDRCTLV	TNCVIKGREV	RKGQSALRQY	KTEIRIGKAS	SGSRRLLSEE	SDGDCISRTQ
AAK52742.1	LDIDGTIRLP	RIYHEGTFIP	GTYKIVIDKR	DKQNDRCTLV	TNCVIKGREV	RKGQSALRQY	RTEIRIGKAS	AGSRRLLSEE	SGDDCISRTQ
BAB84572.1	LDIDGTIRLP	RIYHEGTFIP	GTYKIVIDKR	DKQNDRCTLV	TNCVIKGREV	RRGQSALRQY	RTEIRIGKAS	AGSRRLLSEE	SGDDCISRTQ
Consensus	LDIDGPVRLP	RIHHEGTFIP	GTYKIVIDKK	NKQNDRCTLV	TNCVIKGREI	RKGQSVLRQY	RTEIKIGKAS	SGSRRLLSEE	SGDDCISRTQ

```
                      560         580         600         620
AAM48107.1  LLRTETAEIH SDSYGGPGDK ITICNGSTVV DQRLGSELGC YTINRVKSFK LCENSATGKS CEIDSTPVKC RQGFCLKITQ EGRGHVKLSR
BAB84577.1  LLRTETAEIH NDNYGGPGDK ITICNGSTVV DQRLGSELGC YTINRVKSFK LCENSATGKS CEIDSTPVKC RQGFCLKITQ EGRGHVKLSR
BAB84578.1  LLRTETAEIH SDNYGGPGDK ITICNGSTVV DQRLGSELGC YTINRVKSFK LCENSATGKS CEIDSTPVKC RQGFCLKITQ EGRGHVKLSR
AAK18286.1  LLRTETAEIH SDNYGGPGDK ITICNGSTVV DQRLGSELGC YTINRVKSFK LCENSATGKS CEIDSTPVKC RQGFCLKITQ EGRGHVKLSR
AAK52743.1  LLRTETAEIH NDNYGGPGDK ITICNGSTVV DQRLGSELGC YTINRVNSFN LCENTATGKS CEIDSTPVKC RQGFCLKITQ EGRGHVKLSR
AAW84284.1  LSRTETAEIH SDNYGGPGDK ITICNGSTIV DQRLGSELGC YTINRVKSFK LCENSATGKS CEIDSTPIKC RQGFCLKITQ EGRGHVKLSR
ABB30029.1  LSRTETAEIH SDNYGGPGDK ITICNGSTIV DQRLGSELGC YTINRVKSFK LCENSATGKS CEIDSTPIKC RQGFCLKITQ EGRGHVKLSR
AIE16128.1  LLRTENAEIH SDNYGGPGDK ITICNGSTIV DQRLGSELGC YTINRVKSFK LCENSATGKS CEIDSTPVRC RQGFCLKITQ EGRGHVKLSR
AIE16130.1  LLRTENAEIH SDNYGGPGDK ITICNGSTIV DQRLGSELGC YTINRVKSFK LCENSATGKS CEIDSTPVRC RQGFCLKITQ EGRGHVKLSR
AIE16129.1  LLRTENAEIH SDNYGGPGDK ITICNGSTIV DQRLGSELGC YTINRVKSFK LCENSATGKS CEIDSTPVRC RQGFCLKITQ EGRGHVKLSR
AIE16131.1  LLRTENAEIH SDNYGGPGDK ITICNGSTIV DQRLGSELGC YTINRVKSFK LCENSATGKS CEIDSTPVRC RQGFCLKITQ EGKGHVKLSR
AIE16133.1  LLRTENAEIH SDNYGGPGDK ITICNGSTIV DQRLGSELGC YTINRVKSFK LCENSATGKS CEIDSTPVRC RQGFCLKITQ EGRGHVKLSR
AIE16132.1  LLRTENTEIH SDNYGGPGDK ITICNGSTIV DQRLGSELGC YTINRVKSFK LCENSATGKS CEIDSTPVRC RQGFCLKITQ EGRGHVKLSR
ABB30032.1  LLRTETAEIH GDNYGGPGDK ITICNGSTIV DQRLGSELGC YTINRVKSFK LCENSATGKS CEIDNTPVKC KQGFCLKITQ EGRGHVKLSR
ABB30033.1  LLRTETAEIH SDNYGGPGDK ITICNGSTIV DQRLGSELGC YTINRVKSFK LCENSATGKS CEIDSTPVKC RQGFCLKITQ EGRGHVKLSR
AIE16134.1  LLRTETAEIH SDNYGGPGDK ITICNGSTIV DQRLGSELGC YTINRVKSFK LCENSATGKS CEIDSTPVKC RQGFCLKITQ EGRGHVKLSR
ABB30035.1  LLRTETTEIH SDNYGGPGDK ITICNGSTIV DQRLGSELGC YTINRVKSFK LCENSATGKS CEIDSTPVKC RQGFCLKITQ EGRGHVKLSR
AIE16135.1  LLRTETTEIH SDNYGGPGDK ITICNGSTIV DQRLGSELGC YTINRVKSFK LCENSATGKS CEIDSTPVKC RQGFCLKITQ EGRGHVKLSR
AAK52742.1  LLRTETAEVH GDNYGGPGDK ITICNGSTIV DQRLGSELGC YTINRVKSFK LCENSGTGKS CKIDKHPVKC RQGFCLKITQ EGRGHVKLSR
BAB84572.1  LLRTETAEVH GDNYGGPGDK ITICNGSTIV DQRLGSELGC YTINRVKSFK LCENSGTGKS CKIDNTPVKC RQGFCLKITQ EGRGHVKLSR
Consensus   LLRTETAEIH SDNYGGPGDK ITICNGSTIV DQRLGSELGC YTINRVKSFK LCENSATGKS CEIDSTPVKC RQGFCLKITQ EGRGHVKLSR

                     640         660         680         700         720
AAM48107.1  GSEVVLDACD SSCEIMIPKG TGDILVDCSG GQQHFLKDNL IDLGCPNIPL LGKMAIYICR MSNHPKTTMV FLFWFSFGYV ITCILCKVIF
BAB84577.1  GSEVVLDACD SSCEIMIPKG TGDILVDCSG GQQHFLKDNL IDLGCPNIPL LGKMAIYICR MSNHPKTTMA FLFWFSFGYV VTCILCKVIF
BAB84578.1  GSEVVLDACD SSCEIMIPKG TGDILVDCSG GQQHFLKDNL IDLGCPNIPL LGKMAIYICR MSNHPKTTMG FLFWFSFGYV VTCILCKVIF
AAK18286.1  GSEVVLDACD SSCEIMIPKG TGDILVDCSG GQQHFLKDNL IDLGCPNIPL LGKMAIYICR MSNHPKTTMG FLFWFSFGYV VTCILCKGIF
AAK52743.1  GSEVVLDACE SSCEIMIPKG TGDILVDCSG GQQHFLKDNL IDLGCPNIPL LGKMAIYICR MSNHPKTTMA FLFWFSFGYV VTCILCKVIF
AAW84284.1  GSEVVLDACD SSCEIMIPKG TGDILVDCSG GQQHFLKDNL IDLGCPNIPL LGKMAIYICR MSNHPKTTMA FLFWFSFGYV ITCILCKVIF
ABB30029.1  GSEVVLDACD SSCEIMIPKG TGDILVDCSG GQQHFLKDNL IDLGCPNIPL LGKMAIYICR MSNHPKTTMA FLFWFSFGYV ITCILCKVIF
AIE16128.1  GSEVVLDACD SSCEIMIPKG TGDILVDCSG GQQHFLKDNL VDLGCPNIPL LGKMAIYICR MSNHPKTTMA FLFWFSFGYV ITCILCKAIF
AIE16130.1  GSEVVLDACD SSCEIMIPKG TGDILVDCSG GQQHFLKDNL VDLGCPNIPL LGKMAIYICR MSNHPKTTMA FLFWFSFGYV ITCILCKVIF
AIE16129.1  GSEVVLDACD SSCEIMIPKG TGDILVDCSG GQQHFLKDNL VDLGCPNIPL LGKMAIYICR MSNHPKTTMA FLFWFSFGYV ITCILCKVIF
AIE16131.1  GSEVVLDACD SSCEIMIPKG TGDILVDCSG GQQHFLKDNL VDLGCPNIPL LGKMAIYICR MSNHPKTTMV FLFWFSFGYV ITCILCKVIF
AIE16133.1  GSEVVLDACD SSCEIMIPKG TGDILVDCSG GQQHFLKDNL VDLGCPNIPL LGKMAIYICR MSNHPKTTMG FLFWFSFGYV ITCILCKVIF
AIE16132.1  GSEVVLDACD SSCEIMIPKG TGDILVDCSG GQQHFLKDNL VDLGCPNIPL LGKMAIYICR MSNHPKTTMG FLFWFSFGYV ITCILCKVIF
ABB30032.1  DSEVVLDACD SSCEIMIPKG TGDILVDCSG GQQHFLKDNL IDLGCPNIPL LGKMAIYICR MSNHPKTTMA FLFWFSFGYV ITCILCKVIF
ABB30033.1  GSEIVLDACD SSCEIMIPKG TGDILVDCSG GQQHFLKDNL IDLGCPNIPL LGRMAIYICR MSNHPKTTMA FLFWFSFGYV ITCILCKVIF
AIE16134.1  GSEIVLDACD SSCEIMIPKG TGDILVDCSG GQQHFLKDNL IDLGCPNIPL LGRMAIYICR MSNHPKTTMA FLFWFSFGYV ITCILCKVIF
ABB30035.1  GSEIVLDACD SNCEIMIPKG TGDILVDCSG GQQHFLKDNL IDLGCPNIPL LGKMAIYICR MSNHPKTTMA FLFWFSFGYV ITCILCKVIF
AIE16135.1  GSEIVLDACD SNCEIMIPKG TGDILVDCSG GQQHFLKDNL IDLGCPNIPL LGKMAIYICR MSNHPKTTMA FLFWFSFGYV ITCILCKVIF
AAK52742.1  GSEVVLDACD SSCEIMIPKG TGDILVDCSG GQQHFLKDNL VDLGCPNIPL LGKMAIYICR MSNHPKTTMA FLFWFSFGYV ITCIFCKIIF
BAB84572.1  GSEVVLDACD SSCEIMIPKG TGDILVDCSG GQQHFLKDNL VDLGCPNIPL LGKMAIYICR MSNHPKTTMA FLFWFSFGYV ITCIFCKIIF
Consensus   GSEVVLDACD SSCEIMIPKG TGDILVDCSG GQQHFLKDNL IDLGCPNIPL LGKMAIYICR MSNHPKTTMA FLFWFSFGYV ITCILCKVIF

                      740         760         780         800
AAM48107.1  YLLIVVGTLG KKFKQYRELK PQTCTICETT PVNAIDAEMH DLNCSYNICP YCASRLTSDG LARHVMQCPK RKEKIEETEL YLNLERIPWV
BAB84577.1  YLLIVVGTLG KKFKQYRELK PQTCTICETT PVNAIDAEMH DLNCSYNICP YCASRLTSDG LARHVMQCPK RKEKIEETEL YLNLERIPWV
BAB84578.1  YLLIVVGTLG KKFKQYRELK PQTCTICETT PVNAIDAEMH DLNCSYNICP YCASRLTSDG LARHVMQCPK RKEKIEETEL YLNLERIPWV
AAK18286.1  YLLIVVGTLG KKFKQYRELK PQTCTICETT PVNAIDAEMH DLNCSYNICP YCASRLTSDG LARHVMQCPK RKEKIEETEL YLNLERIPWV
AAK52743.1  YLLIVVGTLG KKFKQYRELK PQTCTICETT PVNAIDAEMH DLNCSYNICP YCASRPTSDG LARHVMQCPK RKEKIEETAL YLNLERIPWV
AAW84284.1  YLLIVVGTLG KKFKQYRELK PQTCTICETT PVNAIDAEMH DLNCSYNICP YCASRLTSDG LARHVMQCPK RKEKVEETEL YLNLERIPWV
ABB30029.1  YLLIVVGTLG KKFKQYRELK PQTCTICETT PVNAIDAEMH DLNCSYNICP YCASRLTSDG LARHVMQCPK RKEKVEETEL YLNLERIPWV
AIE16128.1  YLLIVVGTLG KKFKQYRELK PQTCTICETN PVNAIDAEMH DLNCSYNICP YCASRLTSDG LARHVMQCPK RKEKIEETEL YLNLERIPWV
AIE16130.1  YLLIVVGTLG KKFKQYRELK PQTCTICETN PVNAIDAEMH DLNCSYNICP YCASRLTSDG LARHVMQCPK RKEKIEETEL YLNLERIPWV
AIE16129.1  YLLVVVGTLG KKFKQYRELK PQTCTICETN PVNAIDAEMH DLNCSYNICP YCASRLTSDG LARHVIQCPK RKEKIEETEL YLNLERIPWV
AIE16131.1  YLLIVVGTLG KKFKQYRELK PQTCTICETN PVNAIDAEMH DLNCSYNICP YCASRLTSDG LARHVMQCPK RKEKIEETEL YLNLERIPWV
AIE16133.1  YLLIVVGTLG KKFKQYRELK PQTCTICETN PVNAIDAEMH DLNCSYNICP YCASRLTSDG LARHVMQCPK RKEKIEETEL YLNLERIPWV
AIE16132.1  YLLIVVGTLG KKFKQYRELK PQTCTICETT PVNAIDAEMH DLNCSYNICP YCASRLTSDG LARHVMQCPK RKEKIEETEL YLNLERIPWI
ABB30032.1  YLLIVVGTLG KKFKQYRELK PQTCTICETT PVNAIDAEMH DLNCSYNICP YCASRLTSDG LARHVMQCPK RKEKIEETEL YLNLERIPWV
ABB30033.1  YLLIVAGTVG KKFKQYRELR PQTCTICETT PVNAIDAEMH DLNCSYNICP YCASRLTSDG LARHVMQCPK RKEKIEETEL YLNLERIPWV
AIE16134.1  YLLIVAGTVG KKFKQYRELR PQTCTICETT PVNAIDAEMH DLNCSYNICP YCASRLTSDG LARHVMQCPK RKEKIEETEL YLNLERIPWV
ABB30035.1  YLLIVAGTVG KKFKQYRELR PQTCTICETI PVNAIDAEMH DLNCSYNICP YCASRLTSDG LARHVMQCPK RKEKVEETEL YLNLERIPWV
AIE16135.1  YLLIVAGTVG KKFKQYRELR PQTCTICETI PVNAIDAEMH DLNCSYNICP YCASRLTSDG LARHVMQCPK RKEKVEETEL YLNLERIPWV
AAK52742.1  YLLIVVGTLG KKFKQYRELK PQTCTICETT PTNAIDAEMH DLKCSYNICP YCASRLTSDG LARHVTQCPK RKEKVEETEL YLNLERIPWI
BAB84572.1  YLLIVVGTLG KKFKQYRELK PQTCTICETT PTNAIDAEMH DLKCSYNICP YCASRLTSDG LARHVTQCPK RKEKVEETEL YLNLERIPWI
Consensus   YLLIVVGTLG KKFKQYRELK PQTCTICETT PVNAIDAEMH DLNCSYNICP YCASRLTSDG LARHVMQCPK RKEKIEETEL YLNLERIPWV
```

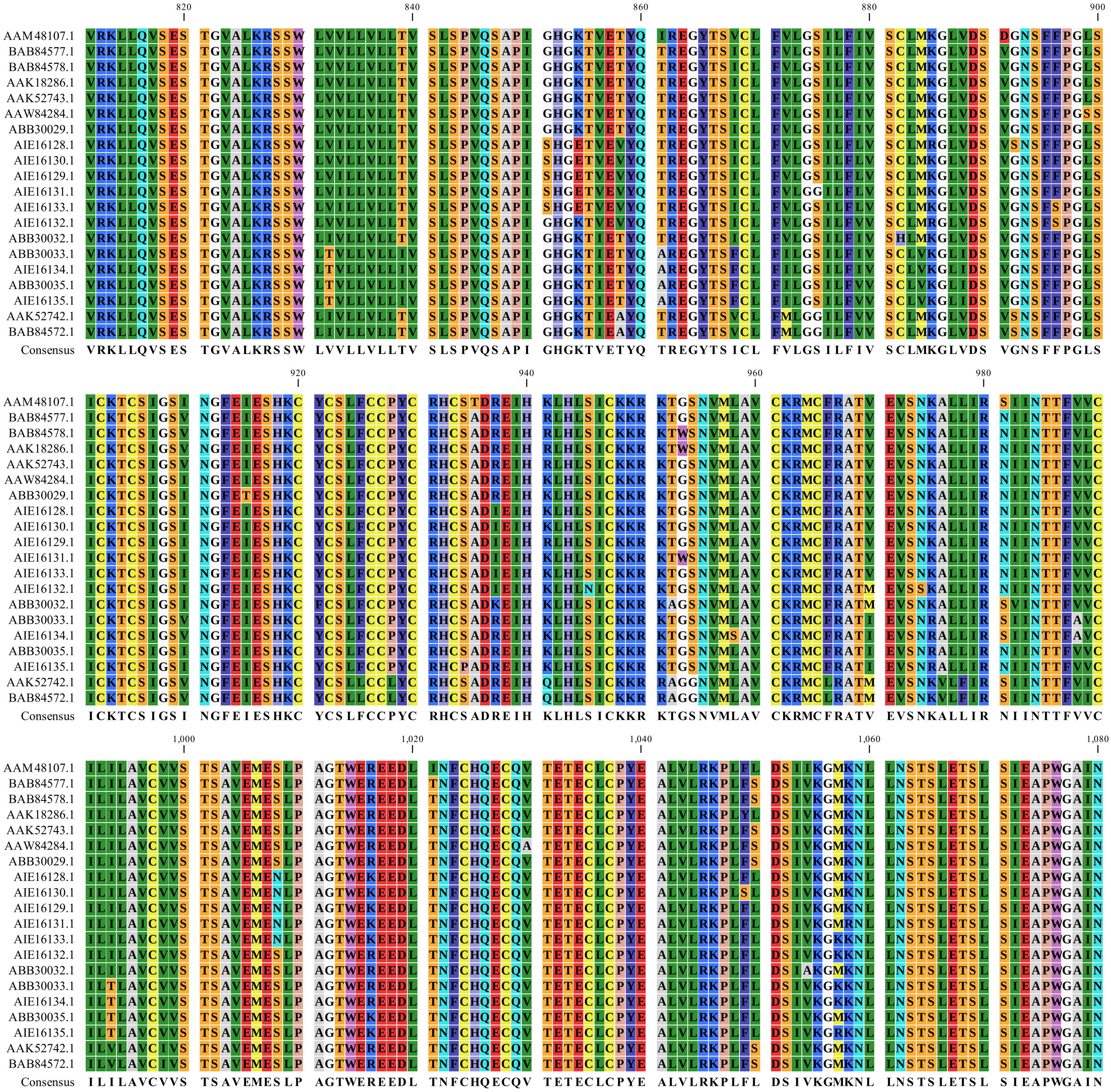
820
840
860
880
900
920
940
960
980
1,000
1,020
1,040
1,060
1,080
AAM48107.1
BAB84577.1
BAB84578.1
AAK18286.1
AAK52743.1
AAW84284.1
ABB30029.1
AIE16128.1
AIE16130.1
AIE16129.1
AIE16131.1
AIE16133.1
AIE16132.1
ABB30032.1
ABB30033.1
AIE16134.1
ABB30035.1
AIE16135.1
AAK52742.1
BAB84572.1
Consensus VRKLLQVSES TGVALKRSSW LVVLLVLLTV SLSPVQSAPI GHGKTVETYQ TREGYTSICL FVLGSILFIV SCLMKGLVDS VGNSFFPGLS
Consensus ICKTCSIGSI NGFEIESHKC YCSLFCCPYC RHCSADREIH KLHLSICKKR KTGSNVMLAV CKRMCFRATV EVSNKALLIR NIINTTFVVC
Consensus ILILAVCVVS TSAVEMESLP AGTWEREEDL TNFCHQECQV TETECLCPYE ALVLRKPLFL DSIVKGMKNL LNSTSLETSL SIEAPWGAIN

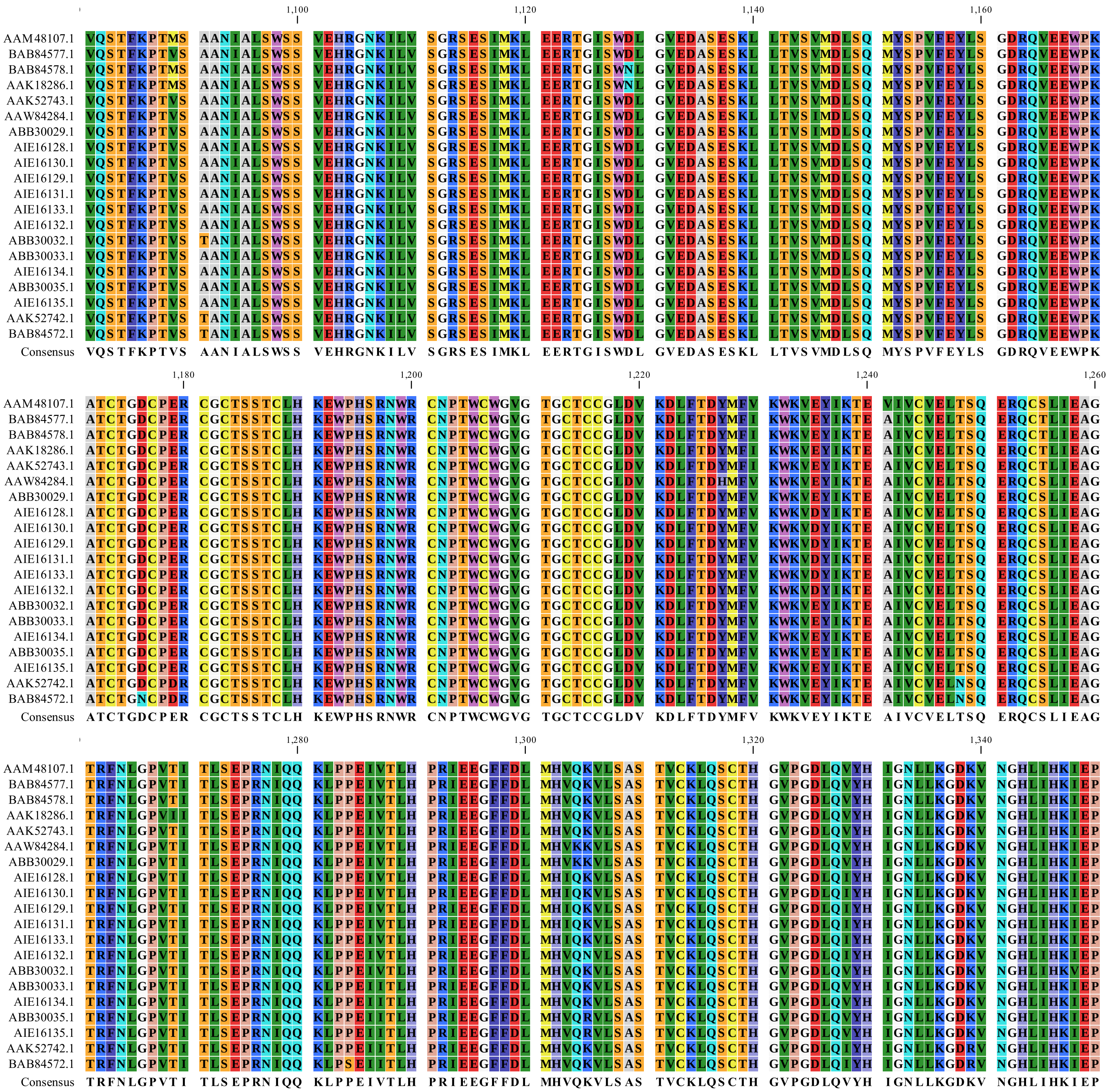
1,100
1,120
1,140
1,160
AAM48107.1
BAB84577.1
BAB84578.1
AAK18286.1
AAK52743.1
AAW84284.1
ABB30029.1
AIE16128.1
AIE16130.1
AIE16129.1
AIE16131.1
AIE16133.1
AIE16132.1
ABB30032.1
ABB30033.1
AIE16134.1
ABB30035.1
AIE16135.1
AAK52742.1
BAB84572.1
Consensus VQSTFKPTVS AANIALSWSS VEHRGNKILV SGRSESIMKL EERTGISWDL GVEDASESKL LTVSVMDLSQ MYSPVFEYLS GDRQVEEWPK
1,180
1,200
1,220
1,240
1,260
Consensus ATCTGDCPER CGCTSSTCLH KEWPHSRNWR CNPTWCWGVG TGCTCCGLDV KDLFTDYMFV KWKVEYIKTE AIVCVELTSQ ERQCSLIEAG
1,280
1,300
1,320
1,340
Consensus TRFNLGPVTI TLSEPRNIQQ KLPPEIVTLH PRIEEGFFDL MHVQKVLSAS TVCKLQSCTH GVPGDLQVYH IGNLLKGDKV NGHLIHKIEP

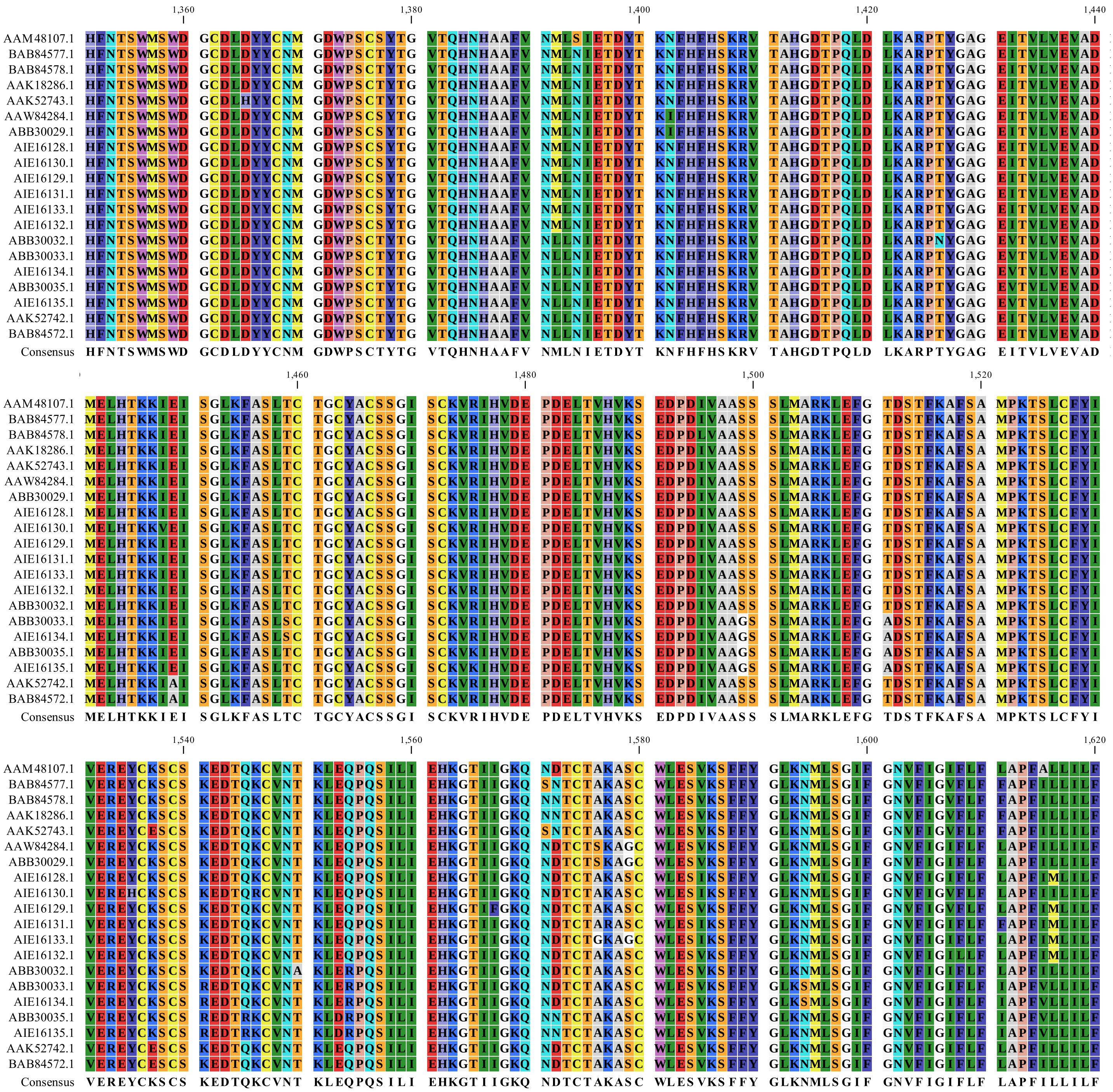

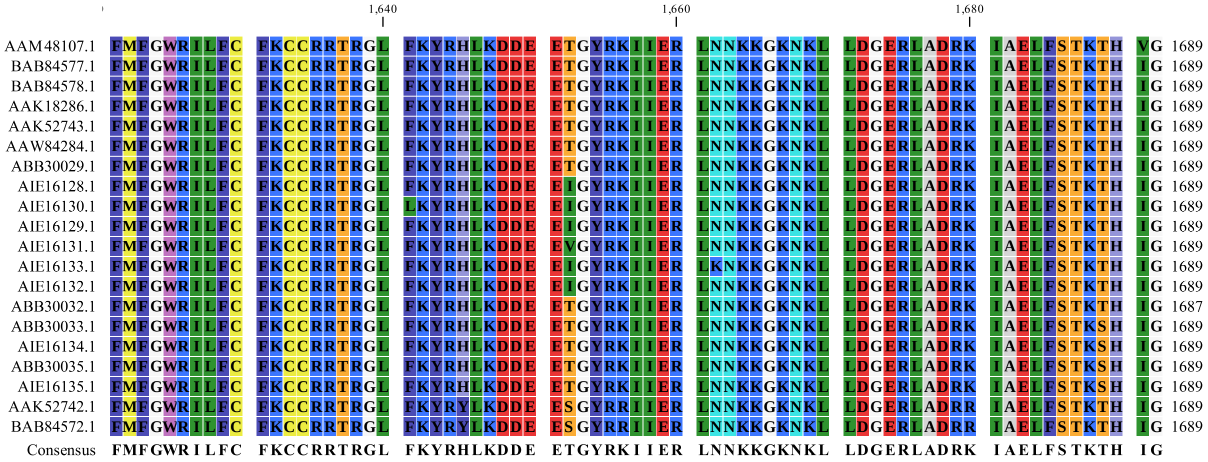
1,640
1,660
1,680
AAM48107.1 FMFGWRILFC FKCCRRTRGL FKYRHLKDDE ETGYRKIIER LNNKKGKNKL LDGERLADRK IAELFSTKTH VG 1689
BAB84577.1 FMFGWRILFC FKCCRRTRGL FKYRHLKDDE ETGYRKIIER LNNKKGKNKL LDGERLADRK IAELFSTKTH IG 1689
BAB84578.1 FMFGWRILFC FKCCRRTRGL FKYRHLKDDE ETGYRKIIER LNNKKGKNKL LDGERLADRK IAELFSTKTH IG 1689
AAK18286.1 FMFGWRILFC FKCCRRTRGL FKYRHLKDDE ETGYRKIIER LNNKKGKNKL LDGERLADRK IAELFSTKTH IG 1689
AAK52743.1 FMFGWRILFC FKCCRRTRGL FKYRHLKDDE ETGYRKIIER LNNKKGKNKL LDGERLADRK IAELFSTKTH IG 1689
AAW84284.1 FMFGWRILFC FKCCRRTRGL FKYRHLKDDE ETGYRKIIER LNNKKGKNKL LDGERLADRK IAELFSTKTH IG 1689
ABB30029.1 FMFGWRILFC FKCCRRTRGL FKYRHLKDDE ETGYRKIIER LNNKKGKNKL LDGERLADRK IAELFSTKTH IG 1689
AIE16128.1 FMFGWRILFC FKCCRRTRGL FKYRHLKDDE EIGYRKIIER LNNKKGKNKL LDGERLADRK IAELFSTKTH IG 1689
AIE16130.1 FMFGWRILFC FKCCRRTRGL LKYRHLKDDE EIGYRKIIER LNNKKGKNKL LDGERLADRK IAELFSTKTH IG 1689
AIE16129.1 FMFGWRILFC FKCCRRTRGL FKYRHLKDDE EIGYRKIIER LNNKKGKNKL LDGERLADRK IAELFSTKTH IG 1689
AIE16131.1 FMFGWRILFC FKCCRRTRGL FKYRHLKDDE EVGYRKIIER LNNKKGKNKL LDGERLADRK IAELFSTKTH IG 1689
AIE16133.1 FMFGWRILFC FKCCRRTRGL FKYRHLKDDE EIGYRKIIER LKNKKGKNKL LDGERLADRK IAELFSTKTH IG 1689
AIE16132.1 FMFGWRILFC FKCCRRTRGL FKYRHLKDDE EIGYRKIIER LNNKKGKNKL LDGERLADRK IAELFSTKTH IG 1689
ABB30032.1 FMFGWRILFC FKCCRRTRGL FKYRHLKDDE ETGYRKIIER LNNKKGKNKL LDGERLADRK IAELFSTKTH IG 1687
ABB30033.1 FMFGWRILFC FKCCRRTRGL FKYRHLKDDE ETGYRKIIER LNNKKGKNKL LDGERLADRK IAELFSTKSH IG 1689
AIE16134.1 FMFGWRILFC FKCCRRTRGL FKYRHLKDDE ETGYRKIIER LNNKKGKNKL LDGERLADRK IAELFSTKSH IG 1689
ABB30035.1 FMFGWRILFC FKCCRRTRGL FKYRHLKDDE ETGYRKIIER LNNKKGKNKL LDGERLADRK IAELFSTKSH IG 1689
AIE16135.1 FMFGWRILFC FKCCRRTRGL FKYRHLKDDE ETGYRKIIER LNNKKGKNKL LDGERLADRK IAELFSTKSH IG 1689
AAK52742.1 FMFGWRILFC FKCCRRTRGL FKYRYLKDDE ESGYRRIIER LNNKKGKNKL LDGERLADRR IAELFSTKTH IG 1689
BAB84572.1 FMFGWRILFC FKCCRRTRGL FKYRYLKDDE ESGYRRIIER LNNKKGKNKL LDGERLADRR IAELFSTKTH IG 1689
Consensus FMFGWRILFC FKCCRRTRGL FKYRHLKDDE ETGYRKIIER LNNKKGKNKL LDGERLADRK IAELFSTKTH IG

B. GP1 protein sequence alignment between the Congo virus Asia-1 genotype retrieved from NCBI with accession numbers AAM48107, AAK52742, BAB84572, AIE16134, ABB30033, AIE16135, ABB30035, ABB30032, AIE16129, AIE1612B8, AIE16130, AIE16131, AIE16133, AIE16132, AAW84284, AAK52743, ABB30029, BA84577, AF338470 and BAB84578

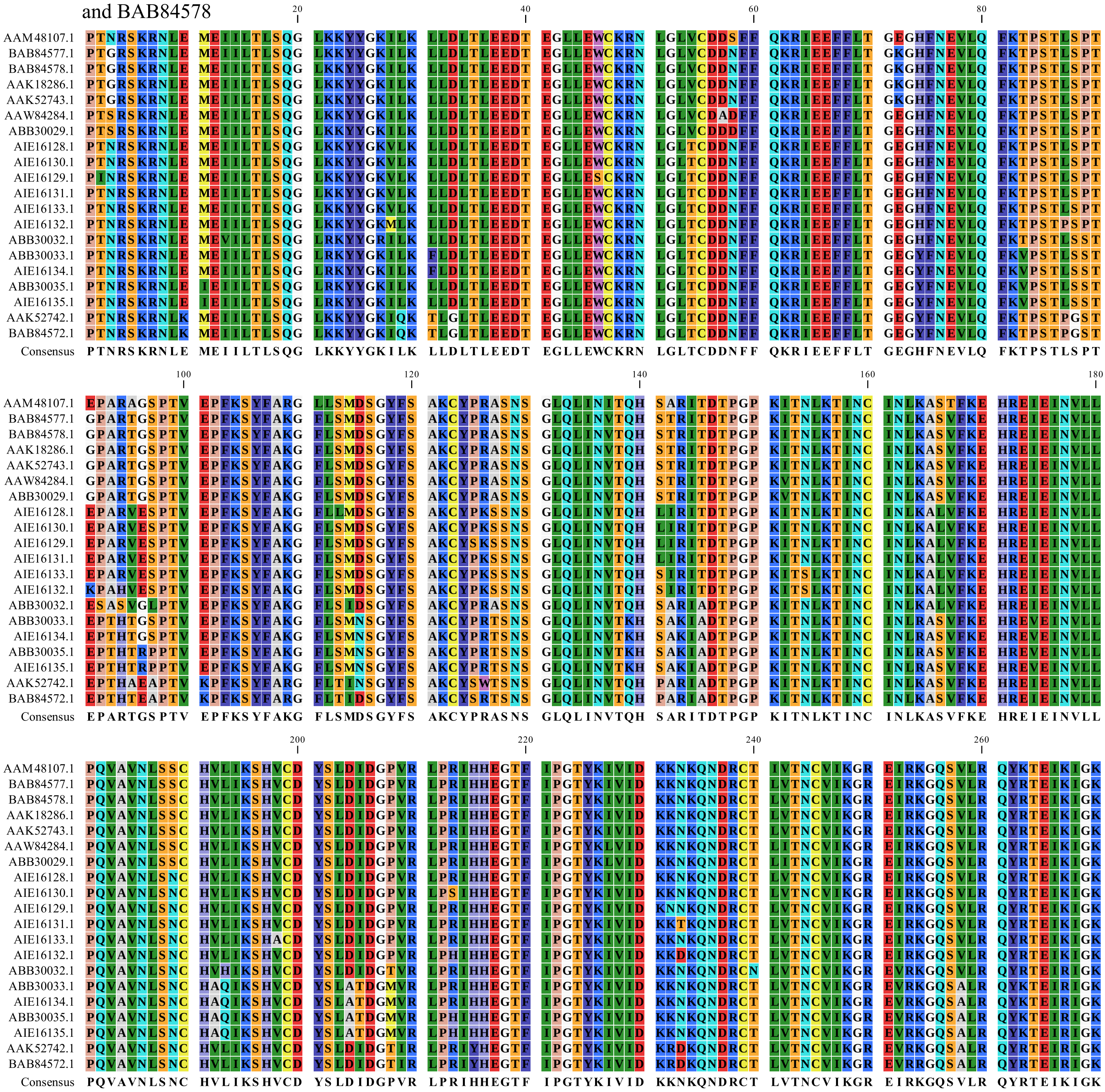

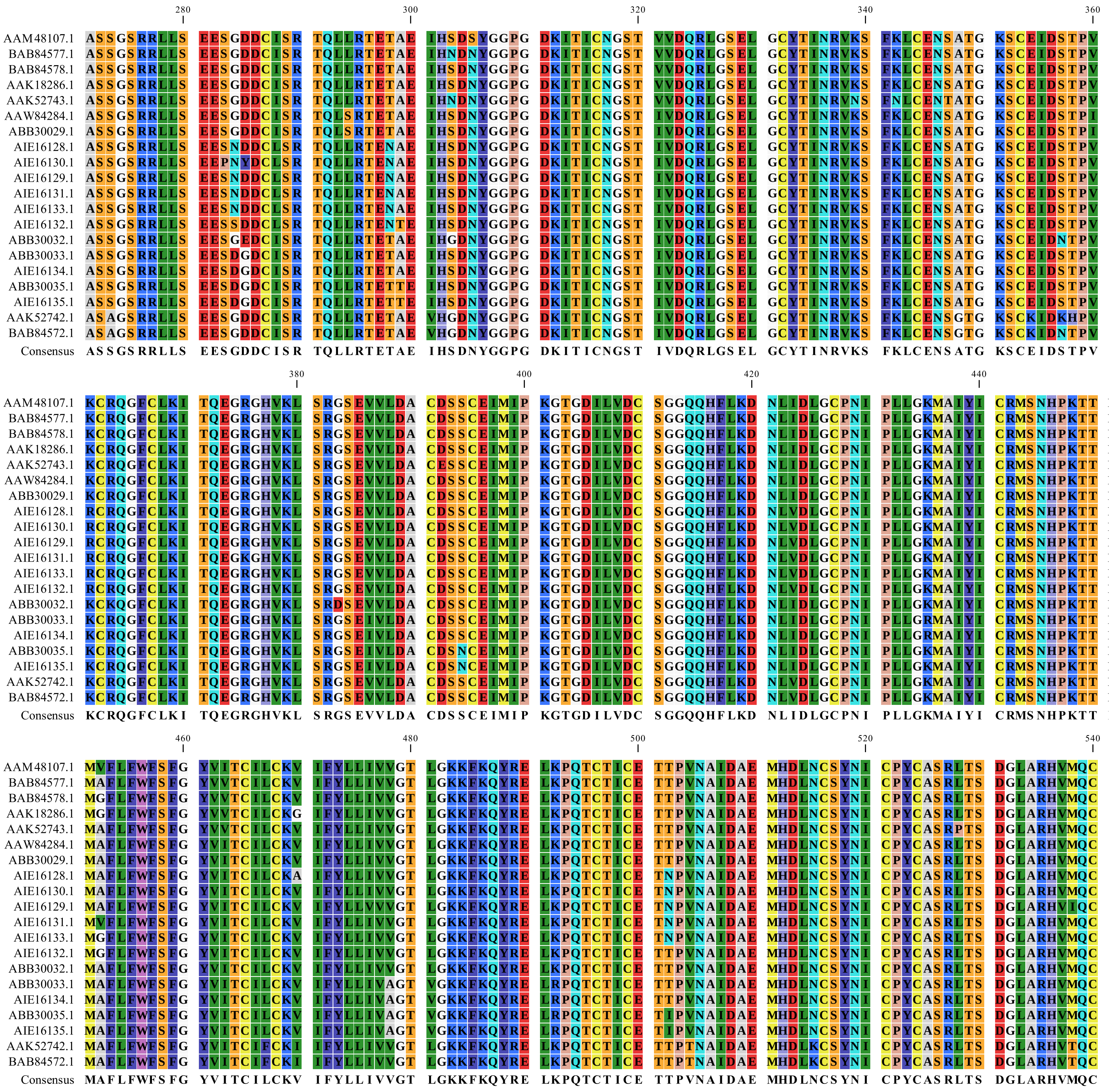

280 300 320 340 360
AAM48107.1 ASSGSRRLLS EESGDDCISR TQLLRTETAE IHSDSYGGPG DKITICNGST VVDQRLGSEL GCYTINRVKS FKLCENSATG KSCEIDSTPV
BAB84577.1 ASSGSRRLLS EESGDDCISR TQLLRTETAE IHNDNYGGPG DKITICNGST VVDQRLGSEL GCYTINRVKS FKLCENSATG KSCEIDSTPV
BAB84578.1 ASSGSRRLLS EESGDDCISR TQLLRTETAE IHSDNYGGPG DKITICNGST VVDQRLGSEL GCYTINRVKS FKLCENSATG KSCEIDSTPV
AAK18286.1 ASSGSRRLLS EESGDDCISR TQLLRTETAE IHSDNYGGPG DKITICNGST VVDQRLGSEL GCYTINRVKS FKLCENSATG KSCEIDSTPV
AAK52743.1 ASSGSRRLLS EESGDDCISR TQLLRTETAE IHNDNYGGPG DKITICNGST VVDQRLGSEL GCYTINRVNS FNLCENTATG KSCEIDSTPV
AAW84284.1 ASSGSRRLLS EESGDDCISR TQLSRTETAE IHSDNYGGPG DKITICNGST IVDQRLGSEL GCYTINRVKS FKLCENSATG KSCEIDSTPI
ABB30029.1 ASSGSRRLLS EESGDDCISR TQLSRTETAE IHSDNYGGPG DKITICNGST IVDQRLGSEL GCYTINRVKS FKLCENSATG KSCEIDSTPI
AIE16128.1 ASSGSRRLLS EESNDDCLSR TQLLRTENAE IHSDNYGGPG DKITICNGST IVDQRLGSEL GCYTINRVKS FKLCENSATG KSCEIDSTPV
AIE16130.1 ASSGSRRLLS EEPNYDCLSR TQLLRTENAE IHSDNYGGPG DKITICNGST IVDQRLGSEL GCYTINRVKS FKLCENSATG KSCEIDSTPV
AIE16129.1 ASSGSRRLLS EESNDDCLSR TQLLRTENAE IHSDNYGGPG DKITICNGST IVDQRLGSEL GCYTINRVKS FKLCENSATG KSCEIDSTPV
AIE16131.1 ASSGSRRLLS EESNDDCLSR TQLLRTENAE IHSDNYGGPG DKITICNGST IVDQRLGSEL GCYTINRVKS FKLCENSATG KSCEIDSTPV
AIE16133.1 ASSGSRRLLS EESNDDCLSR TQLLRTENAE IHSDNYGGPG DKITICNGST IVDQRLGSEL GCYTINRVKS FKLCENSATG KSCEIDSTPV
AIE16132.1 ASSGSRRLLS EESSDDCLSR TQLLRTENTE IHSDNYGGPG DKITICNGST IVDQRLGSEL GCYTINRVKS FKLCENSATG KSCEIDSTPV
ABB30032.1 ASSGSRRLLS EESGEDCISR TQLLRTETAE IHGDNYGGPG DKITICNGST IVDQRLGSEL GCYTINRVKS FKLCENSATG KSCEIDNTPV
ABB30033.1 ASSGSRRLLS EESDGDCISR TQLLRTETAE IHSDNYGGPG DKITICNGST IVDQRLGSEL GCYTINRVKS FKLCENSATG KSCEIDSTPV
AIE16134.1 ASSGSRRLLS EESDGDCISR TQLLRTETAE IHSDNYGGPG DKITICNGST IVDQRLGSEL GCYTINRVKS FKLCENSATG KSCEIDSTPV
ABB30035.1 ASSGSRRLLS EESDGDCISR TQLLRTETTE IHSDNYGGPG DKITICNGST IVDQRLGSEL GCYTINRVKS FKLCENSATG KSCEIDSTPV
AIE16135.1 ASSGSRRLLS EESDGDCISR TQLLRTETTE IHSDNYGGPG DKITICNGST IVDQRLGSEL GCYTINRVKS FKLCENSATG KSCEIDSTPV
AAK52742.1 ASAGSRRLLS EESGDDCISR TQLLRTETAE VHGDNYGGPG DKITICNGST IVDQRLGSEL GCYTINRVKS FKLCENSGTG KSCKIDKHPV
BAB84572.1 ASAGSRRLLS EESGDDCISR TQLLRTETAE VHGDNYGGPG DKITICNGST IVDQRLGSEL GCYTINRVKS FKLCENSGTG KSCKIDNTPV
Consensus ASSGSRRLLS EESGDDCISR TQLLRTETAE IHSDNYGGPG DKITICNGST IVDQRLGSEL GCYTINRVKS FKLCENSATG KSCEIDSTPV
380 400 420 440
AAM48107.1 KCRQGFCLKI TQEGRGHVKL SRGSEVVLDA CDSSCEIMIP KGTGDILVDC SGGQQHFLKD NLIDLGCPNI PLLGKMAIYI CRMSNHPKTT
BAB84577.1 KCRQGFCLKI TQEGRGHVKL SRGSEVVLDA CDSSCEIMIP KGTGDILVDC SGGQQHFLKD NLIDLGCPNI PLLGKMAIYI CRMSNHPKTT
BAB84578.1 KCRQGFCLKI TQEGRGHVKL SRGSEVVLDA CDSSCEIMIP KGTGDILVDC SGGQQHFLKD NLIDLGCPNI PLLGKMAIYI CRMSNHPKTT
AAK18286.1 KCRQGFCLKI TQEGRGHVKL SRGSEVVLDA CDSSCEIMIP KGTGDILVDC SGGQQHFLKD NLIDLGCPNI PLLGKMAIYI CRMSNHPKTT
AAK52743.1 KCRQGFCLKI TQEGRGHVKL SRGSEVVLDA CESSCEIMIP KGTGDILVDC SGGQQHFLKD NLIDLGCPNI PLLGKMAIYI CRMSNHPKTT
AAW84284.1 KCRQGFCLKI TQEGRGHVKL SRGSEVVLDA CDSSCEIMIP KGTGDILVDC SGGQQHFLKD NLIDLGCPNI PLLGKMAIYI CRMSNHPKTT
ABB30029.1 KCRQGFCLKI TQEGRGHVKL SRGSEVVLDA CDSSCEIMIP KGTGDILVDC SGGQQHFLKD NLIDLGCPNI PLLGKMAIYI CRMSNHPKTT
AIE16128.1 RCRQGFCLKI TQEGRGHVKL SRGSEVVLDA CDSSCEIMIP KGTGDILVDC SGGQQHFLKD NLVDLGCPNI PLLGKMAIYI CRMSNHPKTT
AIE16130.1 RCRQGFCLKI TQEGRGHVKL SRGSEVVLDA CDSSCEIMIP KGTGDILVDC SGGQQHFLKD NLVDLGCPNI PLLGKMAIYI CRMSNHPKTT
AIE16129.1 RCRQGFCLKI TQEGRGHVKL SRGSEVVLDA CDSSCEIMIP KGTGDILVDC SGGQQHFLKD NLVDLGCPNI PLLGKMAIYI CRMSNHPKTT
AIE16131.1 RCRQGFCLKI TQEGKGHVKL SRGSEVVLDA CDSSCEIMIP KGTGDILVDC SGGQQHFLKD NLVDLGCPNI PLLGKMAIYI CRMSNHPKTT
AIE16133.1 RCRQGFCLKI TQEGRGHVKL SRGSEVVLDA CDSSCEIMIP KGTGDILVDC SGGQQHFLKD NLVDLGCPNI PLLGKMAIYI CRMSNHPKTT
AIE16132.1 RCRQGFCLKI TQEGRGHVKL SRGSEVVLDA CDSSCEIMIP KGTGDILVDC SGGQQHFLKD NLVDLGCPNI PLLGKMAIYI CRMSNHPKTT
ABB30032.1 KCKQGFCLKI TQEGRGHVKL SRDSEVVLDA CDSSCEIMIP KGTGDILVDC SGGQQHFLKD NLIDLGCPNI PLLGKMAIYI CRMSNHPKTT
ABB30033.1 KCRQGFCLKI TQEGRGHVKL SRGSEIVLDA CDSSCEIMIP KGTGDILVDC SGGQQHFLKD NLIDLGCPNI PLLGRMAIYI CRMSNHPKTT
AIE16134.1 KCRQGFCLKI TQEGRGHVKL SRGSEIVLDA CDSSCEIMIP KGTGDILVDC SGGQQHFLKD NLIDLGCPNI PLLGRMAIYI CRMSNHPKTT
ABB30035.1 KCRQGFCLKI TQEGRGHVKL SRGSEIVLDA CDSNCEIMIP KGTGDILVDC SGGQQHFLKD NLIDLGCPNI PLLGKMAIYI CRMSNHPKTT
AIE16135.1 KCRQGFCLKI TQEGRGHVKL SRGSEIVLDA CDSNCEIMIP KGTGDILVDC SGGQQHFLKD NLIDLGCPNI PLLGKMAIYI CRMSNHPKTT
AAK52742.1 KCRQGFCLKI TQEGRGHVKL SRGSEVVLDA CDSSCEIMIP KGTGDILVDC SGGQQHFLKD NLVDLGCPNI PLLGKMAIYI CRMSNHPKTT
BAB84572.1 KCRQGFCLKI TQEGRGHVKL SRGSEVVLDA CDSSCEIMIP KGTGDILVDC SGGQQHFLKD NLVDLGCPNI PLLGKMAIYI CRMSNHPKTT
Consensus KCRQGFCLKI TQEGRGHVKL SRGSEVVLDA CDSSCEIMIP KGTGDILVDC SGGQQHFLKD NLIDLGCPNI PLLGKMAIYI CRMSNHPKTT
460 480 500 520 540
AAM48107.1 MVFLFWFSFG YVITCILCKV IFYLLIVVGT LGKKFKQYRE LKPQTCTICE TTPVNAIDAE MHDLNCSYNI CPYCASRLTS DGLARHVMQC
BAB84577.1 MAFLFWFSFG YVVTCILCKV IFYLLIVVGT LGKKFKQYRE LKPQTCTICE TTPVNAIDAE MHDLNCSYNI CPYCASRLTS DGLARHVMQC
BAB84578.1 MGFLFWFSFG YVVTCILCKV IFYLLIVVGT LGKKFKQYRE LKPQTCTICE TTPVNAIDAE MHDLNCSYNI CPYCASRLTS DGLARHVMQC
AAK18286.1 MGFLFWFSFG YVVTCILCKG IFYLLIVVGT LGKKFKQYRE LKPQTCTICE TTPVNAIDAE MHDLNCSYNI CPYCASRLTS DGLARHVMQC
AAK52743.1 MAFLFWFSFG YVVTCILCKV IFYLLIVVGT LGKKFKQYRE LKPQTCTICE TTPVNAIDAE MHDLNCSYNI CPYCASRPTS DGLARHVMQC
AAW84284.1 MAFLFWFSFG YVITCILCKV IFYLLIVVGT LGKKFKQYRE LKPQTCTICE TTPVNAIDAE MHDLNCSYNI CPYCASRLTS DGLARHVMQC
ABB30029.1 MAFLFWFSFG YVITCILCKV IFYLLIVVGT LGKKFKQYRE LKPQTCTICE TTPVNAIDAE MHDLNCSYNI CPYCASRLTS DGLARHVMQC
AIE16128.1 MAFLFWFSFG YVITCILCKA IFYLLIVVGT LGKKFKQYRE LKPQTCTICE TNPVNAIDAE MHDLNCSYNI CPYCASRLTS DGLARHVMQC
AIE16130.1 MAFLFWFSFG YVITCILCKV IFYLLIVVGT LGKKFKQYRE LKPQTCTICE TNPVNAIDAE MHDLNCSYNI CPYCASRLTS DGLARHVMQC
AIE16129.1 MAFLFWFSFG YVITCILCKV IFYLLVVVGT LGKKFKQYRE LKPQTCTICE TNPVNAIDAE MHDLNCSYNI CPYCASRLTS DGLARHVIQC
AIE16131.1 MVFLFWFSFG YVITCILCKV IFYLLIVVGT LGKKFKQYRE LKPQTCTICE TNPVNAIDAE MHDLNCSYNI CPYCASRLTS DGLARHVMQC
AIE16133.1 MGFLFWFSFG YVITCILCKV IFYLLIVVGT LGKKFKQYRE LKPQTCTICE TNPVNAIDAE MHDLNCSYNI CPYCASRLTS DGLARHVMQC
AIE16132.1 MGFLFWFSFG YVITCILCKV IFYLLIVVGT LGKKFKQYRE LKPQTCTICE TTPVNAIDAE MHDLNCSYNI CPYCASRLTS DGLARHVMQC
ABB30032.1 MAFLFWFSFG YVITCILCKV IFYLLIVVGT LGKKFKQYRE LKPQTCTICE TTPVNAIDAE MHDLNCSYNI CPYCASRLTS DGLARHVMQC
ABB30033.1 MAFLFWFSFG YVITCILCKV IFYLLIVAGT VGKKFKQYRE LRPQTCTICE TTPVNAIDAE MHDLNCSYNI CPYCASRLTS DGLARHVMQC
AIE16134.1 MAFLFWFSFG YVITCILCKV IFYLLIVAGT VGKKFKQYRE LRPQTCTICE TTPVNAIDAE MHDLNCSYNI CPYCASRLTS DGLARHVMQC
ABB30035.1 MAFLFWFSFG YVITCILCKV IFYLLIVAGT VGKKFKQYRE LRPQTCTICE TIPVNAIDAE MHDLNCSYNI CPYCASRLTS DGLARHVMQC
AIE16135.1 MAFLFWFSFG YVITCILCKV IFYLLIVAGT VGKKFKQYRE LRPQTCTICE TIPVNAIDAE MHDLNCSYNI CPYCASRLTS DGLARHVMQC
AAK52742.1 MAFLFWFSFG YVITCIFCKI IFYLLIVVGT LGKKFKQYRE LKPQTCTICE TTPTNAIDAE MHDLKCSYNI CPYCASRLTS DGLARHVTQC
BAB84572.1 MAFLFWFSFG YVITCIFCKI IFYLLIVVGT LGKKFKQYRE LKPQTCTICE TTPTNAIDAE MHDLKCSYNI CPYCASRLTS DGLARHVTQC
Consensus MAFLFWFSFG YVITCILCKV IFYLLIVVGT LGKKFKQYRE LKPQTCTICE TTPVNAIDAE MHDLNCSYNI CPYCASRLTS DGLARHVMQC

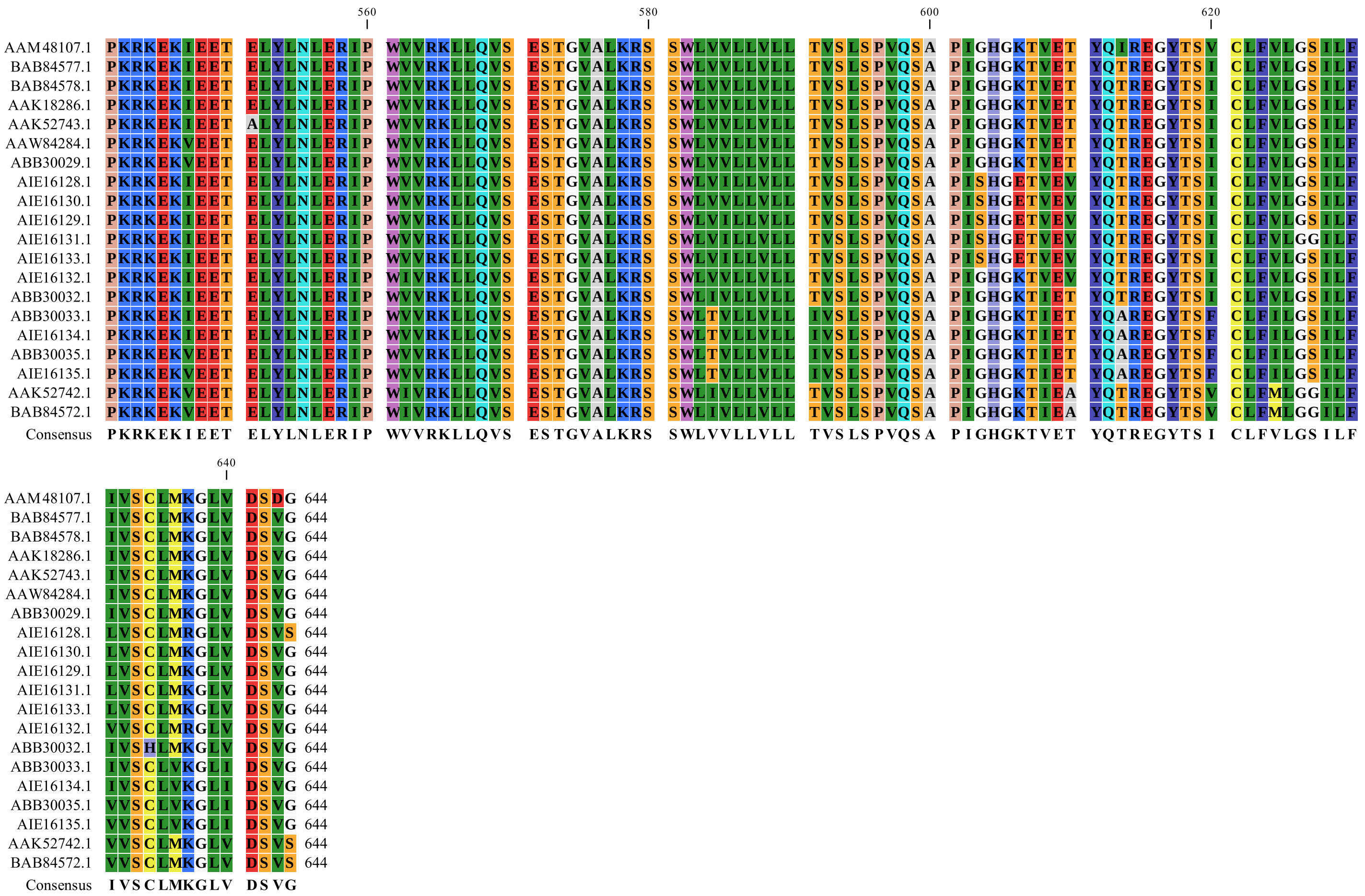
560 580 600 620
AAM48107.1 PKRKEKIEET ELYLNLERIP WVVRKLLQVS ESTGVALKRS SWLVVLLVLL TVSLSPVQSA PIGHGKTVET YQIREGYTSV CLFVLGSILF
BAB84577.1 PKRKEKIEET ELYLNLERIP WVVRKLLQVS ESTGVALKRS SWLVVLLVLL TVSLSPVQSA PIGHGKTVET YQTREGYTSI CLFVLGSILF
BAB84578.1 PKRKEKIEET ELYLNLERIP WVVRKLLQVS ESTGVALKRS SWLVVLLVLL TVSLSPVQSA PIGHGKTVET YQTREGYTSI CLFVLGSILF
AAK18286.1 PKRKEKIEET ELYLNLERIP WVVRKLLQVS ESTGVALKRS SWLVVLLVLL TVSLSPVQSA PIGHGKTVET YQTREGYTSI CLFVLGSILF
AAK52743.1 PKRKEKIEET ALYLNLERIP WVVRKLLQVS ESTGVALKRS SWLVVLLVLL TVSLSPVQSA PIGHGKTVET YQTREGYTSI CLFVLGSILF
AAW84284.1 PKRKEKVEET ELYLNLERIP WVVRKLLQVS ESTGVALKRS SWLVVLLVLL TVSLSPVQSA PIGHGKTVET YQTREGYTSI CLFVLGSILF
ABB30029.1 PKRKEKVEET ELYLNLERIP WVVRKLLQVS ESTGVALKRS SWLVVLLVLL TVSLSPVQSA PIGHGKTVET YQTREGYTSI CLFVLGSILF
AIE16128.1 PKRKEKIEET ELYLNLERIP WVVRKLLQVS ESTGVALKRS SWLVILLVLL TVSLSPVQSA PISHGETVEV YQTREGYTSI CLFVLGSILF
AIE16130.1 PKRKEKIEET ELYLNLERIP WVVRKLLQVS ESTGVALKRS SWLVVLLVLL TVSLSPVQSA PISHGETVEV YQTREGYTSI CLFVLGSILF
AIE16129.1 PKRKEKIEET ELYLNLERIP WVVRKLLQVS ESTGVALKRS SWLVILLVLL TVSLSPVQSA PISHGETVEV YQTREGYTSI CLFVLGSILF
AIE16131.1 PKRKEKIEET ELYLNLERIP WVVRKLLQVS ESTGVALKRS SWLVILLVLL TVSLSPVQSA PISHGETVEV YQTREGYTSI CLFVLGGILF
AIE16133.1 PKRKEKIEET ELYLNLERIP WVVRKLLQVS ESTGVALKRS SWLVILLVLL TVSLSPVQSA PISHGETVEV YQTREGYTSI CLFVLGSILF
AIE16132.1 PKRKEKIEET ELYLNLERIP WIVRKLLQVS ESTGVALKRS SWLVVLLVLL TVSLSPVQSA PIGHGKTVEV YQTREGYTSI CLFVLGSILF
ABB30032.1 PKRKEKIEET ELYLNLERIP WVVRKLLQVS ESTGVALKRS SWLIVLLVLL TVSLSPVQSA PIGHGKTIET YQTREGYTSI CLFVLGSILF
ABB30033.1 PKRKEKIEET ELYLNLERIP WVVRKLLQVS ESTGVALKRS SWLTVLLVLL IVSLSPVQSA PIGHGKTIET YQAREGYTSF CLFILGSILF
AIE16134.1 PKRKEKIEET ELYLNLERIP WVVRKLLQVS ESTGVALKRS SWLTVLLVLL IVSLSPVQSA PIGHGKTIET YQAREGYTSF CLFILGSILF
ABB30035.1 PKRKEKVEET ELYLNLERIP WVVRKLLQVS ESTGVALKRS SWLTVLLVLL IVSLSPVQSA PIGHGKTIET YQAREGYTSF CLFILGSILF
AIE16135.1 PKRKEKVEET ELYLNLERIP WVVRKLLQVS ESTGVALKRS SWLTVLLVLL IVSLSPVQSA PIGHGKTIET YQAREGYTSF CLFILGSILF
AAK52742.1 PKRKEKVEET ELYLNLERIP WIVRKLLQVS ESTGVALKRS SWLIVLLVLL TVSLSPVQSA PIGHGKTIEA YQTREGYTSV CLFMLGGILF
BAB84572.1 PKRKEKVEET ELYLNLERIP WIVRKLLQVS ESTGVALKRS SWLIVLLVLL TVSLSPVQSA PIGHGKTIEA YQTREGYTSV CLFMLGGILF
Consensus PKRKEKIEET ELYLNLERIP WVVRKLLQVS ESTGVALKRS SWLVVLLVLL TVSLSPVQSA PIGHGKTVET YQTREGYTSI CLFVLGSILF
640
AAM48107.1 IVSCLMKGLV DSDG 644
BAB84577.1 IVSCLMKGLV DSVG 644
BAB84578.1 IVSCLMKGLV DSVG 644
AAK18286.1 IVSCLMKGLV DSVG 644
AAK52743.1 IVSCLMKGLV DSVG 644
AAW84284.1 IVSCLMKGLV DSVG 644
ABB30029.1 IVSCLMKGLV DSVG 644
AIE16128.1 LVSCLMRGLV DSVS 644
AIE16130.1 LVSCLMKGLV DSVG 644
AIE16129.1 LVSCLMKGLV DSVG 644
AIE16131.1 LVSCLMKGLV DSVG 644
AIE16133.1 LVSCLMKGLV DSVG 644
AIE16132.1 VVSCLMRGLV DSVG 644
ABB30032.1 IVSHLMKGLV DSVG 644
ABB30033.1 IVSCLVKGLI DSVG 644
AIE16134.1 IVSCLVKGLI DSVG 644
ABB30035.1 VVSCLVKGLI DSVG 644
AIE16135.1 VVSCLVKGLI DSVG 644
AAK52742.1 VVSCLMKGLV DSVS 644
BAB84572.1 VVSCLMKGLV DSVS 644
Consensus IVSCLMKGLV DSVG

C. GP2 protein of Congo virus Asia-1 genotype multiple sequence alignment among the genomes retrieved from NCBI with accession numbers AAM48107, AAK52742, BAB84572, AIE16134, ABB30033, AIE16135, ABB30035, ABB30032, AIE16129, AIE1612B8, AIE16130, AIE16131, AIE16133, AIE16132, AAW84284, AAK52743, ABB30029, BA84577, AF338470 and BAB84578.

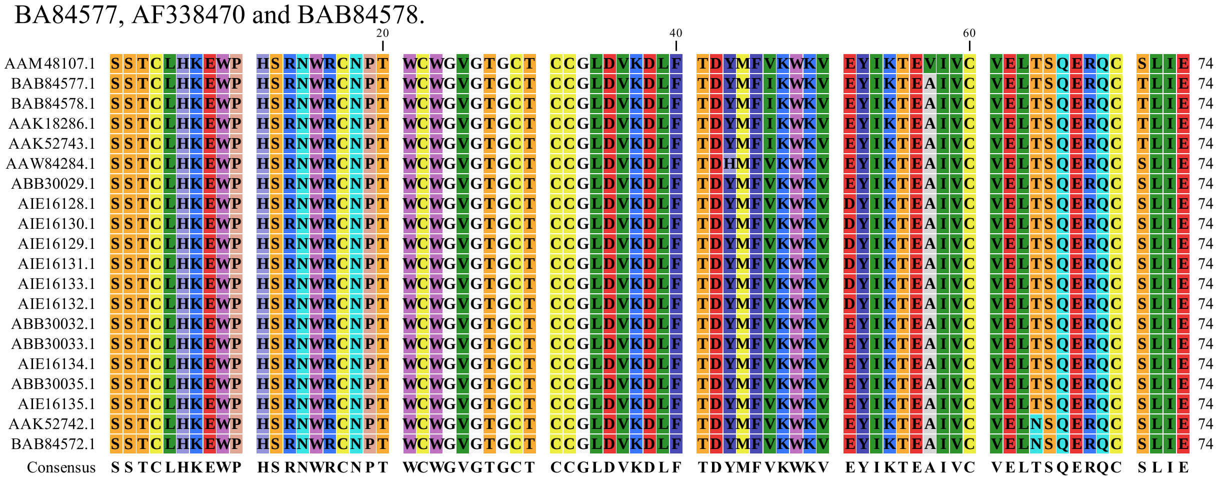

Supplement: Supplementary file 1 [file vaccines-11-00061-s001.zip › Supplimentry File 2.pdf]
